# Receptor-binding proteins from animal viruses are broadly compatible with human cell entry factors

**DOI:** 10.1038/s41564-024-01879-4

**Published:** 2025-01-02

**Authors:** Jérémy Dufloo, Iván Andreu-Moreno, Jorge Moreno-García, Ana Valero-Rello, Rafael Sanjuán

**Affiliations:** Institute for Integrative Systems Biology, Universitat de València - Consejo Superior de Investigaciones Científicas, Paterna, Spain

**Keywords:** Viral transmission, Virus-host interactions, Viral membrane fusion, Systems virology

## Abstract

Cross-species transmission of animal viruses poses a threat to human health. However, systematic experimental assessments of these risks remain scarce. A critical step in viral infection is cellular internalization mediated by viral receptor-binding proteins (RBPs). Here we constructed viral pseudotypes bearing the RBPs of 102 enveloped RNA viruses and assayed their infectivity across 5,202 RBP–cell combinations. This showed that most of the tested viruses have the potential to enter human cells. Pseudotype infectivity varied widely among the 14 viral families examined and was influenced by RBP characteristics, host of origin and target cell type. Cellular gene expression data revealed that the availability of specific cell-surface receptors is not necessarily the main factor limiting viral entry and that additional host factors must be considered. Altogether, these results suggest weak interspecies barriers in the early stages of infection and advance our understanding of the molecular interactions driving viral zoonosis.

## Abstract (web summary)

Functional systematic pseudotype virus-based analysis of the ability of diverse animal viruses to enter into a panel of well-characterized human cell lines shows a broad compatibility between animal virus receptor-binding proteins and human cell entry factors.

## Main

The cross-species transmission of animal viruses to humans (zoonosis) poses a tremendous threat to human health. A substantial fraction of the estimated tens of thousands of viruses infecting wildlife or domestic mammals may spill over into humans^1^. However, predicting which viruses are more likely to emerge is extremely challenging, as this is a largely random process influenced by many ecological, evolutionary, social, genetic and virological factors. Previous studies have identified ecological risk factors including biodiversity loss^2^ and global warming^3^ among other disturbances^4,5^, as well as important targets of evolutionary optimization following viral cross-species transmission^6^. Certain viral traits have also been associated with zoonotic risk. In particular, enveloped RNA viruses are of greatest concern as they show increased cross-species transmissibility and account for >70% of all zoonotic viruses^7–10^.

Recent large-scale viral metagenomics initiatives have led to major advances in the identification of potential zoonotic threats^11^. However, experimental virology studies providing functional information about the ability of animal viruses to infect human cells are comparatively scarce^12^ because viral culturing is technically challenging and raises biosafety issues. One way to circumvent these limitations is to use surrogate systems that recapitulate specific steps of the infection cycle, such as viral pseudotypes, in which the receptor-binding protein (RBP) of an enveloped virus of interest is incorporated into a viral vector. Pseudotyping has been applied to most families of enveloped RNA viruses and can faithfully reproduce key processes such as receptor usage, cellular tropism and antibody-mediated neutralization^13^.

The cross-species transmission of viruses initially depends on the compatibility between the viral RBP and the cellular entry factors of different host species. Interspecies variability in these factors has often been considered a major barrier to zoonosis, based on observations made with a few well-studied viruses, such as avian and human influenza strains^14^ or coronaviruses^15^. Moreover, an evolutionary arms race between RBPs and virus receptors has been shown in several cases including some rodent arenaviruses^16^, bat ebolaviruses^17^ and hominid lentiviruses^18^. However, viral internalization is a complex process that typically involves multiple steps including initial attachment^19^, receptor binding, endocytosis pathways and antiviral proteins acting at the entry stage of infection^20^. How these factors determine the infection of human cells remains unknown for most viruses.

Using available RBP sequences from animal viruses, the combination of gene synthesis and pseudotyping allows us to systematically study the ability of these viruses to enter human cells and the molecular determinants of this process. To address this goal, we engineered pseudotypes carrying the RBPs from over a hundred viruses belonging to 14 different families of enveloped RNA viruses and tested them in dozens of well-characterized human cell lines. We detected viral entry in most RBP–cell combinations tested. Pseudotype infectivity varied strongly across viral families, with coronaviruses showing the strongest interspecies barrier at the entry stage. Using information on viral taxonomy, viral host and the cell type being challenged, we explored the predictability of RBP-mediated human cell internalization. We also showed that specific RBP–receptor interactions are not always a limiting step for infection, and we identified host factors with broad-range effects on viral internalization and cellular tropism.

## Results

### The RBPs from animal RNA viruses frequently allow entry into human cells

We built RBP phylogenetic trees for 14 families of enveloped RNA viruses (Supplementary Figs. 1–14**)** and used them to select 129 RBPs that spanned the diversity of each family (Supplementary Table 1). We obtained these RBPs through gene synthesis and used vesicular stomatitis virus (VSV) as a system to produce viral pseudotypes. Where necessary and feasible, pseudotypes also incorporated other viral proteins involved in entry in addition to the RBP (for example, E1–E2 for flaviviruses, togaviruses and matonaviruses, F and G for paramyxoviruses; Supplementary Table 1). Pseudotypes were successfully constructed for 102 RBPs, as shown by western blot analysis of viral particles or preliminary infectivity assays (Supplementary Figs. 1–14). Pseudotyping success rates were similarly high among viral families, except for flaviviruses, which showed low pseudotyping efficiency (Supplementary Fig. 15**)**. Of the 102 RBPs, 78 corresponded to viruses not reported to infect humans, including 50 viruses described in different mammals such as bats, rodents or artiodactyls, among others, 6 viruses described in non-mammalian vertebrates (birds or fish) and 22 viruses reported only in arthropods but belonging to families that contain arboviruses (Supplementary Fig. 16 and Supplementary Table 2). Among the 24 known human-infective viruses, 21 were zoonotic, and 3 were typically human exclusive (rubella, hepatitis C and measles viruses). We also explored available information on previous cell culture propagation of each virus. In addition to the 24 viruses known to infect humans, 14 viruses have been passaged in at least one human cell line, whereas 37 have been passaged in non-human cells, and 27 have not been cultured so far (Supplementary Fig. 17 and Supplementary Table 3).

**Extended Data Fig. 1:**
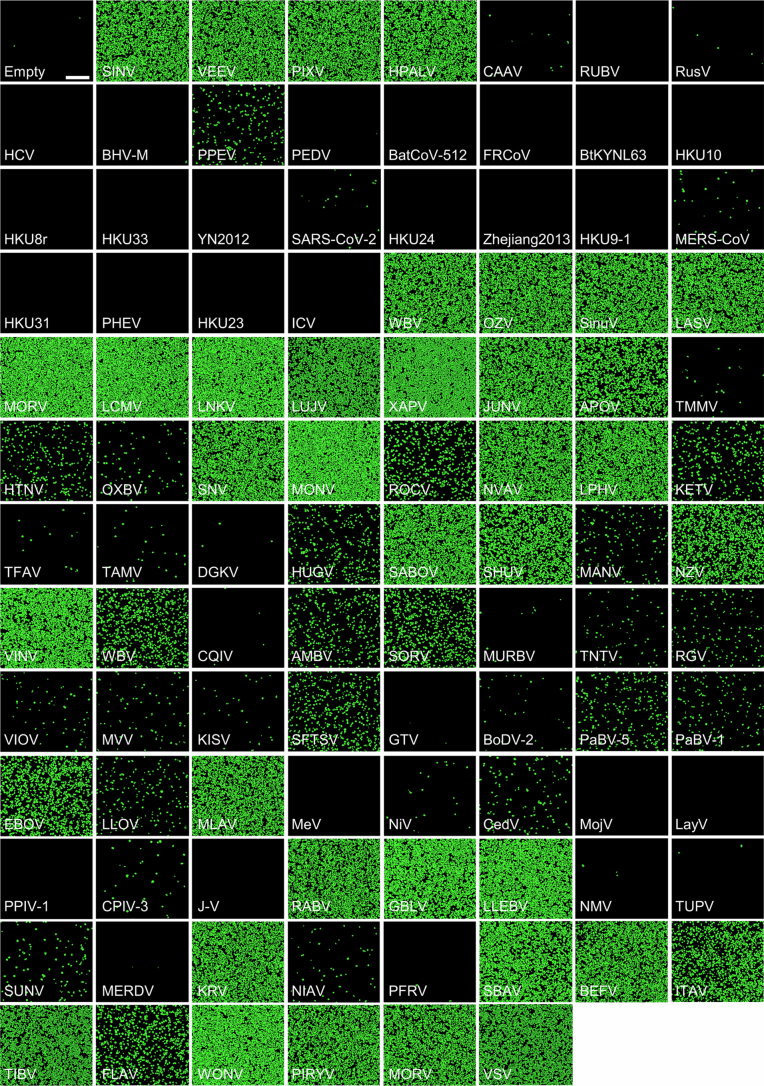
Assay of RBP-mediated viral entry in HEK293T cells. HEK293T cells were inoculated with VSV pseudotypes and imaged after 24 h with the SX5 Live-Cell Analysis System at a 4X magnification. Images show the GFP-positive area defined using the Incucyte Analysis software. Virus abbreviations are shown. Top left: empty control in which cells were inoculated with a VSV carrying no RBP. Scale bar: 400 µm. Data are provided in Supplementary Table 4. The experiment was performed once. Source data

**Extended Data Fig. 2:**
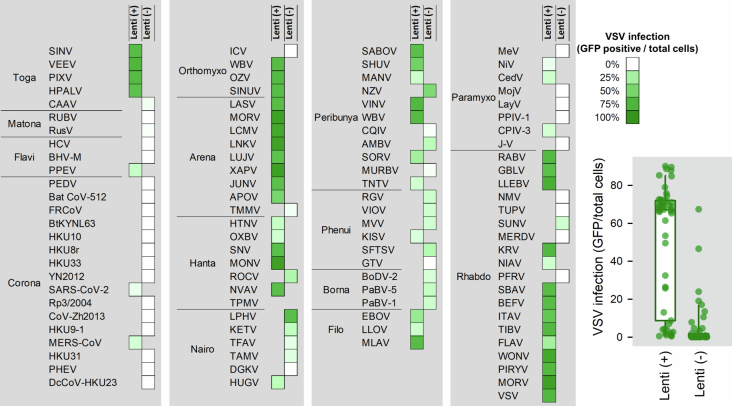
RBP-mediated viral entry in HEK293T cells for VSV versus lentiviral pseudotypes. Shades of green correspond to the images shown in Extended Data Fig. 1. For each RBP, lentiviral infection was ascertained qualitatively (+ or −) based on visual comparison of the GFP signal with the background obtained with an empty control. Right: box plot of VSV infection data as a function of whether RBP-mediated entry was detected in the lentivirus system. Boxes show the median (green line), 25th and 75th percentiles. Vertical lines departing from boxes indicate the 10th and 90th percentiles. Dots show data points for individual RBPs (n = 102). Source data

To preliminarily test whether RBPs could mediate viral entry into human cells, we inoculated HEK293T cells with each pseudotype. For 74 out of 102 RBPs, pseudotypes showed infectivity values above the background levels obtained with an empty control (that is, VSV carrying no RBP) as determined by VSV-encoded GFP (green fluorescent protein) signal, showing that most RBPs mediated viral internalization (Extended Data Fig. 1 and Supplementary Table 4). We also used lentiviruses as an alternative pseudotyping system^21^, but this proved to be a less sensitive method as fluorescence was weaker and more difficult to quantify. Despite this limitation, we detected infection for 52 out of 102 RBPs and found consistency between the two pseudotyping systems. All RBPs that yielded infection signal with lentiviruses did so with VSV, while those showing no signal with lentiviruses tended to show weak or no signal with VSV (Extended Data Fig. 2 and Supplementary Table 4). To examine another cell line, we inoculated primary human umbilical vein endothelial cells (HUVEC) with the 102 VSV-based pseudotypes. Although infection was generally weaker due to lower VSV susceptibility (5% GFP-positive cells versus 72% in HEK293T cells inoculated with full VSV control), we still detected viral internalization for 43 out of 102 RBPs (Supplementary Table 4).

**Fig. 1:**
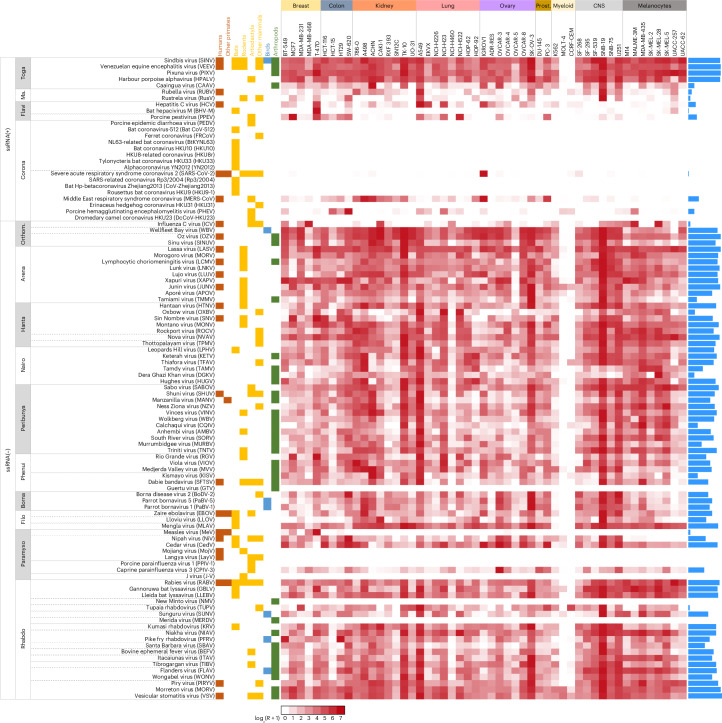
Tropism of RBPs from 102 enveloped RNA viruses in 51 human cell lines. Viruses are sorted by family and organized by genera within families, as indicated by the horizontal lines. Orange, yellow and green boxes show whether each virus has been reported to infect humans, other groups of mammals and arthropods, respectively. Names of the 51 cell lines are indicated at the top, and cells are organized by tissue of origin. The heat map shows relative pseudotype infectivity, calculated as log_2_(*R* + 1), where *R* is the MOI scaled as a percentage of the maximum MOI across all cell lines for each pseudotype (see Methods for details). Blue bars on the right indicate the number of cell lines in which infection was detected. ssRNA, single-stranded RNA; Ma., Matonaviridae; Orthom., Orthomyxoviridae; Prost., prostate. Source data

To study the infectivity of VSV pseudotypes in human cells more systematically, we extended our experiments to 51 cell lines from the NCI-60 panel (Supplementary Table 5). We verified that the average expression of cell-surface genes in these cell lines correlated with the values reported for normal tissues in the Human Protein Atlas (Pearson *r* = 0.841, *P* < 0.0001) and that 51 cell lines was a sufficient sample size to saturate detection of infectious pseudotypes (Supplementary Fig. 18). Out of the 102 × 51 = 5,202 total RBP–cell pairs tested, 2,698 (51.9%) showed viral internalization, and 82 out of 102 RBPs (80.4%) showed internalization in at least one of the 51 cell lines (Fig. 1 and Supplementary Table 6). We conclude that the RBPs of most animal viruses tested are compatible with human cell entry factors.

**Fig. 2:**
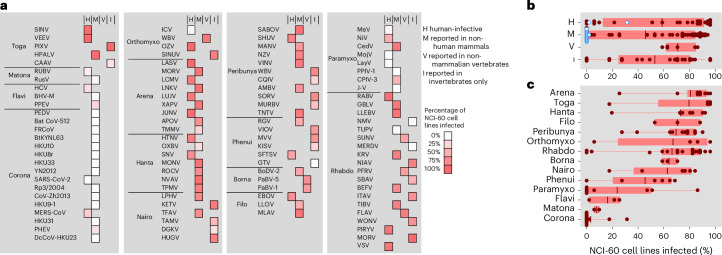
Percentage of cell lines infected as a function of viral family and host type. **a**, For each pseudotype, the percentage of NCI-60 cell lines in which infection was detected is shown in shades of red. Viruses are classified by family and in four groups according to their reported hosts: human-infective (H), non-human mammals (M), non-mammalian vertebrates (V) or invertebrates only (I). **b**, Box plot of the percentage of NCI-60 cell lines infected for each of these four groups. Coronaviruses are indicated in blue. **c**, Box plot of the percentage of NCI-60 cell lines infected by viral family. In **b** and **c**, boxes show the median (red line) and 25th and 75th percentiles, and horizontal lines departing from boxes indicate the 10th and 90th percentiles. Dots show data points for individual RBPs (*n* = 102). Infectivity as a function of previous viral passaging evidence is provided in Supplementary Fig. 19. Source data

The weakness of the interspecies barrier at the entry stage was evident for the RBPs of most viruses regardless of whether they have been reported to infect humans, non-human mammals, other vertebrates or only arthropods in nature (Fig. 2a,b; two-way analysis of variance (ANOVA) controlling for viral family, *P* > 0.10). Examples of viruses not previously reported in mammals but whose RBPs mediated human cell internalization include Sinu virus, a thogotovirus isolated in 2017 from mosquitoes in Colombia^22^; Wellfleet Bay virus, a quaranjavirus identified in 2014 as the causative agent of avian mass mortality in the United States^23^; and Niakha virus, a rhabdovirus isolated in 2013 from phlebotomine sandflies in Senegal^24^. Examples of viruses reported in non-human mammals include the harbour porpoise alphavirus, isolated in 2021 in Alaska from cetaceans^25^, and Rustrela virus, a relative of Rubella virus discovered in 2020 as the causative agent of brain infection in wild yellow-necked field mice and zoo animals^26^, for which we found RBP-mediated entry into human astrocyte- and lung-derived cells. Concerning known human-infective viruses, one of the most infectious pseudotypes carried the RBP of Oz virus, a zoonotic thogotovirus that recently caused the first human fatality in Japan^27^. Infection rates >50% were also observed for the pseudotypes of Shuni orthobunyavirus, a suspected cause of human neurological disease in Africa^28^; Manzanilla orthobunyavirus, which is maintained in nature through a pig–mosquito–bird cycle in Africa and South East Asia^29^; Dabie bandavirus, a highly pathogenic emerging bunyavirus responsible for the severe fever with thrombocytopaenia syndrome in East Asia^30^; and several arenaviruses and rhabdoviruses.

### Viral and host features impact RBP-mediated human cell entry

We then explored whether information about virus taxonomy, general RBP features, reported hosts and cells could be used to predict the ability of RBPs to mediate internalization. The most obvious effect corresponded to viral taxonomy (Fig. 2c). Coronavirus, matonavirus and flavivirus pseudotypes showed the narrowest tropism, infecting only 3.2%, 7.8% and 16.3% of the 51 cell lines, respectively. In the case of flaviviruses, inefficient pseudotype production and poor RBP incorporation could be responsible for this narrow tropism (Supplementary Fig. 3). However, matonavirus and coronavirus RBPs were incorporated well into pseudotypes (Supplementary Figs. 2 and 4). Coronaviruses comprised 13 of the 20 pseudotypes showing a complete lack of infectivity. Moreover, the three coronavirus pseudotypes for which we detected infection entered only 1, 9 and 16 of the 51 cell lines. For example, the severe acute respiratory syndrome coronavirus 2 (SARS-CoV-2) spike only mediated entry into IGROV-1 cells, as shown previously^31^. By contrast, pseudotypes from the other 11 viral families infected most cell lines, with the highest percentages corresponding to arenaviruses (80.8 ± 7.3%), togaviruses (79.6 ± 15.0%) and hantaviruses (73.1 ± 9.3%).

**Fig. 3:**
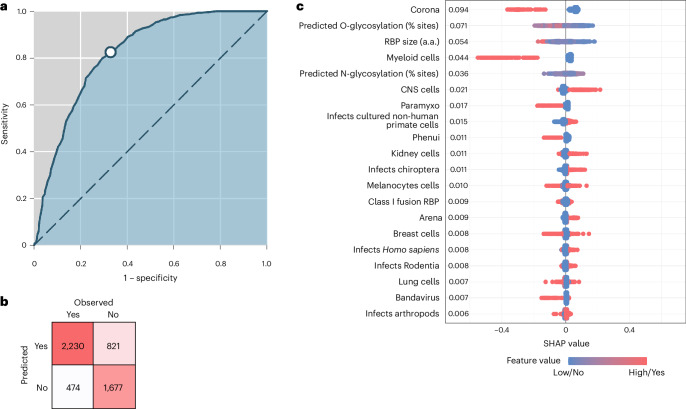
Gradient boosting model for the prediction of RBP-mediated human cell entry. **a**, AUC plot showing the true positive rate (TPR, that is, fraction of infections correctly predicted by the model, also termed sensitivity or recall) versus the false positive rate (FPR, that is, fraction of real negative infections erroneously predicted as positives by the model, or 1 − specificity). The white dot indicates the values obtained when a model score equal to 0.5 was used as threshold value for making predictions. **b**, Confusion matrix. Counts of model predictions for the presence/absence or infection versus data labels are shown. **c**, SHAP summary plot showing the impact of the top 20 features on the model predictions. Each dot represents a data point, coloured based on its feature value (low/high for continuous features, no/yes for binary features). The numbers on the left represent the sum of the absolute SHAP values for each feature, indicating its overall importance in the predictions. SHAP plots for individual viruses are provided in Supplementary Fig. 20. a.a., number of amino acids. Source data

**Extended Data Fig. 3:**
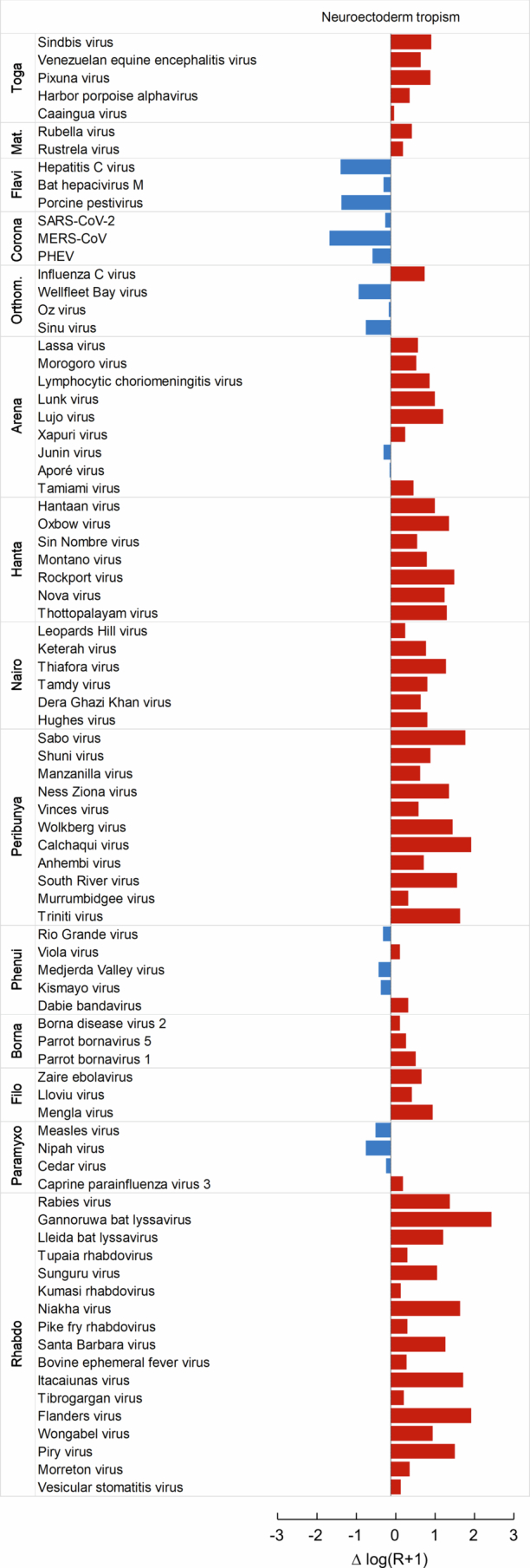
Tropism of pseudotypes for neuroectoderm-derived cells. For each pseudotype, the difference between the average log_2_(*R* + 1) values in the 15 cell lines derived from the neuroectoderm versus the other 36 cell lines is shown. Red and blue bars show RBPs with a preference for neuroectoderm-derived cells versus other cells, respectively. Source data

We used gradient boosting to examine the ability of 81 features to collectively predict RBP-mediated internalization in NCI-60 cells (Fig. 3). The model showing the highest cross-validated performance reached an area under the curve (AUC) of 81.6%, a sensitivity of 82.5% and a precision of 73.1% (Fig. 3a). Overall, 75.1% of the total 5,202 RPB–cell combinations were correctly predicted (Fig. 3b). Shapley additive explanations (SHAP) showed that the most important feature was the viral family, particularly for coronaviruses, which showed a very narrow cell tropism (Fig. 3c). RBP features such as protein glycosylation and size were additional relevant features, albeit there was no straightforward relationship between these features and RBP tropism (Supplementary Fig. 20). RBPs from viruses previously propagated in cell cultures were more likely to mediate entry into the NCI-60 cells examined. It is worth noting that the model detected a similar positive effect on infectivity for RBPs from bat viruses. We also found that cells derived from the central nervous system (CNS) tended to be more susceptible to infection by the pseudotypes tested, whereas myeloid cell lines were the least susceptible. The poor infection of myeloid cells may be attributed to the VSV vector used, which did not infect these cells robustly. By contrast, VSV showed no marked preference for CNS cells or, more broadly, neuroectoderm-derived cells, whereas most Bunyavirales and rhabdovirus pseudotypes showed such preference (Extended Data Fig. 3).

**Fig. 4:**
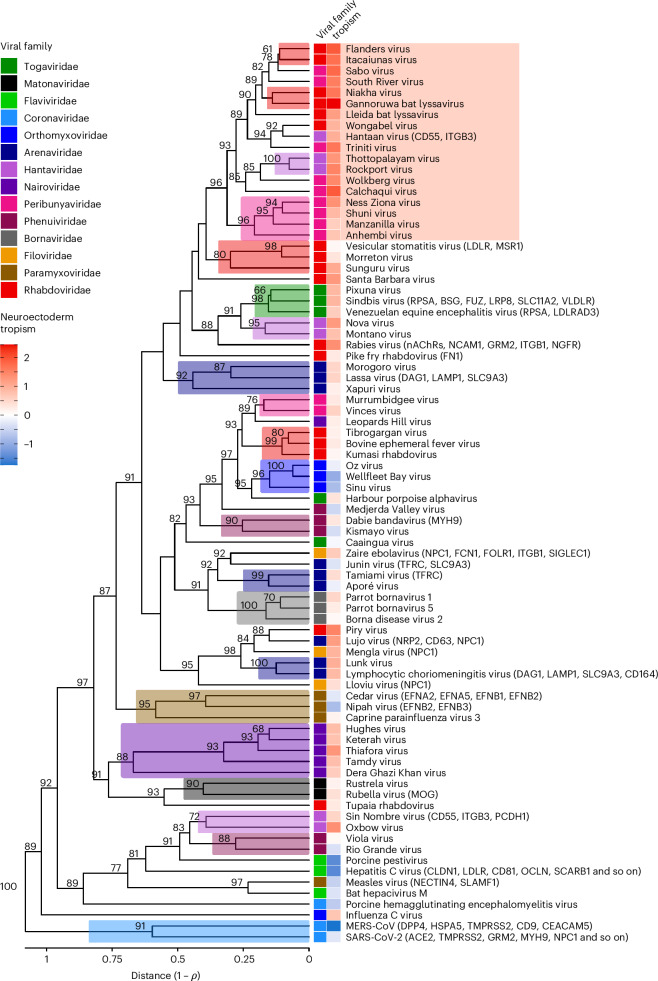
Hierarchical cluster analysis of pseudotype infectivity profiles across cell lines. Dendrogram of the 82 pseudotypes infecting at least one cell line, built using WPGMA on the Pearson correlation distance matrix derived from the infectivity correlation matrix between pairs of pseudotypes shown in Supplementary Fig. 21. Shades above branches indicate clusters of pseudotypes formed exclusively by members of the same viral family. Numbers in nodes indicate approximately unbiased bootstrap values >50%. The viral family of each virus and its tropism towards neuroectoderm-derived cells (also shown in Extended Data Fig. 3) are displayed. This tropism was calculated as the difference between the average log_2_(*R* + 1) values obtained in neuroectoderm-derived versus all other cell lines. The names of known viral receptors are shown in parentheses (see Fig. 5 for a more complete list). The red rectangle on the right highlights a cluster of RBPs showing a significantly higher preference towards neuroectoderm-derived cells (two-sided Wilcoxon rank-sum test, *P* < 0.0001).

We then performed a hierarchical clustering analysis to test whether RBPs from the same viral family showed similar NCI-60 infectivity profiles. We obtained 20 clusters of 2–7 RBPs formed exclusively by members of the same family (Fig. 4 and Supplementary Fig. 21), and phylogenetically consistent clusters were found among orthobornaviruses, vesiculoviruses, non-influenza orthomyxoviruses, henipaviruses, bandaviruses, matonaviruses and most nairoviruses, suggesting that viruses within these groups use similar entry factors. However, in many cases, clusters did not include all members of a given taxonomical group. We identified preference towards neuroectoderm-derived cells as an additional factor driving the observed clustering. This tropism was particularly marked in a well-delimited cluster formed by nine peribunyavirus, six rhabodvirus, and three hantavirus RBPs (Wilcoxon rank-sum test: *P* < 0.0001), including those from viruses known to cause brain pathology, such as Gannoruwa bat lyssavirus. By contrast, coronavirus and flavivirus pseudotypes rarely entered neuroectoderm-derived cells (0.4% and 0.0% cell lines, respectively), whereas epithelial cells were more frequently infected (4.2% and 22.5%, respectively; Fisher exact tests: *P* = 0.002 and *P* < 0.0001, respectively; Extended Data Fig. 3).

### Influence of known receptors on human cellular tropism

We observed similar pseudotype infectivity profiles among some RBPs known to share cellular receptors, such as those of Nipah and Cedar viruses, as well as those of Sindbis virus and Venezuelan equine encephalitis virus (VEEV; Fig. 4). However, this was not always the case. For instance, Lassa virus and lymphocytic choriomeningitis virus (LCMV) are two Old-World arenaviruses that use dystroglycan-1 (DAG1) as a receptor^32^, but their infectivity across cell lines correlated poorly, and the profile of the LCMV pseudotype was more similar to that of Lujo arenavirus, which uses neuropilin 2 (NRP2) and cluster of differentiation (CD) 63 instead^33^.

**Fig. 5:**
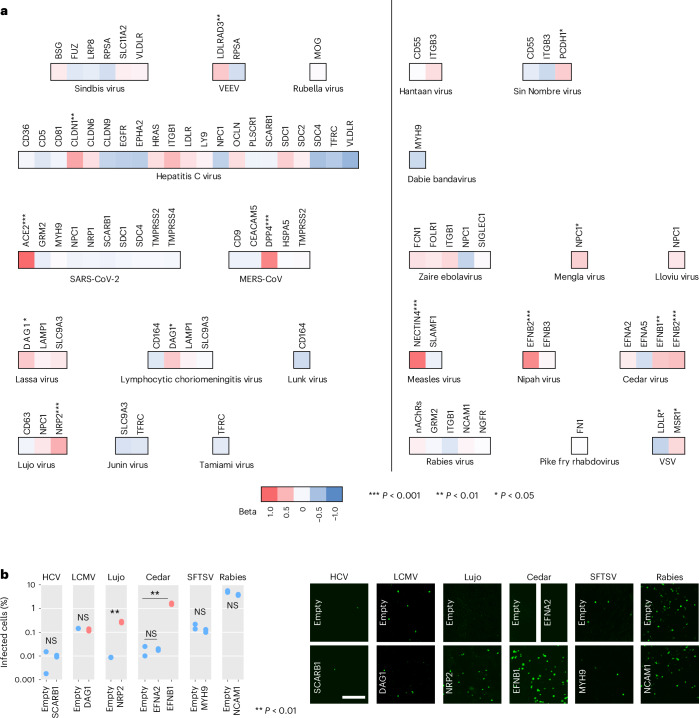
Contribution of the expression level of known receptors to RBP cellular tropism. **a**, Multiple regression analysis of pseudotype infectivity as a function of the RNA-seq expression levels of known viral receptors. For each virus, colours indicate the standardized regression coefficient. Significant coefficients are indicated with asterisks (ANOVA; SARS-CoV-2–ACE2, MERS-CoV–DPP4, measles–NECTIN4, Nipah–EFNB2: *P* < 0.0001; VEEV–LDLRAD3: *P* = 0.0056, HCV–CLDN1: *P* = 0.0042; Lassa–DAG1: *P* = 0.0120; LCMV–DAG1: *P* = 0.0103; Lujo–NRP2: *P* = 0.0001; Sin Nombre–PCDH1: *P* = 0.0320; Mengla–NPC1: *P* = 0.0193; Cedar–EFNB1: *P* = 0.0032; Cedar–EFNB2: *P* = 0.0004; VSV–LDLR: *P* = 0.0166; VSV–MSR1: *P* = 0.0330). Lectins and proteins that directly or indirectly attach to lipids were not included because we focused on proteins interacting with RBPs. For the rabies virus pseudotype, the reads per kilobase per million (rpkm) values of all genes encoding cholinergic receptor nicotinic (nAChRs) Alpha or Beta subunits were summed. Scatter plots for several significant cases are shown in Extended Data Fig. 4. **b**, Effect of receptor overexpression on viral entry. Cell lines were transfected with a receptor-encoding plasmid or an empty vector and then inoculated with pseudotypes. The most appropriate cell line was chosen for each pseudotype–receptor pair based on the criteria detailed in the Methods section. These were SK-OV-3 for HCV–SCARB1, MCF7 for LCMV–DAG1 and Lujo–NRP2, ACHN for Cedar–EFNA2 and Cedar–EFNB1, UACC-62 for SFTSV–MYH9, and EKVX for rabies–NCAM1. The percentage of infection at 24 h post inoculation is shown. Two technical replicates (*n* = 2) were carried out for each assay. Data points shown in blue and red correspond to receptors with non-significant and significant effects on pseudotype infectivity according to the multiple linear regression shown above, respectively. Experimental results were compared with the empty controls using two-sided *t*-tests. **P < 0.01; NS, not significant (Lujo: *P* = 0.0051, Cedar–EFNB1: *P* = 0.0044). Representative images are shown on the right. Scale bar, 400 µm. Source data

**Extended Data Fig. 4:**
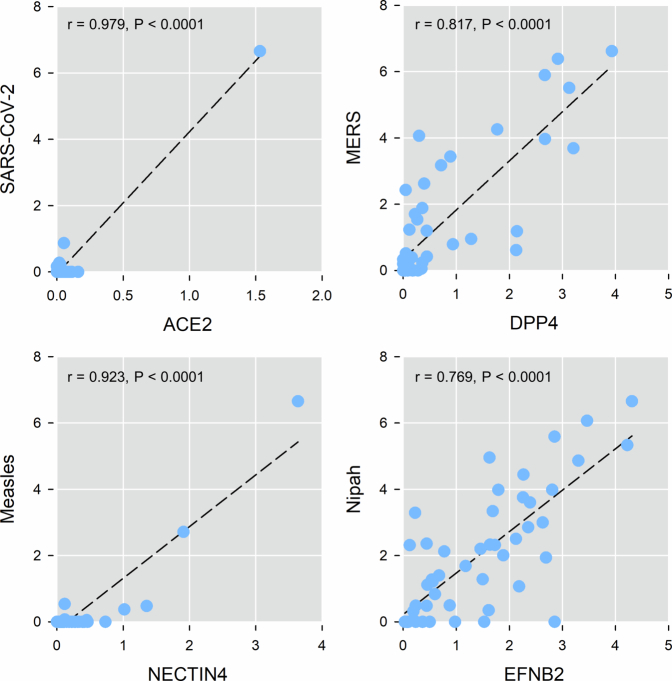
Examples of significantly positive correlations between the expression of known receptors and pseudotyped virus infectivity. Correlation between the expression of receptor genes, measured by RNA-seq as log_2_(rpkm+1), and viral pseudotypes infectivity, measured as log_2_(*R* + 1) across 50 cell lines. Each dot represents a cell line. Pearson r coefficients and *P* values are indicated (two-sided test, all *P* values < 0.0001). Source data

We used multiple linear regression to analyse how pseudotype infectivity depended on the expression levels of specific receptors, which have been quantified by RNA sequencing (RNA-seq) for 50 of the NCI-60 cell lines^34^. This included primary receptors but also other cell-surface proteins known to interact with RBPs. In some cases, the messenger RNA (mRNA) levels of known receptors explained viral entry satisfactorily, such as angiotensin converting enzyme (ACE) 2 for SARS-CoV-2, nectin 4 for measles, dipeptidyl peptidase (DPP) 4 for MERS-CoV (Middle East respiratory syndrome coronavirus), and Ephrin (EFN) B2 for Nipah virus pseudotypes (*P* < 0.001 in all cases; Fig. 5a and Extended Data Fig. 4). Weaker but significant associations were observed for other receptor–pseudotype combinations, such as LDLRAD3 and VEEV, DAG1 and Lassa and LCMV, NPC1 and Mengla virus, and PCDH1 and Sin Nombre virus. Where available, quantitative proteomic data^35,36^ tended to confirm the associations observed with RNA-seq data (Supplementary Fig. 22).

For viruses with several known receptors, we could identify those more strongly associated with the observed RBP tropism. Specifically, the infection profile of the Lujo virus pseudotype was better explained by the mRNA levels of the NRP2 receptor (*P* < 0.001) than those of the downstream entry factor CD63. Similarly, infection with the Cedar virus pseudotype was positively associated with the expression of EFNB1 and EFNB2 (*P* < 0.01), but not of EFNA2 or EFNA5. This confirms previously published data showing that the affinity of the Cedar virus G protein is much higher for EFNB1 and EFNB2 than for EFNA2 or EFNA5, although all have been described as receptors^37^. The infectivity of LCMV pseudotype correlated slightly more strongly with the mRNA levels of sarcoglycan beta (SCGB) than with those of DAG1, both components of the dystroglycan complex (Supplementary Fig. 23). This suggests that a functional dystroglycan complex may be required for LCMV entry, and that, consequently, SCGB may also function as a determinant of viral tropism.

However, for several other RBP–receptor combinations, the association between receptor expression levels and pseudotype infectivity was weak or absent. As this was unexpected, we set out to verify it experimentally. We transfected expression plasmids encoding different well-established receptors (Supplementary Fig. 24) and quantified the resulting changes in pseudotype infectivity. This included three receptor–RBP pairs for which mRNA levels were significantly associated with infection in the abovementioned analyses (Cedar–EFNB1, Lujo–NRP2 and LCMV–DAG1), versus four with no apparent association (HCV–SCARB1, Cedar–EFNA2, SFTSV–MYH9 and rabies–NCAM1; Fig. 5b). Confirming the results obtained with omics data, only EFNB1 and NRP2 overexpression increased infection by Cedar and Lujo pseudotypes, respectively. The only discrepancy was observed for LCMV and DAG1, for which no effect of receptor overexpression was observed. As shown above, the association between mRNA levels and pseudotype infectivity was modest for this pair, and infectivity also correlated with the expression of another component of the dystroglycan complex. The requirement for a functional dystroglycan complex may explain why overexpression of DAG1 alone was not sufficient to promote viral entry.

### Role of other entry determinants

**Extended Data Fig. 5:**
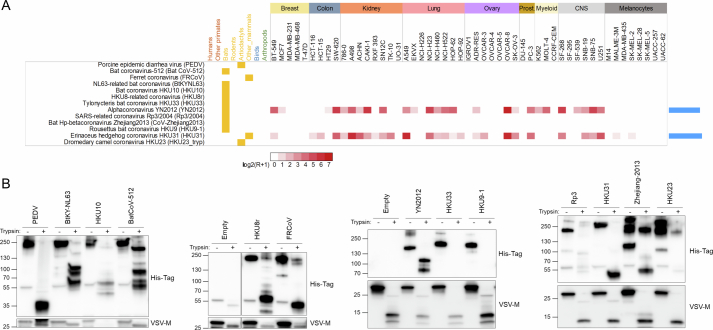
Effect of trypsin treatment on the infectivity of coronavirus pseudotypes. **A**. Viral suspensions were treated with trypsin as indicated, washed and used to inoculate each of the 51 cell lines. Yellow boxes show host information. Names of cell lines are indicated at the top, and cells are organized by tissue of origin. The heat map shows relative pseudotype infectivity, calculated as log_2_(*R* + 1), where R is the multiplicity of infection (MOI) scaled as a percentage of the maximum MOI across all cell lines for each pseudotype. Blue bars on the right indicate the number of cell lines in which infection was detected. **B**. Mock- or trypsin-treated coronavirus pseudotypes were analyzed by western blot. The spike was detected using an anti-His-Tag antibody. VSV-M was used as a loading control. Spike cleavage by trypsin was detected in most cases. Western blot analysis was performed once. Source data

As many virus-specific receptors are currently unknown and the expression of known receptors sometimes did not correlate with pseudotype infectivity, we analysed other entry determinants. For this, we first focused on coronavirus RBPs, which had the narrowest tropism in the human cells tested. It has been shown that coronavirus entry typically requires the involvement of host proteases, which activate spike proteins through cleavage and can play a role in unlocking the cross-species transmissibility of some coronaviruses^15,38,39^. To examine this, we pretreated with trypsin the pseudotypes of the 13 coronaviruses that showed no infectivity, verified spike cleavage by western blot and re-assayed the treated pseudotypes in the 51 cell lines from the NCI-60 panel (Extended Data Fig. 5). This revealed infection of 17 and 18 cell lines from various tissues with the alphacoronavirus YN2012 and Erinaceus hedgehog betacoronavirus HKU31 pseudotypes, respectively. The other 11 pseudotypes remained non-infectious.

**Extended Data Fig. 6:**
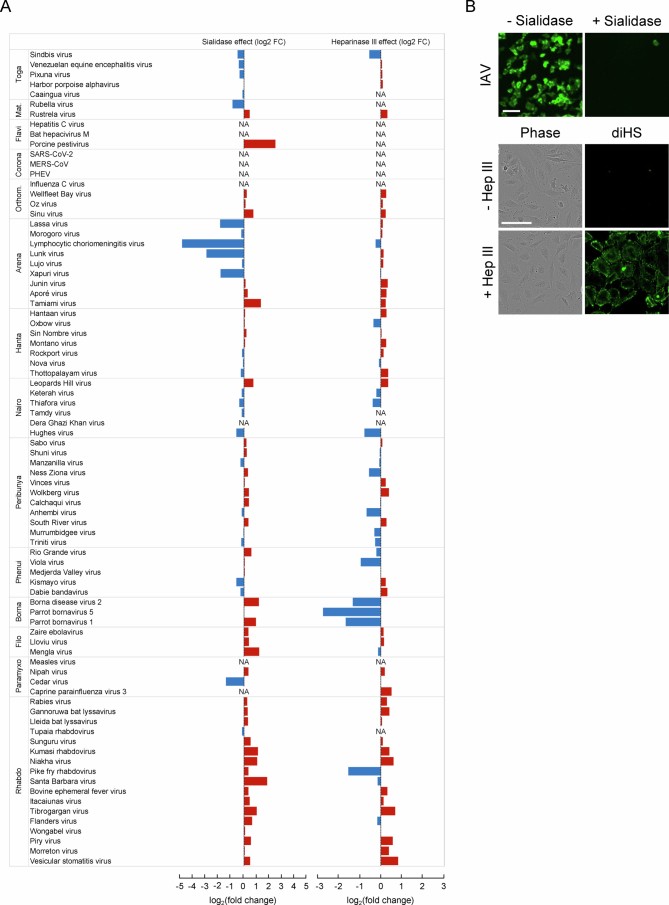
Effect of neuraminidase (sialidase) and heparinase III treatment on pseudotype infectivity in SNB-19 cells. **A**. For each pseudotype, the log_2_(fold change) in the GFP signal in cells pre-treated with neuraminidase (left) or heparinase III (right) relative to untreated controls is shown. Red and blue bars show cases in which treatment increased or decreased infectivity, respectively. All assays were performed twice. NA: not assayed. **B**. Verification of the neuraminidase and Heparinase III activities. Top: reduction in the infectivity of influenza A virus (IAV) PR8–GFP following pretreatment of cells with neuraminidase. Bottom: immunofluorescence validation of heparan sulfate cleavage by Heparinase III using an antibody specific to digested heparan sulfates (diHS). Scale bars: 100 µm. Representative images of one out of two independent experiments are shown. Source data

Carbohydrate moieties serve as attachment factors for many viruses and can be critical for entry, as is for instance the case of most influenza viruses^40^. We focused here on α2,3 and α2,6 sialic acids and heparan sulfate proteoglycans because they are frequently used by viruses and can be easily removed by pre-treatment of cells with exogenous neuraminidase and heparinase, respectively^41,42^. For this, we used SNB-19 (Surgical Neurology Branch-19) cells, as they were susceptible to the largest number of pseudotypes (73/102). We found sialic acid dependence for the RBPs of LCMV (from 66.6 ± 2.0% to 2.24 ± 0.04% GFP-positive cells; *t*-test, *P* = 0.001), other Old-World arenaviruses and, to a lower extent, Cedar virus (Extended Data Fig. 6a). This is in line with work showing that sialic acid can mediate the interactions of CD164 and lysosomal associated membrane protein (LAMP) 1 with the LCMV and Lassa virus glycoproteins, respectively^43,44^. However, removal of sialic acids had no adverse effect for most other pseudotypes and even tended to increase infectivity for some rhabdoviruses (Santa Barbara and Kumasi viruses) and porcine pestivirus.

Although heparinase III treatment efficiently digested heparan sulfates at the surface of SNB-19 cells (Extended Data Fig. 6b), it had little or no effect on the infectivity of most pseudotypes (Extended Data Fig. 6a). A more than twofold reduction in viral entry was observed for the three bornaviruses and pike fry rhabdovirus. Heparan sulfate removal slightly inhibited the infectivity of Sindbis virus, in agreement with previous reports^45^. For some viruses previously shown to use heparan sulfate during viral entry (for example, rabies^46^, Zaire ebolavirus^47^, Nipah^48^), pseudotype infectivity was not affected by heparinase III treatment. These discrepancies may result from the use of different target cells or viral strains, or the use of viral pseudotypes versus full viruses, as adaptation of the latter to cell cultures can favour heparan sulfate usage^42^.

**Fig. 6:**
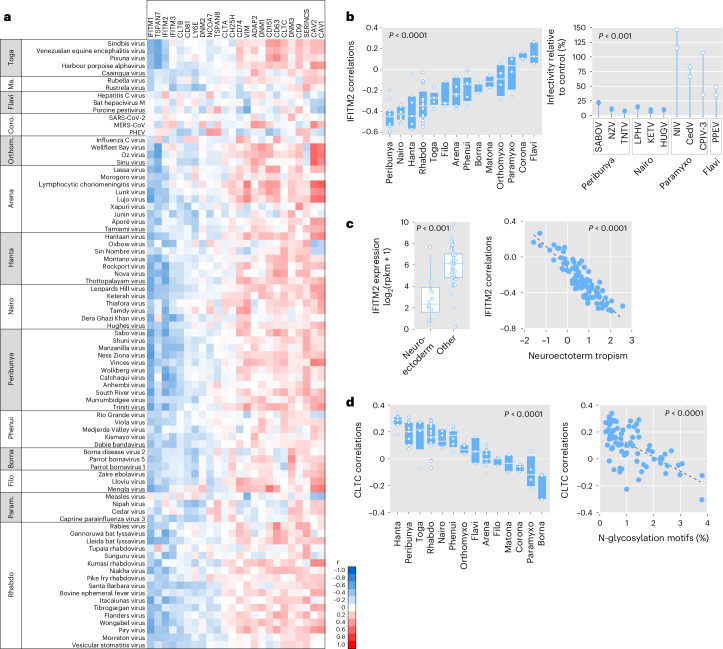
Broad-range determinants of human cell entry across animal viruses. **a**, For 24 host factors known to influence viral entry and the 82 infectious pseudotypes, Pearson correlations between pseudotype infectivity and gene expression levels across cell lines are shown. Infectivity and host gene expression were measured as log_2_(*R* + 1) and log_2_(rpkm + 1), respectively. The heat map indicates positive (red) or negative (blue) correlations, as shown in the scale bar. Ma., Matonaviridae; Coro., Coronaviridae; Param., Paramyxoviridae. **b**, Left: across-family variation in these correlations for IFITM2. Boxes show the median (white line) and 25th and 75th percentiles, and dots show data points for individual RBPs (*n* = 82). Families are sorted according to their mean correlation values. The significance of an ANOVA test for differences among families is shown. Right: effect of IFITM2 overexpression on pseudotype infectivity in HEK293T cells for 10 different RBPs. The percentage of infectivity relative to controls transfected with an empty plasmid is shown. Each dot represents an independent assay (*n* = 2). The peribunyavirus and nairovirus groups showed significantly stronger inhibition by IFITM2 than the flavivirus and paramyxovirus groups (two-sided Wilcoxon test, *P* = 0.00021). Coronavirus and matonavirus pseudotypes were not assayed because they did not infect HEK293T cells. **c**, Left: differential basal IFITM2 expression levels in neuroectoderm-derived cells (two-sided Wilcoxon test, *P* = 0.00012). Boxes show the median and 25th and 75th percentiles. Vertical lines departing from boxes indicate the 10th and 90th percentiles. Dots show data points for individual RBPs. Right: correlation between basal IFITM2 expression and pseudotype infectivity, shown against the RBP preference for neuroectoderm-derived cells, calculated as in Fig. 4 and Extended Data Fig. 3. The Pearson *r* coefficient and *P* values are indicated (two-sided test; *n* = 50 cell lines). **d**, Left: across-family variation in the correlation between CLTC expression and pseudotype infectivity. Families are sorted according to their mean correlation values. Boxes show the median (white line) and 25th and 75th percentiles, and dots show data points for individual RBPs (*n* = 82). The significance of an ANOVA test for differences among families is shown. Right: correlation between this correlation and the percentage of N-glycosylation sequence motifs in viral RBPs (shown in Supplementary Table 1). The Pearson *r* coefficient and *P* value are indicated (two-sided test; *n* = 50 cell lines). Source data

We then explored the effects of 24 cellular proteins known to broadly influence viral entry, including caveolins (CAVs)^49^, clathrins (CLTs)^50^, dynamins (DNMs)^51^, vimentin^52^, tetraspanins^53^, interferon-induced transmembrane proteins (IFITMs)^54^, lymphocyte antigen 6 family member E (LY6E)^55^, nuclear receptor coactivator (NCOA) 7 (ref. ^56^), cholesterol-25-hydroxylase (CH25H)^57^ and centaurin-alpha 2 protein (ADAP2)^58^. Viral entry dependence on each of these factors was explored using Pearson correlation between gene mRNA levels and pseudotype infectivity across cell lines (Fig. 6a).

The strongest statistical associations corresponded to IFITMs, which function as broad-range antiviral proteins. Indeed, we found that 50 of the 82 pseudotypes showed a significantly negative correlation (*P* < 0.01) between infectivity and the basal expression of at least one of these three genes. Interestingly, this pattern was strongly dependent on the viral family (one-way ANOVA, *P* < 0.0001). The most negative associations corresponded to peribunyaviruses and nairoviruses, whereas the infectivity of flavivirus, coronavirus and paramyxovirus pseudotypes was weakly correlated with IFITM levels (Fig. 6b). To verify these differences experimentally, we quantified how IFITM2 overexpression altered the infectivity of three peribunyavirus, three nairovirus, one flavivirus and three paramyxovirus pseudotypes in HEK293T cells. As expected from the above correlation analyses, IFITM2 transfection had a marked inhibitory effect (>75%) on peribunyavirus and nairovirus pseudotype infectivity, whereas the reduction was weaker for the other pseudotypes (Fig. 6b). We also found that RBPs that appeared to be more strongly inhibited by IFITM2 showed a more marked tropism towards neuroectoderm-derived cells (*r* = −0.888; *P* < 0.0001; Fig. 6c). Basal IFITM2 levels were indeed significantly lower in neuroectoderm-derived compared to other cell lines (Wilcoxon test, *P* = 0.00012; Fig. 6c). This suggests that viral RBPs more strongly inhibited by IFITM2 tend to show a tropism more restricted toward cells expressing lower basal levels of this protein, such as neuroectodermal cells.

In contrast to IFITMs, positive correlations with pseudotype infectivity were found for several endocytosis factors known to be exploited by viruses, including CAV1 and CAV2, the clathrin heavy chain (CLTC) and DNM3. However, CLTC dependence was conditional to the predicted N-glycosylation density of the RBPs (*r* = −0.637, *P* < 0.0001; Fig. 6d). It has been shown that glycosylation inhibits the endocytosis of cellular transmembrane proteins^59^, and our data suggest that this may also apply to viral clathrin-mediated viral endocytosis. The association between CLTC expression and pseudotype infectivity also varied significantly across viral families (one-way ANOVA, *P* < 0.0001; Fig. 6d).

Finally, genes encoding other proteins such as LY6E and several tretraspanins showed variable correlations with pseudotype infectivity, which will deserve future investigation. For instance, LY6E has been shown to enhance viral infection, mainly in flaviviruses, but also in some togaviruses, orthomyxoviruses and retroviruses^60^. We found that LY6E mRNA levels correlated positively with pseudotype infectivity in some cases but negatively for most pseudotypes. Our observations are consistent with work suggesting an antiviral role for this gene^55^.

## Discussion

Our results suggest that incompatibilities between viral entry factors across host species may not constitute a major barrier to zoonosis and that entry factors are sufficiently conserved to allow viral transmission. This agrees with work showing that viral RBPs are rarely a major target of natural selection during host jumps^6^. Such entry factors can be proteins but also cell-surface molecules such as carbohydrate moieties. We examined how sialic acid and heparan sulfate proteoglycan depletion impacted pseudotype entry, but more work is needed to understand whether RBPs use other types of carbohydrates or proteinaceous receptors.

Coronaviruses constituted a remarkable exception as most of their RBPs failed to mediate entry into any of the human cell lines tested or showed a narrow cell tropism. Known coronavirus receptors such as ACE2 and DPP4 were expressed in few cell lines, in contrast to ubiquitous viral receptors such as DAG1, LDLR, NPC1 or EFNA1. We found that the requirement of protease-mediated activation only partially explains this narrow tropism, as trypsin treatment failed to unlock human cell infectivity in most cases. Whether another dose of trypsin or other proteases may activate coronavirus spikes more efficiently and allow human infectivity deserves further investigation.

Our results reveal that the availability of a specific receptor is often not a limiting factor for viral entry. Some receptors may be necessary for viral entry but expressed ubiquitously, whereas in other cases, entry may be achieved through multiple alternative receptors and pathways. It remains to be shown whether these findings hold in the cells relevant to the transmission route of each specific virus (for example, lung cells for respiratory viruses). Despite the broad compatibility between viral RBPs and human entry factors shown here, viral entry could still represent a block to zoonosis if the receptor is poorly expressed on cells present at the site of viral exposure. For instance, a recent study showed that although simian arteriviruses were able to use the human version of their receptor CD163, they were unable to infect CD163-expressing human macrophages, which could be due to suboptimal entry or to post-entry blocks^61^.

We also show how broadly acting host factors play a role in determining viral tropism. Notably, IFITM expression showed a negative correlation with RBP-mediated entry, particularly for peribunyaviruses and nairoviruses. Although IFITMs are well-characterized broad-spectrum antiviral factors, how their effect varies across viral families remains poorly understood. RBPs may show different levels of susceptibility to IFITM-mediated restriction, or, alternatively, the strength of this effect relative to those of other entry factors may vary across RBPs. We also found viral family-dependent effects for other broad-range entry factors such as CLTC and identified a link between RBP glycosylation and clathrin-mediated viral endocytosis that deserves future investigation.

Many researchers attempt to predict which viral species are more likely to emerge in humans^62^, and several studies have identified viral features associated with cross-species transmission and zoonosis^7–10^. For instance, it has been shown that machine learning applied to viral genomic data can predict whether a virus is vector borne^63^ or at risk of infecting humans^64^. However, because functional information on wildlife viruses is often lacking, data on their ability to infect human cells are rarely included in such predictions. Our study contributes to filling this gap by extensively characterizing the ability of RBPs from animal viruses to mediate entry into human cells and showing that this ability can be predicted to some extent.

However, one limitation of our study is that we mainly used standard cell lines. Performing such a systematic screening in human primary cells or organoids would be technically much more complex. We verified that the expression levels of cell-surface genes in NCI-60 cells correlated well with those reported for normal tissues. Moreover, many viral pseudotypes were also infectious in HUVEC primary cells. Tumoural cells are widely used models in virology, including some NCI-60 cells such as A549, and, although these cells show abnormal cell physiology, they allow testing whether animal viruses are compatible with the human version of their native-host entry factors. A major advantage of the NCI-60 panel is that their gene expression profile has been extensively characterized, which allowed us to explore the molecular determinants of viral entry. Thus, the NCI-60 panel offers an experimentally tractable system for high-throughput cell culture screening and infection and a valuable tool for investigating virus–host interactions, as shown previously^65,66^.

Another limitation of our approach is that we have focused on viral pseudotypes. This allows the study of entry mechanisms even for viruses that have never been isolated, as is the case for several viral species included in our analysis. Although it is largely accepted that pseudotypes faithfully mimic the entry process of native viruses, we cannot exclude that some differences between pseudotypes and real viruses (for example, RBP incorporation levels or particle geometry) may affect entry efficiency into human cells. We used two different pseudotyping platforms to mitigate this risk.

Finally, viral entry is only the first step that a virus must complete to productively infect a cell, produce progeny virions and spread at the intra-host and population levels. These other aspects of infection should also be studied to achieve a more comprehensive view of zoonotic risks. Indeed, our results suggest that the post-entry stages of the infection cycle, as well as epidemiological and ecological factors, may be more critical determinants of viral zoonosis than entry.

## Methods

### RBP sequence retrieval and phylogenetic analyses

For each viral family, viral species were retrieved from both the International Committee on Taxonomy of Viruses (https://ictv.global) and the National Center for Biotechnology Information (NCBI) RefSeq (https://www.ncbi.nlm.nih.gov/refseq/) databases. One representative of each species was selected except in some cases (for example, betacoronavirus 1) due to special relevance or different origins. The genomes of the selected virus were retrieved from the NCBI database, and their CDSs (coding DNA sequences) were translated into protein and annotated with InterProScan v5.48-83.0 (https://www.ebi.ac.uk/interpro/) to identify the protein of interest. For flavivirus and nairovirus polyproteins, the region of interest was retrieved directly from NCBI Entrez. Protein sequences were aligned using Clustal Omega (ebi.ac.uk/jdispatcher/msa/clustalo; v1.2.4). The best amino acid substitution model was then selected using ProtTest3 (github.com/ddarriba/prottest3; v3.4.2), and a maximum-likelihood tree was built using RaxML-NG (https://github.com/amkozlov/raxml-ng; v1.0.1).

### Selection and synthesis of RBP sequences

For each viral family, species of interest were selected across different clades to cover as much viral diversity as possible. The full RBP gene sequence was synthesized, with the exception of the *Flaviviridae* and *Togaviridae* families where only parts of the structural polyprotein gene were synthesized (C-terminal part of C-E1E2 and E3E26kE1, respectively). All sequences were codon-optimized for human expression, except for VSV, Ebola and Tamiami virus. RBP-coding DNA was cloned into a pcDNA3.1-C-HisTag vector (Genscript). For paramyxoviruses, fusion proteins were cloned into a pcDNA3.1-C-Flag vector, and attachment glycoproteins were cloned into a pcDNA3.1-N-HisTag vector. For Nipah virus, the F and G proteins were cloned in a single pCI-Neo-G-IRES-F plasmid. The Ebola GP-encoding plasmid was obtained from Addgene. The VSV, Ebola, Tamiami and Nipah expression constructs did not contain any protein tag. Information about RBPs is provided in Supplementary Table 1.

### Host information

Known virus–host associations were retrieved from the Virus-Host Database (genome.jp/virushostdb) and the Virion Database (viralemergence.github.io/virion). Each association was manually verified. Virus isolation, sequencing and PCR evidence were considered, while serologic evidence was not used to avoid false positives due to cross-reactivity. For each association, the original or a relevant publication was retrieved. The curated data and PubMed identifiers for each virus–host association are provided in Supplementary Table 2. Information on virus passaging in cell culture is provided in Supplementary Table 3, which shows the cell lines used and includes a reference for each virus (typically the reference reporting the cell line closest to humans).

### VSV pseudotyping

T75 flasks were coated with poly-d-lysine (Gibco) for 2 h at 37 °C, washed with water and seeded with 8 × 10^6^ HEK293T cells. The following day, cells were transfected with 30 µg of viral glycoprotein expression plasmid using Lipofectamine 2000 (Invitrogen) following the manufacturer’s instructions. For paramyxoviruses, cells were transfected with a mixture of 15 µg of fusion protein- and 15 µg of haemagglutinin/glycoprotein-expressing plasmids. To produce bald pseudotypes to be used as negative controls in infection experiments, cells were transfected with an empty pcDNA3.1 vector. At 24 h post transfection, cells were inoculated at a multiplicity of infection (MOI) of 3 infectious units per cell for 1 h at 37 °C with a VSV encoding GFP, lacking the glycoprotein gene G (VSVΔG-GFP) and previously pseudotyped with G. Cells were washed three times with phosphate-buffered saline (PBS), and 8 ml of Dulbecco's modified essential medium (DMEM) supplemented with 2% fetal bovine serum (FBS) was added. Supernatants containing pseudotypes were collected 24 h later, cleared by centrifugation at 2,000 *g* for 10 min, passed through a 0.45 µm filter, aliquoted and stored at −80 °C.

### Lentivirus pseudotyping

Six-well plates were coated with poly-d-lysine (Gibco) for 2 h at 37 °C, washed with water and seeded with 10^6^ HEK293T cells. The following day, cells were transfected with 0.83 µg of pCMV∆R8.2 packaging plasmid, 0.83 µg of pTRIP-GFP and 0.83 µg of viral RBP expression plasmid using Lipofectamine 3000 (Invitrogen) following the manufacturer’s instructions. For paramyxoviruses, plasmids encoding the fusion protein and the haemagglutinin/glycoprotein were co-transfected with the pCMV∆R8.2 and pTRIP-GFP plasmids at a 1:1:1:1 ratio (2.5 µg of DNA in total). Supernatants containing pseudotypes were collected 48 h later, cleared by centrifugation at 2,000 *g* for 10 min, aliquoted and stored at −80 °C.

### Western blotting

A 1 ml volume of supernatant containing pseudotype was pelleted by centrifugation at 30,000 *g* for 2 h at 4 °C and lysed in 30 µl of NP-40 lysis buffer (Invitrogen) for 30 min on ice. Approximately 10^6^ pseudotype-producing cells were lysed for 30 min on ice in 100 µl of NP-40 lysis buffer. Lysates were cleared by centrifugation at 15,000 *g* for 10 min at 4 °C. Cleared lysates were mixed with 4× Laemlli buffer (Bio-Rad) supplemented with 10% β-mercaptoethanol and denatured at 95 °C for 5 min. Proteins were separated by sodium dodecyl sulfate–polyacrylamide gel electrophoresis on a 4–20% Mini-PROTEAN TGX Gel (Bio-Rad) and transferred onto a 0.45 µm PVDF membrane (Thermo Scientific). Membranes were blocked for 1 h at room temperature in TBS-T (20 mM tris, 150 mM NaCl, 0.1% Tween-20, pH 7.5) supplemented with 3% bovine serum albumin (BSA; Sigma). Membranes were then incubated for 1 h at room temperature with the following primary antibodies: mouse anti-His-Tag (dilution 1:1,000, clone HIS.H8, Invitrogen MA121315), mouse anti-Flag (dilution 1:1,000, clone M2, Sigma-Aldrich F1804), mouse anti-VSV-M (dilution 1:1,000, clone 23H12, Kerafast EB0011) or rabbit anti-GAPDH (dilution 1:3,000, Sigma-Aldrich ABS16). Membranes were washed 3 times with TBS-T and incubated for 1 h at room temperature with an HRP-conjugated anti-mouse (dilution 1:50,000, Invitrogen, G-21040) or anti-rabbit (dilution 1:50,000, Invitrogen, G-21234) secondary antibody. After three washes in TBS-T, the signal was revealed with SuperSignal West Pico PLUS (Thermo Scientific) following the manufacturer’s instructions. Images were acquired on an ImageQuant LAS 500 (GE Healthcare) and analysed with Fiji software (v2.1.0).

### HEK293T and HUVEC cells

HEK293T cells (ATCC) were cultured in DMEM (Gibco) supplemented with 10% FBS (Gibco), 10 units per ml penicillin, 10 µg ml^−1^ streptomycin (Gibco) and 250 ng ml^−1^ amphotericin B (Gibco). HUVECs were kindly provided by I. Fariñas (Universitat de València) and were cultured in Endothelial Cell Growth Medium (PromoCell) supplemented with 18.5% Growth Medium SupplementMix (PromoCell), 10 units per ml penicillin, 10 µg ml^−1^ streptomycin (Gibco) and 250 ng ml^−1^ amphotericin B (Gibco). Cells were regularly shown to be free of mycoplasma contamination by PCR.

### NCI-60 cells

The NCI-60 panel (dtp.cancer.gov/discovery_development/nci-60) was purchased from the National Cancer Institute. The panel consists of 60 well-characterized tumoural cell lines from various origins. Nine cell lines showing poor growth or lack of infection by VSV were excluded (RPMI-8226, SR, HL-60, COLO 205, NCI-H322, HCC2998, HS 578T, LOX IMVI and KM12). Information about the remaining 51 cell lines is provided in Supplementary Table 5. All cells were cultured in RPMI (Roswell Park Memorial Institute) (Gibco) supplemented with 10% FBS (Gibco), 10 units per ml penicillin, 10 µg ml^−1^ streptomycin (Gibco), 250 ng ml^−1^ amphotericin B (Gibco) and 5 µg ml^−1^ prophylactic plasmocin (InvivoGen). For adherent cells, confluence was quantified automatically with the Incucyte SX5 Live-Cell Analysis System (Sartorius) using the Artificial Intelligence Confluence segmentation algorithm with a clean-up hole fill parameter of 100 µm^2^ and filtering out segments smaller than 200 µm^2^. Cells were washed with PBS and detached with trypsin (Gibco); complete culture medium was added, and cells were dispensed in new culture dishes for maintenance and in 96-well plates for infection experiments the following day. For suspension cell lines, cells were counted and diluted to a concentration of 0.5 × 10^6^ cells per ml; for infection, 96-well plates were coated with poly-d-lysin (Gibco) and washed with water, and 60,000 cells were added to each well and let adhere for at least 2 h. All cell lines were regularly shown to be free of mycoplasma contamination by PCR. Most lines (48 of 51) were authenticated by short tandem repeat (STR) genotyping. Briefly, genomic DNA was extracted using the PureLink Genomic DNA Mini Kit (Invitrogen) following the manufacturer’s instructions. Genomic DNA concentration was quantified using a NanoDrop One spectrophotometer (Thermo), and 1 ng of DNA was amplified by PCR using the AmpFℓSTR Identifiler Plus PCR Amplification Kit (Applied Biosystems) following the manufacturer’s instructions. Amplified fragments were analysed by capillary electrophoresis using a 3730 XL DNA Analyzer (Applied Biosystems). Chromatograms were analysed using the Osiris software (https://ncbi.nlm.nih.gov/osiris; v2.16). Results were compared to the STR profiles of the NCI-60 cell lines available online (web.expasy.org/cellosaurus) using a relaxed similarity metric.

### Infection with VSV pseudotypes

Viral suspensions were mixed 1:1 with an anti-VSV-G monoclonal antibody to remove residual VSV-G and incubated for 20 min at 37 °C. This antibody was obtained in-house from a mouse hybridoma cell line. Cell culture medium was removed, and cells were inoculated with 50 µl of the antibody-treated pseudotypes. Plates were incubated for 2 h at 37 °C, and 50 µl of RPMI supplemented with 5% FBS was added to each well. After 18–24 h, cells were imaged in the Incucyte SX5 Live-Cell Analysis System (Sartorius). Cell confluence and the percentage of GFP-positive area were quantified automatically with the Incucyte Analysis software (v2022BRev2). The following internal controls were run in all infection assays and for all cell lines. First, a blank control inoculated with a bald pseudotype was used to measure the background signal resulting from cell auto-fluorescence or residual VSV-G-pseudotyped particles. The values obtained in these negative controls were subtracted from the corresponding measurements. Second, a control in which cells were inoculated with VSVΔG-GFP pseudotyped with its own G protein allowed us to assess inter-assay reproducibility. Each pseudotype was assayed twice in experimental blocks performed on different days.

### Quantitation of VSV pseudotype infectivity

The proportion of infected cells, *Q*, was measured as the ratio between the GFP area and cell confluence, subtracting the value obtained for the corresponding blank control. To correct for saturation effects in highly infected wells, we transformed *Q* values into MOIs as follows: MOI = −ln (1 − *Q*). For *Q* > 0.95, an MOI of 3 infectious units per cell was assumed. To account for differences in pseudotype titre, for each pseudotype and assay, relative MOI values were calculated as *R* = 100 × MOI/max(MOI), that is, as a percentage of the maximal MOI observed among the 51 cell lines. Finally, values were log-transformed as log_2_(*R* + 1). For the VSV-G internal controls, the median Pearson correlation coefficient between log_2_(*R* + 1) values from different experimental blocks was *r* = 0.834, and 91.2% of the individual data points were within twofold of the inter-assay average, validating the reproducibility of the assays. In addition to quantifying infection, for each of the 102 × 51 RBP–cell combinations, we obtained a dichotomous variable indicating the presence or absence of infection. The following conditions had to be met in both replicates of each RBP–cell combination for this variable to be positive: (1) *R* > 1; (2) *Q* value at least 5 times higher than in the corresponding blank control; (3) *Q* > 0.05%. Visual inspection of multiple wells was used to establish these criteria. Average *Q* values, log_2_(*R* + 1) values and positivity data are provided in Supplementary Table 6. These averages were obtained from the two technical replicates for all pseudotypes, except for VSV-G (used as internal control), which was assayed 42 times. For the initial exploratory analysis performed in HEK293T and HUVEC cells, a single replicate was performed, and infection was binarized using the second and third criteria only.

### Infection with lentiviral pseudotypes

HEK293T were seeded in 96-well plates and infected the following day with 100 μl of lentiviral pseudotype suspension. Plates were imaged 2 days later using the Incucyte SX5 Live-Cell Analysis System (Sartorius). Successful infections were determined visually. To avoid false-negative results, pseudotypes showing a negative result using this initial approach were also spinoculated onto HEK293T at 1,200 *g* for 1 h at 4 °C. After 2 days at 37 °C, the infection outcome was similarly evaluated using the Incucyte SX5 Live-Cell Analysis System (Sartorius).

### Human gene cloning

For each gene of interest, the sequence of the main human transcript (flagged as MANE Select) was retrieved from Ensembl (https://www.ensembl.org) and the NCBI RefSeq databases (http://ncbi.nlm.nih.gov/refseq). RNA was extracted from HEK293T cells or NCI-60 cell lines expressing the gene of interest according to RNA-seq data, using RNAzol (Sigma-Aldrich) and following the manufacturer’s instructions. RNA was reverse-transcribed into complementary DNA using Oligo dT and SuperScript IV Reverse Transcriptase (Invitrogen) following the manufacturer’s instructions. The cDNAs were used as templates for PCR amplification using primers detailed in Supplementary Table 7. PCR-amplified transcripts were cloned into a pcDNA3.1-C-Flag or pcDNA3.1-N-HA vector with restriction enzymes or through HiFi assembly. For restriction enzyme cloning, restriction sites were added in amplification primers. The vector and PCR products were digested with restriction enzymes and band-purified (vector) or cleaned (PCR products) using the Zymoclean Gel DNA Recovery Kit (Zymo Research) or the DNA Clean & Concentrator-5 kit (Zymo Research), respectively. Purified PCR products and the vector were mixed at a 1:3 molar ratio and ligated using T4 DNA ligase (Thermo Scientific). For HiFi cloning, the pcDNA3.1-C-Flag vector was linearized by PCR (forward primer, 5′-GATTACAAGGATGACGACGATAAGTG-3′; reverse primer, 5′-GGTGGCAAGCTTAAGTTTAAACGCTAG-3′). Amplification primers contained a 20-nucleotide tail overlapping with the 5′ or 3′ ends of the linearized pcDNA3.1-C-Flag vector. The linearized vector and PCR-amplified sequences were mixed at a 1:2 molar ratio and assembled using the NEBuilder HiFi DNA Assembly Master Mix (New England Biolabs) following the manufacturer’s instructions. Phusion Hot Start II High-Fidelity DNA polymerase (Thermo Scientific) was used for all PCR steps. Assembled products were transformed into NY5α competent cells (NZYtech). Correct insertion was checked by colony PCR using vector-specific primers (forward, 5′-GAGAACCCACTGCTTACTGGC-3′; reverse, 5′-AGGGTCAAGGAAGGCACG-3′) and the NZYTaq II 2× Green Master Mix (NZYtech). Plasmids with correct insertions were checked by Sanger (Eurofins) or whole-plasmid high-throughput sequencing (Plasmidsaurus). In addition, correct production of the protein of interest was checked by western blot of HEK293T-transfected cells using an anti-Flag antibody (Sigma-Aldrich).

### Host gene overexpression

For each pseudotype–gene combination, the most appropriate cell line was based on the following criteria: low expression of the gene to be tested, high transfection efficiency, low infection by the virus to be tested and high susceptibility to VSV. Cells were plated in 96-well plates. The following day, cells were transfected with the gene-encoding plasmid or an empty vector control using Lipofectamine 2000 or Lipofectamine 3000 (Invitrogen), following the manufacturer’s instructions. A control for transfection efficiency was included (GFP expression plasmid). After 20–24 h, pseudotypes were mixed 1:1 with an anti-VSV-G monoclonal antibody and incubated for 30 min at 37 °C. Cell culture medium was removed, and cells were inoculated with 50 µl of antibody-treated pseudotypes. Plates were incubated for 2 h at 37 °C, and 50 µl of RPMI supplemented with 5% FBS was added to each well. After 18–24 h, cells were imaged in the Incucyte SX5 Live-Cell Analysis System (Sartorius). Cell confluence and the percentage of GFP-positive area were quantified automatically with the Incucyte Analysis software. The proportion of infected cells was calculated as the ratio between the GFP area and cell confluence. To verify expression of the transfected genes, in parallel experiments, cells were fixed with 4% paraformaldehyde for 10 min at room temperature. Cells were then washed with PBS and permeabilized with PBS 0.5% Triton X-100 for 20 min at room temperature. Cells were incubated with blocking buffer (PBS, 1% BSA, 0.1% Tween 20) for 30 min at room temperature. Cells were then incubated 1 h at room temperature with a primary anti-Flag antibody (Sigma-Aldrich, F1804, diluted 1:500 in blocking buffer), washed three times with PBS and incubated 1 h at room temperature with an AF-488-conjugated secondary anti-mouse antibody (Invitrogen, A32766, diluted 1:500 in blocking buffer). After three washes with PBS, images were acquired on the Incucyte SX5 Live-Cell Analysis System (Sartorius) at ×20 magnification.

### Effect of trypsin on coronavirus pseudotype infectivity

The protocol was adapted from a previous study^15^. Coronavirus pseudotypes were incubated with N-tosyl-L-phenylalanine chloromethyl ketone (TPCK)-treated trypsin (Sigma-Aldrich) at a 2.5 mg ml^−1^ concentration for 15 min at 37 °C or mock-treated with DMEM only. Trypsin-treated pseudotypes were then mixed 1:1 with cold anti-VSV-G antibody and incubated for 15 min at 4 °C. An aliquot was frozen for future western blot analysis. To remove the excess of trypsin, pseudotypes were centrifuged 2 h at 4 °C at 30,000 *g* and resuspended in RPMI 5% FBS. NCI-60 cell culture medium was removed, and cells were inoculated with 90 µl of treated pseudotype before spinoculation at 1,200 *g* for 1 h at 4 °C. Cells were then cultured at 37 °C for 24 h before acquiring images on the Incucyte SX5 Live-Cell Analysis System (Sartorius). Quantitation of pseudotype infectivity was performed as indicated above.

### Effect of sialic acids on pseudotype entry

SNB-19 cells were plated in a 96-well plate in the presence or absence of 40 µg ml^−1^ of neuraminidase from *Clostridium perfringens* (Sigma-Aldrich). The following day, cells were inoculated with all viral pseudotypes previously shown to infect SNB-19 cells, in the presence or absence of 40 µg ml^−1^ neuraminidase. Cells were also inoculated with a GFP-expressing influenza A virus (strain PR8) as a positive control for sialic acid depletion. After 18–24 h, cells were imaged in the Incucyte SX5 Live-Cell Analysis System (Sartorius). The infected cell percentage was calculated as the ratio between GFP-positive area and cell confluence.

### Effect of heparan sulfate proteoglycans on pseudotype entry

SNB-19 cells were plated in a 96-well plate and treated the following day with Heparinase III from *Flavobacterium heparinum* (Amsbio, 5 mIU ml^−1^, diluted in DMEM 0.2% BSA) for 1 h at 37 °C. Mock-treated cells were incubated for 1 h at 37 °C with DMEM 0.2% BSA alone. Cells were then washed once with DMEM 0.2% BSA and inoculated with all viral pseudotypes previously shown to infect SNB-19 cells. After 18–24 h, cells were imaged in the Incucyte SX5 Live-Cell Analysis System (Sartorius). The infected cell percentage was calculated as the ratio between GFP-positive area and cell confluence. To verify digestion of heparan sulfate proteoglycans, cells treated with Heparinase III and mock-treated cells were incubated 30 min at room temperature with an antibody specifically recognizing digested heparan sulfates (Amsbio, F69-3G10, diluted 1:100 in DMEM 0.2% BSA), washed once with DMEM 0.2% BSA and incubated 30 min at room temperature with an AF-488-conjugated secondary anti-mouse antibody (Invitrogen, A32766, diluted 1:400 in DMEM 0.2% BSA). After one wash with DMEM 0.2% BSA, images were acquired on the Incucyte SX5 Live-Cell Analysis System (Sartorius) at ×20 magnification.

### Gradient boosting

An XGBoost classification model was used to predict the presence or absence of pseudotype infection signal in NCI-60 cells. A total of 81 features related to the viral family (Toga, Matona, Flavi, Corona, Orthomyxo, Arena, Hanta, Nairo, Peribunya, Phenui, Borna, Filo, Paramyxo, Rhabdoviridae), viral genus (Alpha-, Rubi-, Hepaci-, Pesti-, Alphacorona-, Betacorona-, Gammainfluenza-, Quaranja-, Thogoto-, Mammarena-, Orthohanta-, Mobat-, Thottim-, Orthonairo-, Orthobunya-, Phlebo-, Banda-, Orthoborna-, Orthoebola-, Cueva-, Dianlo-, Morbilli-, Henipa-, Respiro-, Jeilong-, Lyssa-, Sawgrha-, Tupa-, Sunrha-, Merha-, Ledante-, Sripu-, Sprivi-, Arurha-, Ephemero-, Curio-, Tibro-, Hapa-, Vesiculovirus), cell origin (breast, CNS, colon, kidney, lung, melanocytes, ovary, prostate), RBP fusion class (I, II, III) and known hosts in nature (humans, non-human primates, Artiodactyla, Rodentia, Chiroptera, other mammals, birds, fish, arthropods), as well as in cell culture (human, non-human primate, other mammals, fish, birds, invertebrate cells), RBP size and the level of N- and O-glycosylation were used as predictors. XGBoost was implemented in R using the xgboost package (v1.7.8.1)^67^. To mitigate the risks of overfitting, a sixfold cross-validation structured by virus was used to ascertain that highly similar instances associated with a particular virus were not present in both the training and test subsets. This involved splitting the dataset into six disjoint test sets, each containing data from 17 randomly chosen viruses. The area under the ROC curve (AUC) was selected as the evaluation metric. Model complexity was determined using a maximum of 10,000 boosting iterations with 50 rounds as an early stopping criterion, and an optimal set of hyper-parameters was identified using Bayesian optimization with the R package ParBayesianOptimization (CRAN.R-project.org/package=ParBayesianOptimization; v1.2.6). This Bayesian optimization was initialized with 100 random hyper-parameter combinations, followed by a total of 50 refinement epochs, each incorporating 10 new hyper-parameter sets. For every hyper-parameter configuration, the model was evaluated using 10 predefined, randomly generated sixfold cross-validation sets, and the optimization was aimed to maximize the AUC averaged over these sets. The hyper-parameters optimized comprised the maximum depth of boosted trees (max_depth), the fraction of training samples used to construct each tree (subsample), the fraction of predictors used to construct each tree (colsample_bytree), the learning rate (eta), the L1 regularization term (alpha), the L2 regularization term (lambda) and the Lagrangian control for tree split (gamma). Moreover, despite our data being globally well balanced (2,704 positive and 2,498 negative instances), the positive class was weighted based on the ratio of negative to positive instances. To account for model variability in training and robustness across different data splits, the 50 top-ranked models based on their average AUC underwent 25 additional runs, each using a predefined, randomly generated sixfold cross-validation set. The complexity and hyper-parameters of the model showing the significantly highest AUC, determined via a one-tailed one-sample *t*-test, were chosen to train a final model using the entire dataset. To understand how features contributed to the final model predictions, SHAP explanations were obtained using the shapforxgboost R package (cran.r-project.org/web/packages/SHAPforxgboost; v0.1.3). SHAP values dissect each prediction into a unified bias term related to the average model prediction, along with positive and negative additive terms that describe the marginal impact of each feature on the model output^68^.

### Hierarchical cluster analysis

A hierarchical cluster analysis was carried out to classify pseudotypes according to their similarly in infectivity profiles, measured as log_2_(*R* + 1) across the 51 cell lines. Several distance metrics (Pearson correlation distance, cosine distance and Euclidean distance) and linkage methods (unweighted or weighted pair group method with arithmetic mean (UPGMA and WPGMA)), Ward and Neighbor Joining) were tested. The dendrogram obtained by Pearson correlation distance (1 − *ρ*) and WPGMA linkage was the one that best recapitulated the viral phylogeny based on the average size and number of viruses included in groups monophyletic for the viral family. The stability of dendrogram nodes was evaluated applying multiscale bootstrap resampling upon infectivity data using pvclust R package (https://github.com/shimo-lab/pvclust; v2.2.0).

### RNA-seq and proteomics data

A processed RNA-seq dataset was downloaded from the CellMiner website (discover.nci.nih.gov/cellminer/loadDownload.do, RNA-seq—composite expression file), as well as proteomic data available for a subset of virus receptors (SWATH Mass spectrometry—Protein file). An additional proteomics dataset was obtained from https://www.ebi.ac.uk/pride/archive/projects/PXD005940. These omics data were available for all the cell lines except MDA-MB-468. Gene expression values were available as log_2_(reads per kilobase per million (rpkm) + 1). For the genes analysed in this study, RNA-seq data are available in Supplementary Table 8, and proteomics datasets are available in Supplementary Tables 9 and 10. RNA-seq data averaged over 40 human tissues were downloaded from the Human Protein Atlas (https://www.proteinatlas.org/about/download, rna_tissue_hpa file).

### Statistical associations with gene expression data

Multiple linear regression was used to estimate the relative contribution of different virus receptors to observed infectivity data, where the dependent variable was log_2_(*R* + 1) across all cell lines and the independent variables were the standardized log_2_(rpkm + 1) data for receptor genes. In gene overexpression experiments, mean GFP signals were compared using *t*-tests with log-transformed data. The association of infectivity profiles with the expression of broad-range entry factors was evaluated using Pearson correlations between log_2_(*R* + 1) and log_2_(rpkm + 1) data across all cell lines. All statistical tests were two-sided. These statistical analyses were carried out with R and SPSS v28.

### Prediction of N- and O-glycosylation levels

N-Glycosylation and O-glycosylation levels were predicted using deep learning, language model-based tools, namely, LMNglyPred^69^ and LM-OGlcNAc-Site^70^. The estimated fraction of glycosylated residues is provided in Supplementary Table 1. These models are accessible at https://github.com/KCLabMTU.

### Reporting summary

Further information on research design is available in the Nature Portfolio Reporting Summary linked to this article.

## Supplementary information

Supplementary Information Supplementary Figs. 1–24, legends of Tables 1–6 and Uncropped western blots related to Figs. 1–14.

Reporting Summary

Supplementary Tables Supplementary Tables 1–6.

## Source data

Source Data Fig. 1 Statistical source data.

Source Data Fig. 2 Statistical source data.

Source Data Fig. 3 Statistical source data.

Source Data Fig. 5 Statistical source data.

Source Data Fig. 6 Statistical source data.

Source Data Extended Data Fig. 1 Statistical source data.

Source Data Extended Data Fig. 2 Statistical source data.

Source Data Extended Data Fig. 3 Statistical source data.

Source Data Extended Data Fig. 4 Statistical source data.

Source Data Extended Data Fig. 5 Unprocessed western blots.

Source Data Extended Data Fig. 5 Statistical source data.

Source Data Extended Data Fig. 6 Statistical source data.

## Data Availability

Features of the RBPs analysed in this study are available in Supplementary Table 1. Information about natural hosts and cell culture passaging of viruses are available in Supplementary Tables 2 and 3, respectively. The infectivity of VSV pseudotypes in HEK293T and HUVEC cells and of lentiviral pseudotypes in HEK293T cells is provided in Supplementary Table 4. Features of the 51 cell lines of the NCI-60 panel are available in Supplementary Table 5 and at https://dtp.cancer.gov/discovery_development/nci-60/cell_list.htm. VSV pseudotype infection data in these 51 cell lines are shown in Supplementary Table 6. Information about receptor cloning is available in Supplementary Table 7. NCI-60 omics data are available at https://discover.nci.nih.gov/cellminer/loadDownload.do, at https://www.ebi.ac.uk/pride/archive/projects/PXD005940 and in Supplementary Tables 8–10. RNA-seq data from the Human Protein Atlas are available at https://proteinatlas.org/about/download. Known virus–host associations were retrieved from the Virus-Host Database (https://genome.jp/virushostdb) and the Virion Database (https://viralemergence.github.io/virion). Source data are provided with this paper.
