# Supplementary material for: Receptor-binding proteins from animal viruses are broadly compatible with human cell entry factors
Source: Nat Microbiol. 2025 Jan 2;10(2):405–19. doi: 10.1038/s41564-024-01879-4 (PMC11790484; doi:10.1038/s41564-024-01879-4)

nature microbiology

Supplementary information https://doi.org/10.1038/s41564-024-01879-4

Receptor-binding proteins from animal viruses are broadly compatible with human cell entry factors

In the format provided by the authors and unedited

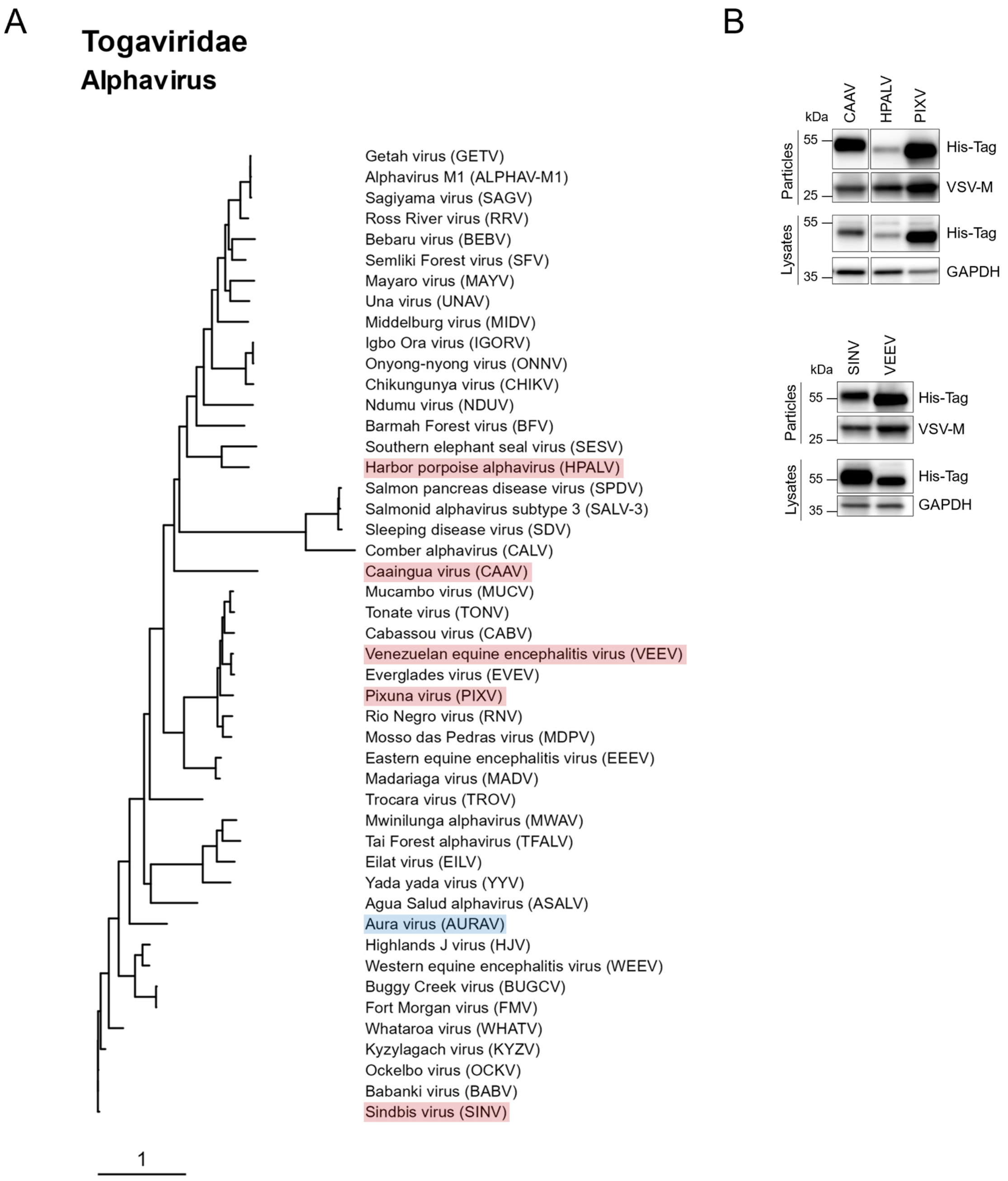

Figure S1. ***Togaviridae*** **structural protein phylogeny for the alphavirus genus and Western Blot confirmation of correct pseudotype production. A.** Maximum-likelihood phylogenetic tree of the CPE3E26kE1 polyprotein. Species included in our analysis are shaded in red. Species shaded in blue are viruses for which pseudotype production was attempted but unsuccessful. **B.** The E3, E26k and E1 proteins were expressed. A His-Tag added to the E1 protein C-terminus was used for detection in producer cells and to show incorporation into VSV pseudotypes. GAPDH and VSV-M were used as loading controls for producer cells and viral pseudotypes, respectively. Western blot analysis was performed once.

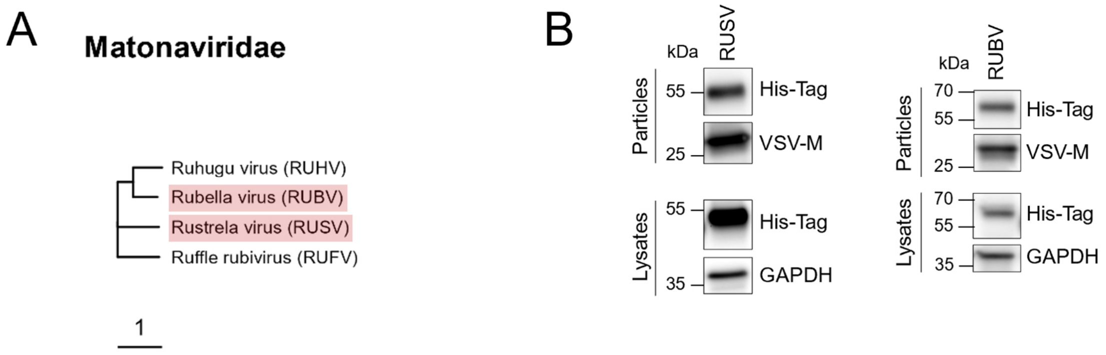

Figure S2. *Matonaviridae* structural protein phylogeny and Western Blot confirmation of correct pseudotype production. A. Maximum-likelihood phylogenetic tree of the CPE2E1 polyprotein. Species included in our analysis are shaded in red. **B.** The C, E2 and E1 proteins were expressed. A His-Tag added to the E1 C-terminus was used for detection in producer cells and to show incorporation into VSV pseudotypes. GAPDH and VSV-M were used as loading controls for producer cells and viral pseudotypes, respectively. Western blot analysis was performed once.

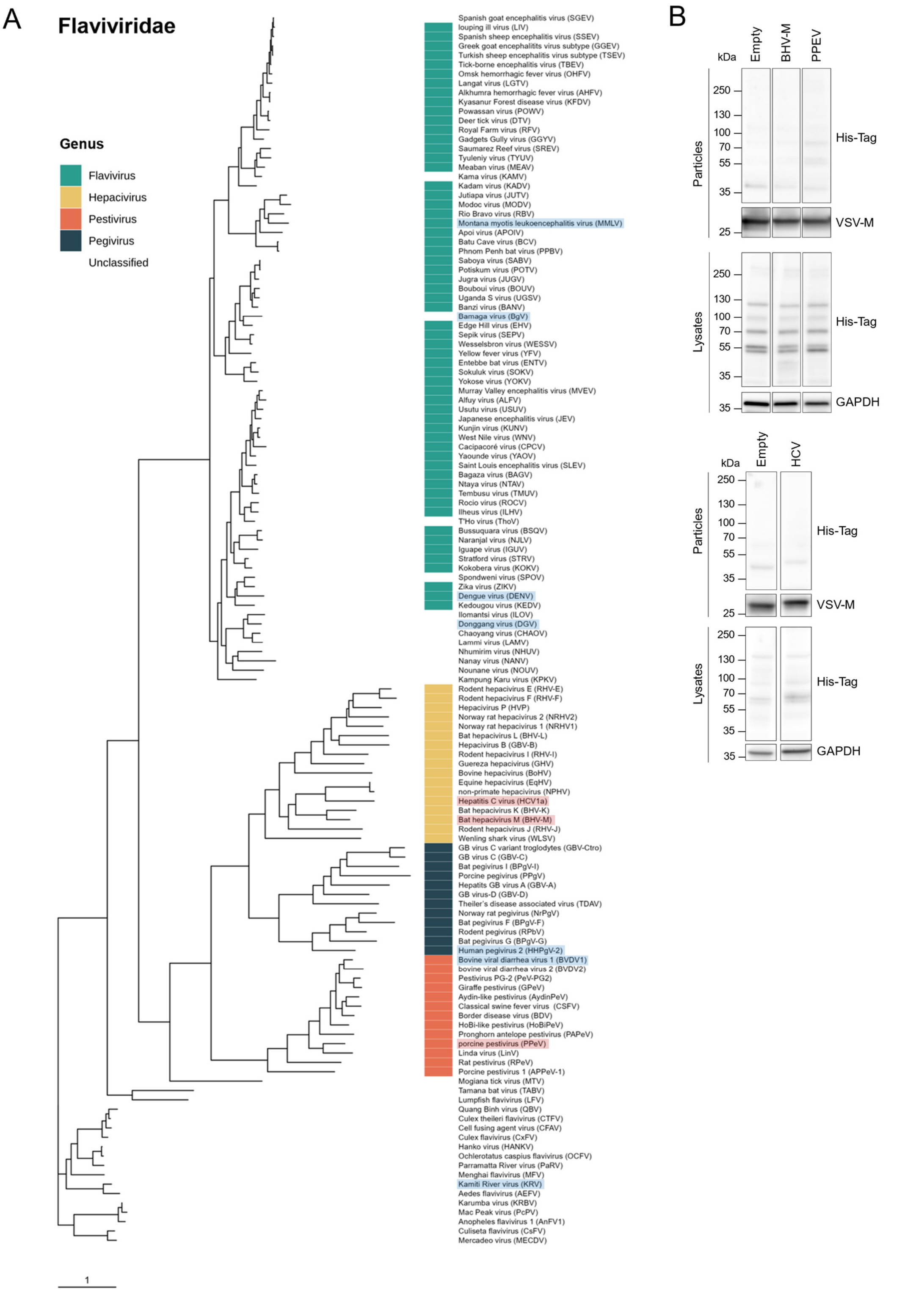

Figure S3. *Flaviviridae* envelope proteins phylogeny and Western Blot confirmation of correct pseudotype production. A. Maximum-likelihood phylogenetic tree. Species included in our analysis are shaded in red. Species shaded in blue are viruses for which pseudotype production was attempted but unsuccessful. **B.** The C terminus of the C protein and the entire E1E2 (HCV and BHV-M) and E^{rns}E1E2 (PPEV) proteins were expressed. A His-Tag added to the E2 protein C-terminus was used for detection in producer cells and to show incorporation into VSV pseudotypes. GAPDH and VSV-M were used as loading controls for producer cells and viral pseudotypes, respectively. Although RBP incorporation could not be observed for BHV-M, PPEV and HCV, infectivity of produced pseudotypes confirmed proper production. Western blot analysis was performed once.

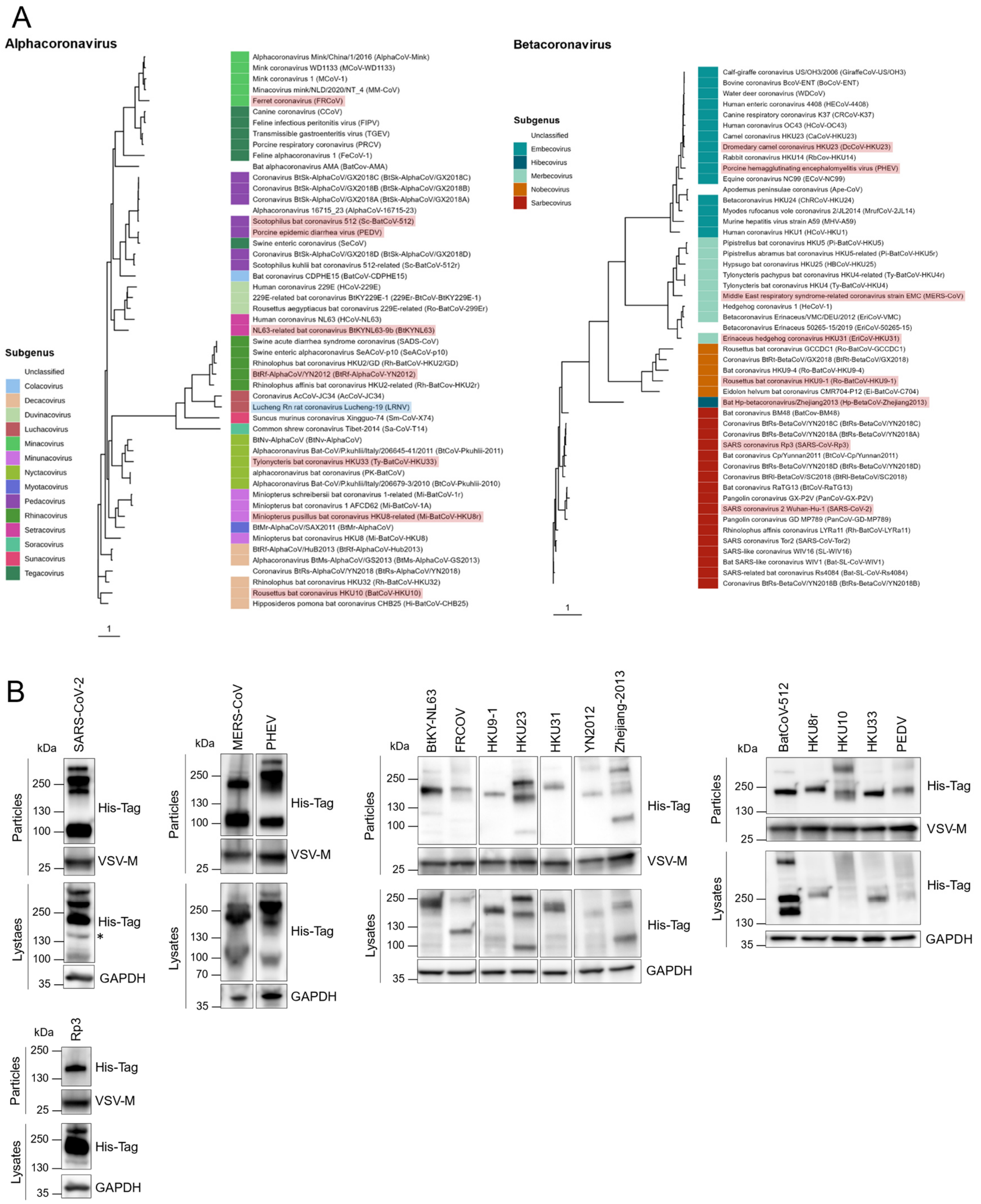

Figure S4. *Coronaviridae* spike protein phylogeny and Western Blot confirmation of correct pseudotype production. A. Separate maximum-likelihood phylogenetic trees for the alpha and betacoronaviruses. Species included in our analysis are shaded in red. Species shaded in blue are viruses for which pseudotype production was attempted but unsuccessful. **B.** A His-Tag added to the spike protein C-terminus was used for detection in producer cells and to show incorporation into VSV pseudotypes. GAPDH and VSV-M were used as loading controls for producer cells and viral pseudotypes, respectively. The asterisk indicates an unspecific band. Western blot analysis was performed once.

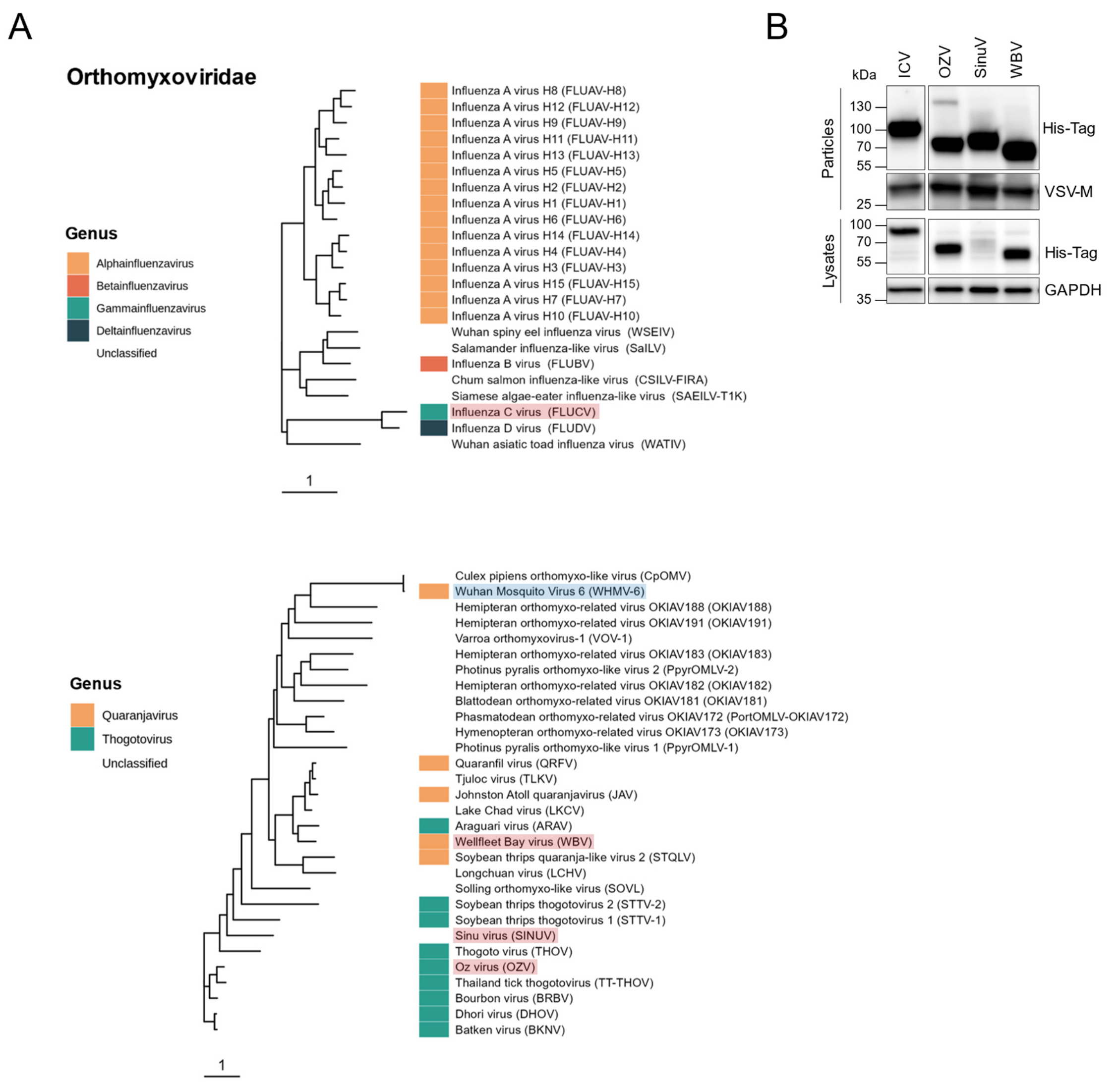

Figure S5. *Orthomyxoviridae* RBP phylogeny and Western Blot confirmation of correct pseudotype production. A. Maximum-likelihood phylogenetic tree of the HA (top tree) or GP (bottom tree) depending on genus. Species included in our analysis are shaded in red. Species shaded in blue are viruses for which pseudotype production was attempted but unsuccessful. **B.** A His-Tag added to the HA or GP C-terminus was used for detection in producer cells and to show incorporation into VSV pseudotypes. GAPDH and VSV-M were used as loading controls for producer cells and viral pseudotypes, respectively. Western blot analysis was performed once.

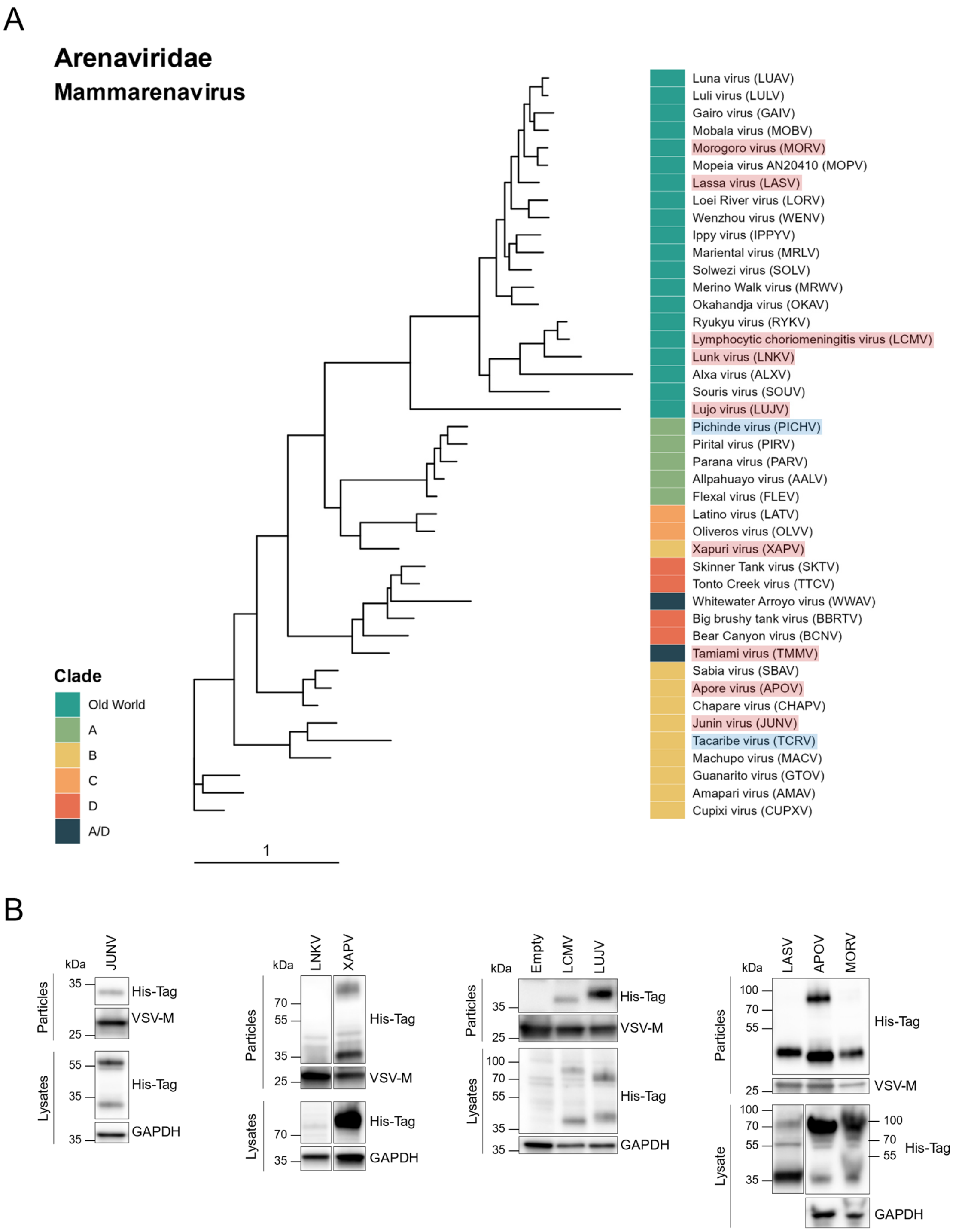

Figure S6. ***Arenaviridae*** **GP phylogeny for the mammarenavirus genus and Western Blot confirmation of correct pseudotype production. A.** Maximum-likelihood phylogenetic tree of the RBPs. Species included in our analysis are shaded in red. Species shaded in blue are viruses for which pseudotype production was attempted but unsuccessful. **B.** A His-Tag added to the GP C-terminus was used for detection in producer cells and to show incorporation into VSV pseudotypes, except for Tamiami virus, for which incorporation was shown by assaying pseudotype infectivity. GAPDH and VSV-M were used as loading controls for producer cells and viral pseudotypes, respectively. Although RBP incorporation could not be observed for LNKV, infectivity of produced pseudotypes confirmed proper production. Western blot analysis was performed once.

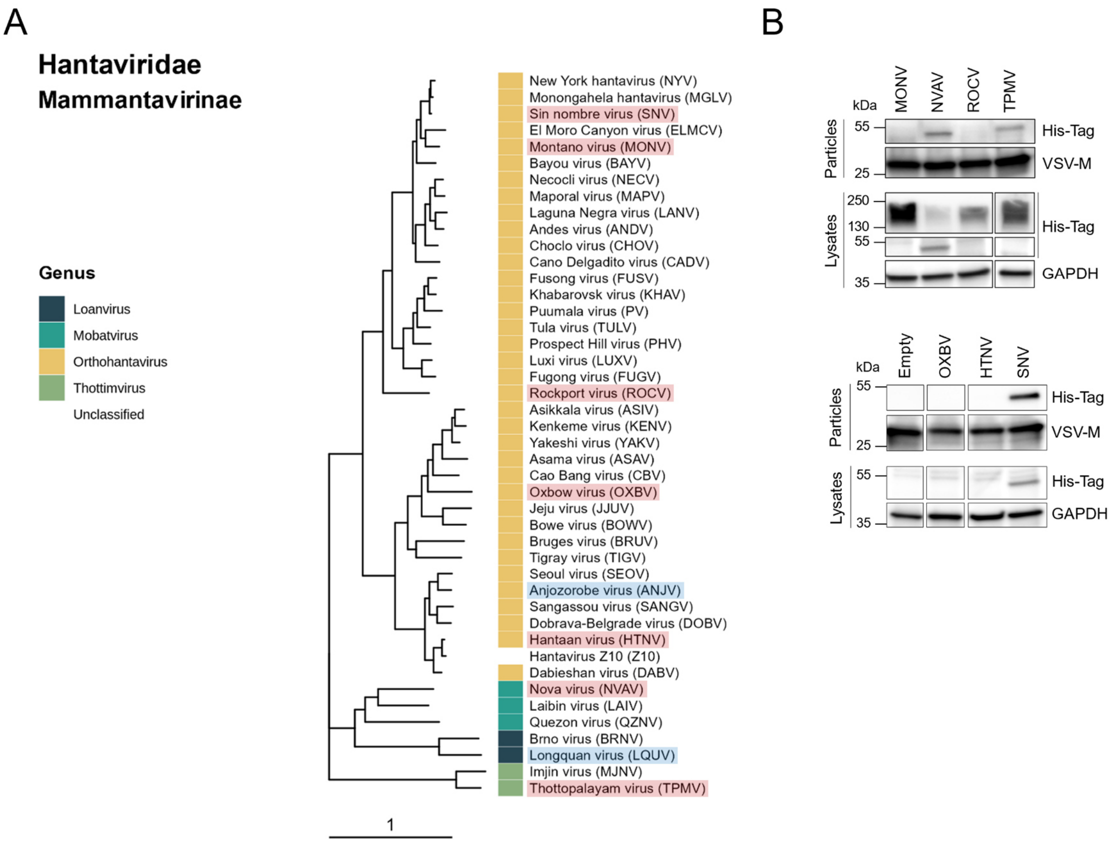

Figure S7. *Mammahantavirinae* subfamily Gc-Gn (GPC) phylogeny and Western Blot confirmation of correct pseudotype production. A. Maximum-likelihood phylogenetic tree, which includes the polyprotein M segment. Species included in our analysis are shaded in red. Species shaded in blue are viruses for which pseudotype production was attempted but unsuccessful. **B.** A His-Tag added to the GPC C-terminus was used for detection in producer cells and to show incorporation into VSV pseudotypes. GAPDH and VSV-M were used as loading controls for producer cells and viral pseudotypes, respectively. Although RBP incorporation could not be observed for MONV, ROCV, OXBV and HTNV viruses, infectivity of produced pseudotypes confirmed proper production. Western blot analysis was performed once.

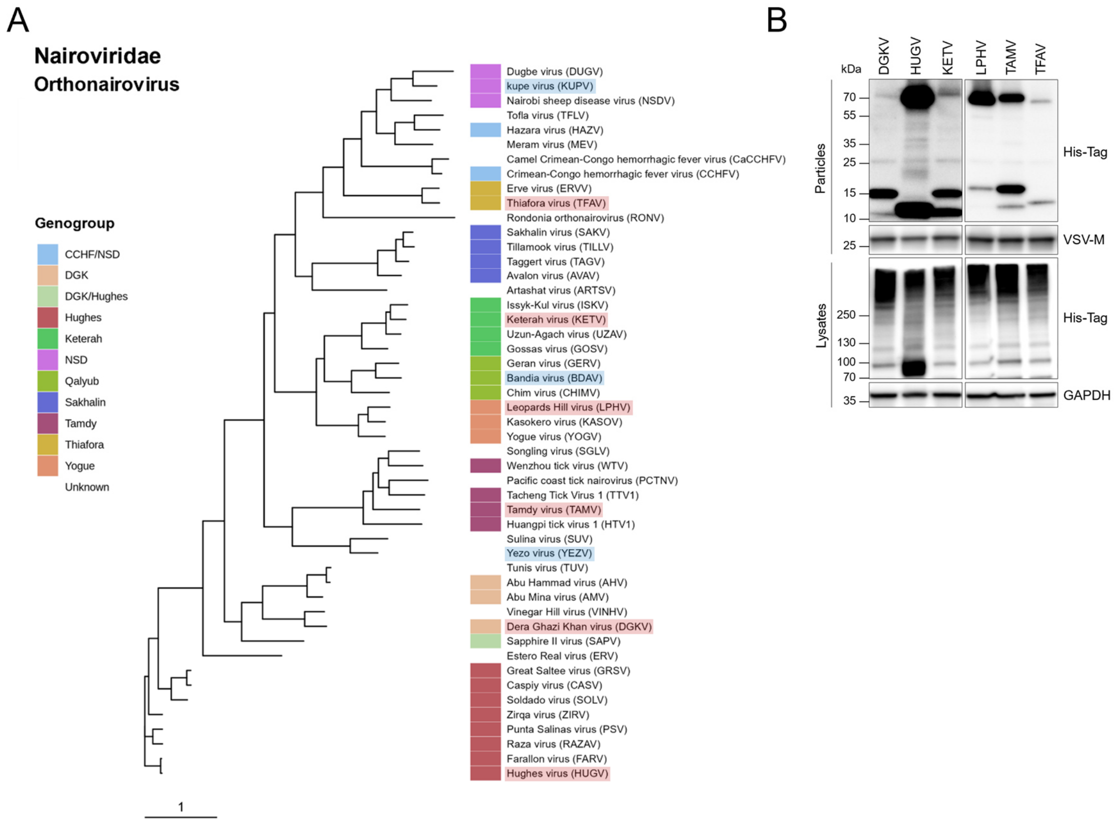

Figure S8. *Nairoviridae* Gc-Gn (GPC) phylogeny for the Orthonairovirus genus and Western Blot confirmation of correct pseudotype production. A. Maximum-likelihood phylogenetic tree, which includes the polyprotein M segment. Species included in our analysis are shaded in red. Species shaded in blue are viruses for which pseudotype production was attempted but unsuccessful. **B.** A His-Tag added to the GPC C-terminus was used for detection in producer cells and to show incorporation into VSV pseudotypes. GAPDH and VSV-M were used as loading controls for producer cells and viral pseudotypes, respectively. Western blot analysis was performed once.

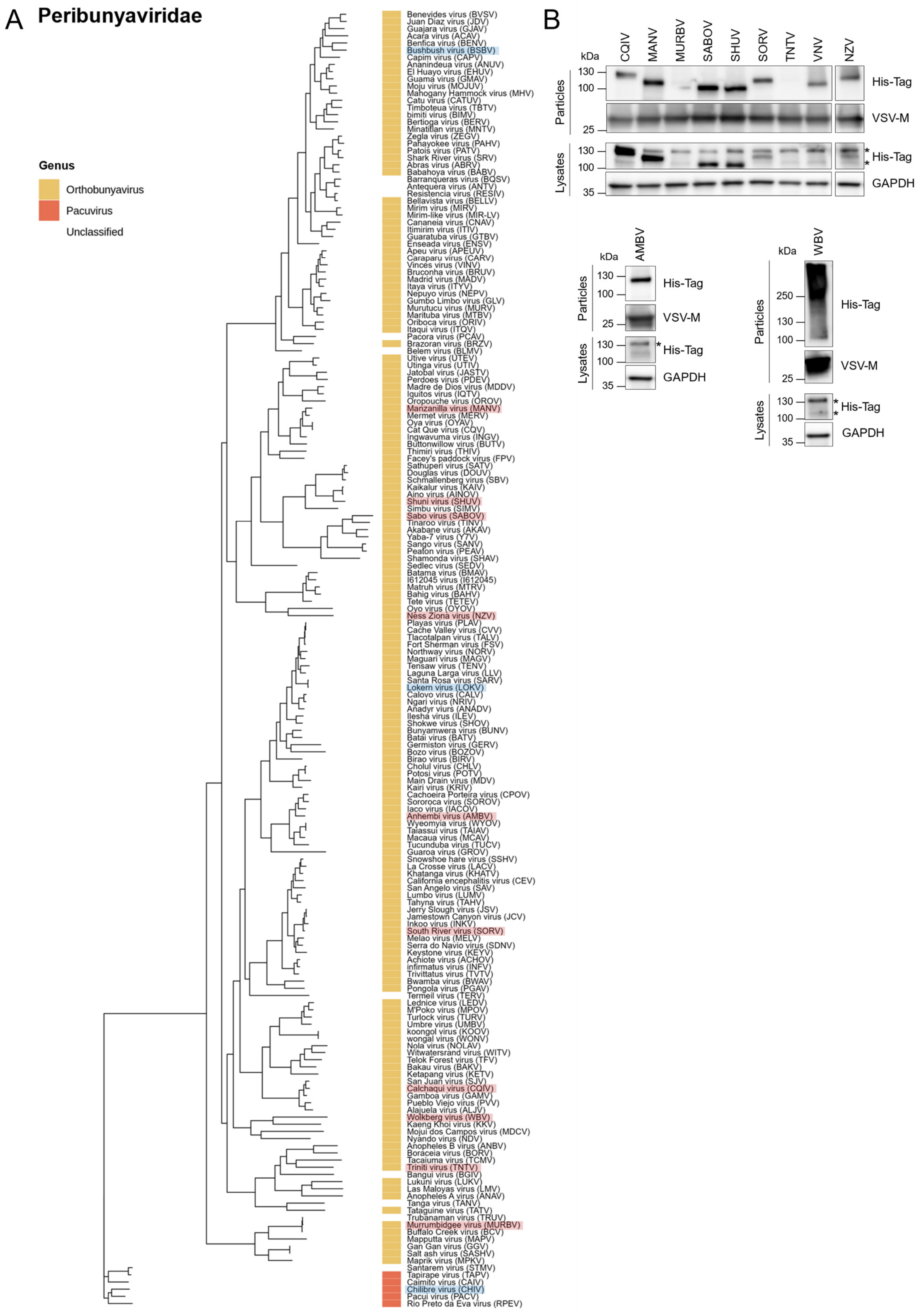

Figure S9. ***Peribunyaviridae*** **Gc-Gn (GPC) phylogeny and Western Blot confirmation of correct pseudotype production. A.** Maximum-likelihood phylogenetic tree, which includes the polyprotein M segment. Species included in our analysis are shaded in red. Species shaded in blue are viruses for which pseudotype production was attempted but unsuccessful. **B.** A His-Tag added to the GPC C-terminus was used to show incorporation into VSV pseudotypes. VSV-M was used as a loading control. Although RBP incorporation could not be clearly observed for TNTV and MURBV, infectivity of produced pseudotypes confirmed proper production. The asterisks indicate unspecific bands. Western blot analysis was performed once.

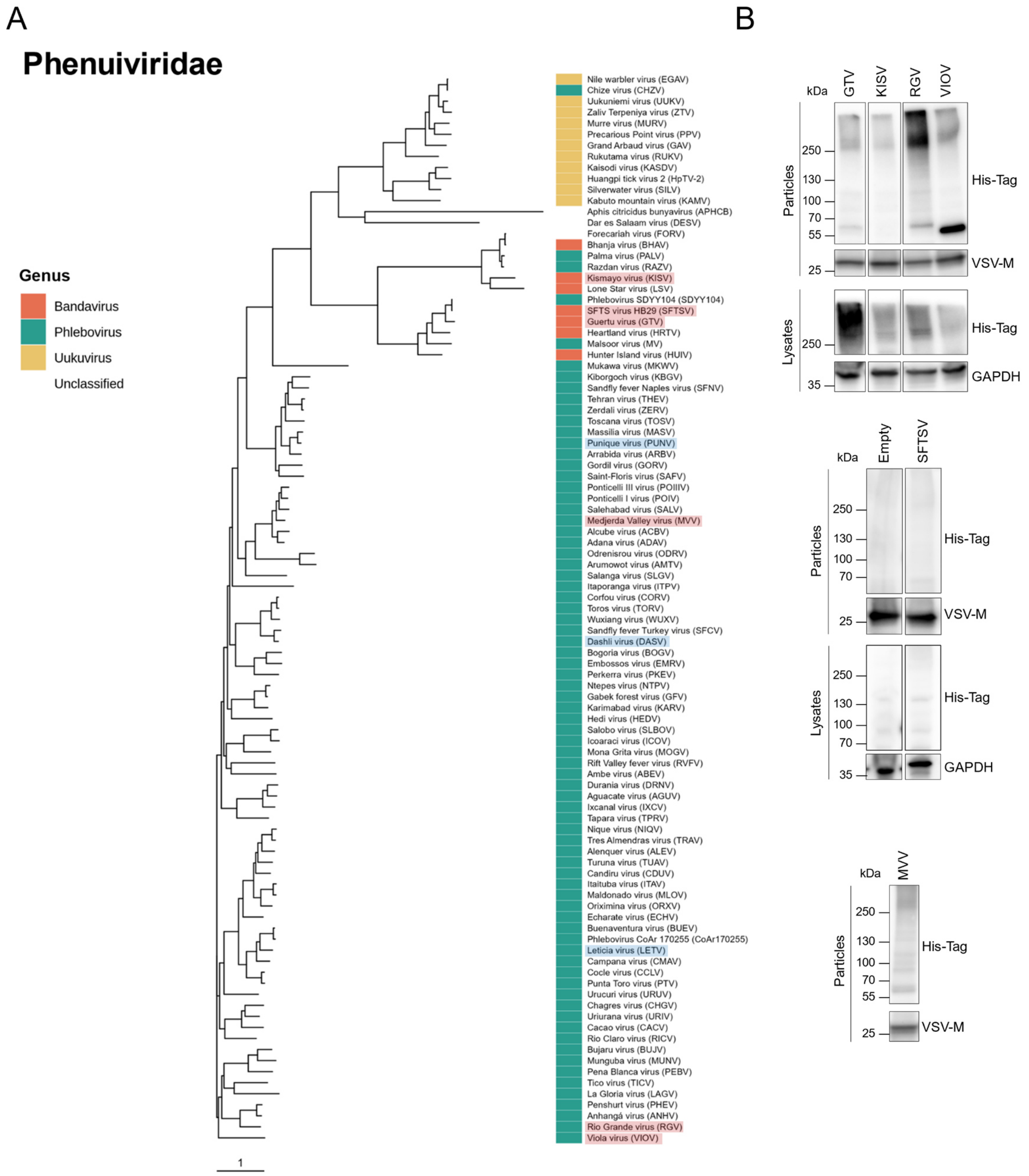

Figure S10. *Phenuiviridae* Gc-Gn (GPC) phylogeny and Western Blot confirmation of correct pseudotype production. A. Maximum-likelihood phylogenetic tree, which includes the polyprotein M segment. Species included in our analysis are shaded in red. Species shaded in blue are viruses for which pseudotype production was attempted but unsuccessful. **B.** A His-Tag added to the GPC C-terminus was used for detection in producer cells and to show incorporation into VSV pseudotypes. GAPDH and VSV-M were used as loading controls for producer cells and viral pseudotypes, respectively. The cell lysate blot for MVV is not shown as no band could be detected. Although RBP incorporation was weakly detected for SFTSV, infectivity of produced pseudotypes confirmed proper production. Western blot analysis was performed once.

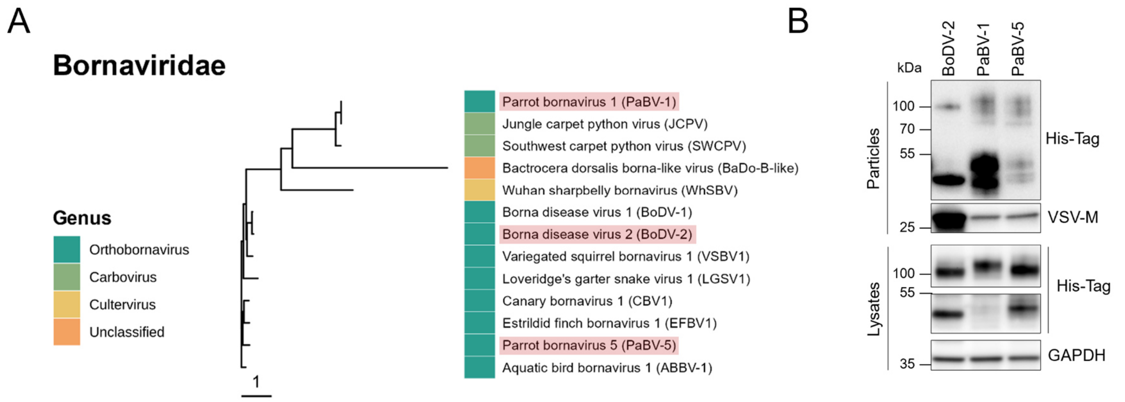

Figure S11. *Bornaviridae* G protein phylogeny and Western Blot confirmation of correct pseudotype production. A. Maximum-likelihood phylogenetic tree. Species included in our analysis are shaded in red. **B.** A His-Tag added to the G protein C-terminus was used for detection in producer cells and to show incorporation into VSV pseudotypes. GAPDH and VSV-M were used as loading controls for producer cells and viral pseudotypes, respectively. Western blot analysis was performed once.

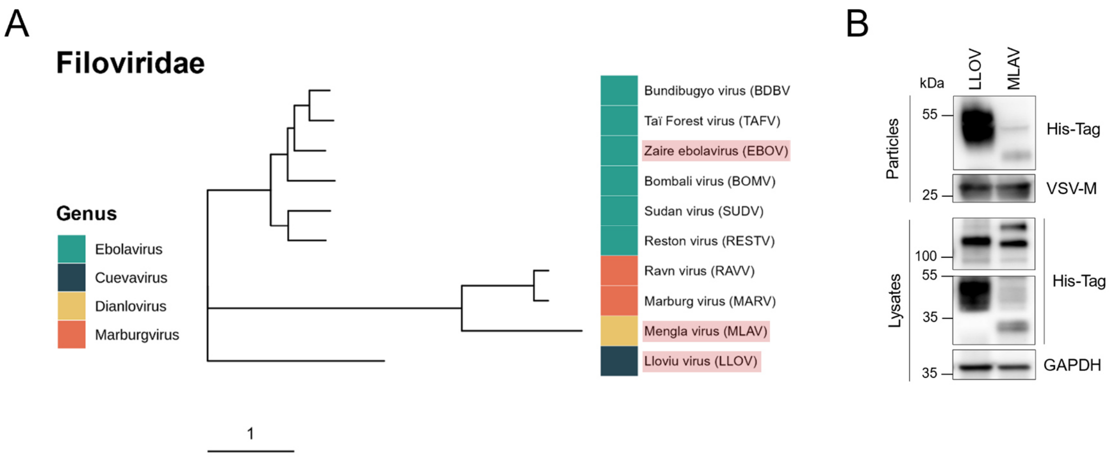

Figure S12. *Filoviridae* G protein phylogeny and Western Blot confirmation of correct pseudotype production. A. Maximum-likelihood phylogenetic tree. Species included in our analysis are shaded in red. **B.** A His-Tag added to the G protein C-terminus was used for detection in producer cells and to show incorporation into VSV pseudotypes, except for Zaire ebolavirus, for which incorporation was shown by assaying pseudotype infectivity. GAPDH and VSV-M were used as loading controls for producer cells and viral pseudotypes, respectively. Western blot analysis was performed once.

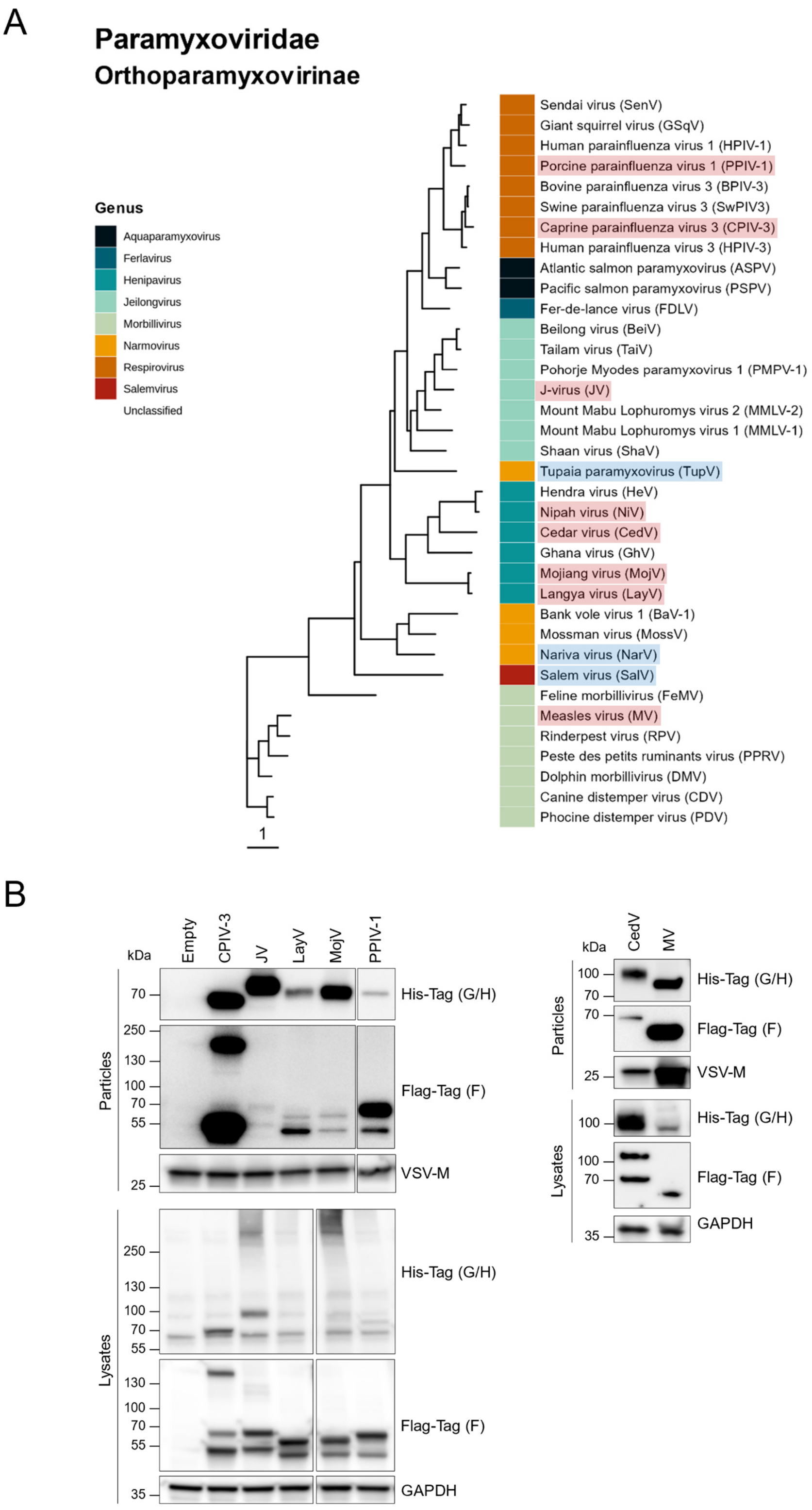

Figure S13. ***Orthoparamyxovirinae*** **subfamily attachment glycoprotein (G/H/Hn) and fusion protein (F) phylogeny and Western Blot confirmation of correct pseudotype production. A.** Maximum-likelihood phylogenetic tree of the protein concatenate. Species included in our analysis are shaded in red. Species shaded in blue are viruses for which pseudotype production was attempted but unsuccessful. **B.** A His-Tag added to the G/H/Hn protein N-terminus and a Flag-Tag added to the F protein C-terminus were used for detection in producer cells and to show incorporation into VSV pseudotypes, except for Nipah virus, for which incorporation was shown by assaying pseudotype infectivity. GAPDH and VSV-M were used as loading controls for producer cells and viral pseudotypes, respectively. Western blot analysis was performed once.

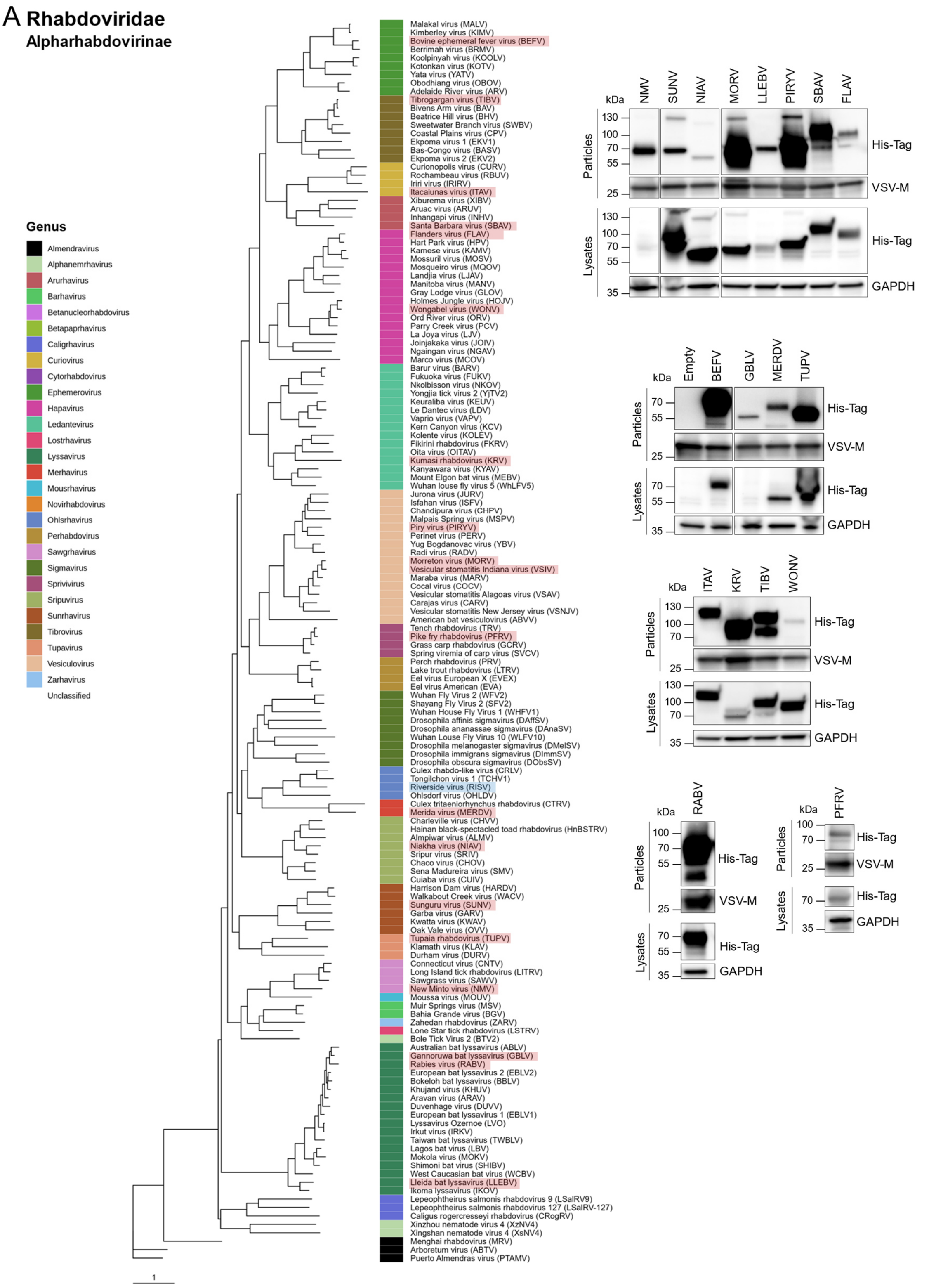

Figure S14. *Apharhabdovirinae* subfamily G glycoprotein phylogeny and Western Blot confirmation of correct pseudotype production. A. Maximum-likelihood phylogenetic tree. Species included in our analysis are shaded in red. Species shaded in blue are viruses for which pseudotype production was attempted but unsuccessful. **B.** A His-Tag added to G protein C-terminus was used for detection in producer cells and to show incorporation into VSV pseudotypes, except for Vesicular stomatitis virus, for which incorporation was shown by assaying pseudotype infectivity. GAPDH and VSV-M were used as loading controls for producer cells and viral pseudotypes, respectively. Western blot analysis was performed once.

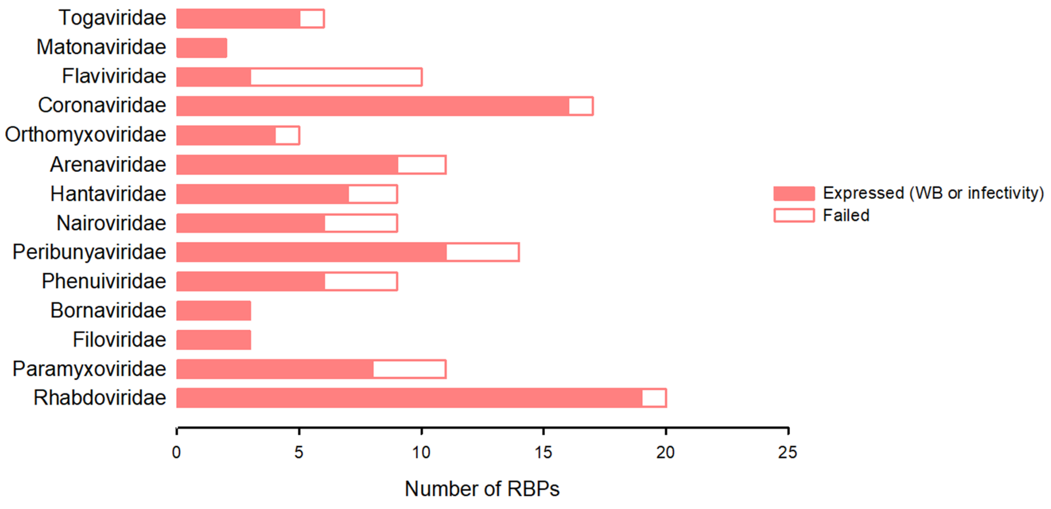

Figure S15. Success rate of VSV pseudotyping for the 129 RPBs. The total number of RPBs in each family and the number of RBPs successfully incorporated into VSV particles is indicated. Successful incorporation was observed by Western blot in 90 cases. In 12 cases, RBP was not detected in Western blots but supernatants showed significant infection of HEK293T, relative to background levels obtained using empty controls (i.e. VSV carrying no RBP). These cases were also considered successful.

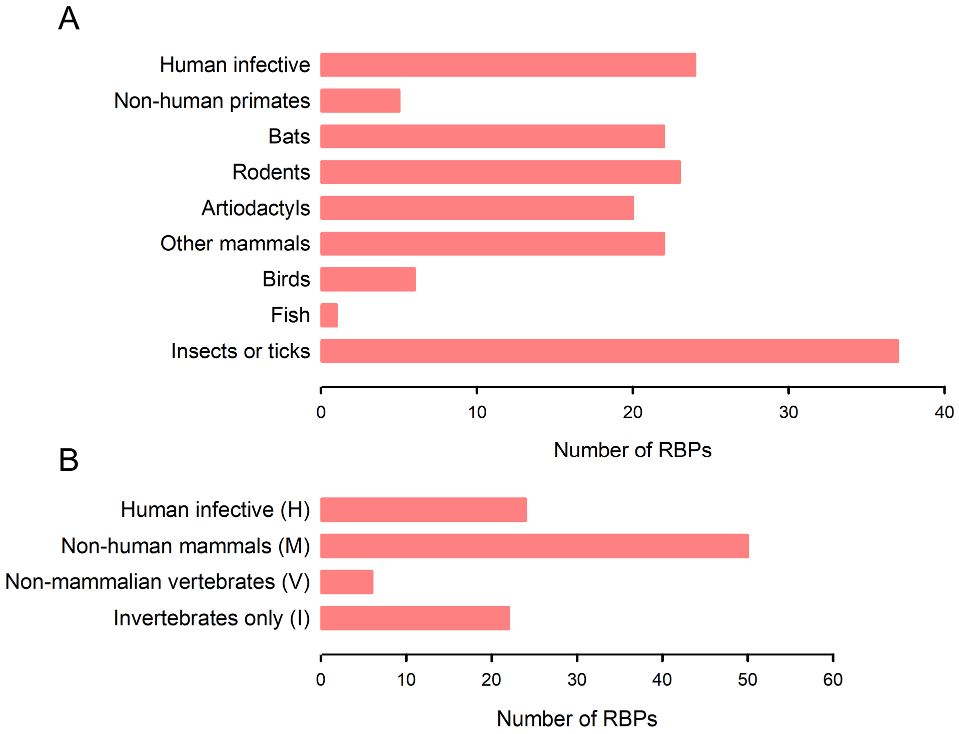

Figure S16. Host information for the 102 successfully pseudotyped RBPs. A. Counts of the number of viruses reported to infect each of the indicated host categories. Notice that each virus can be counted in more than one host category (e.g. insects/ticks and human). PubMed identifiers for articles reporting each association are provided in **Table S2**. **B.** Classification of RBPs in four mutually exclusive reported host categories: human-infective viruses (H), non-human mammals (M), non-mammalian vertebrates (V, i.e. fish or birds), and viruses found only in invertebrates (**I**; i.e. insects or ticks).

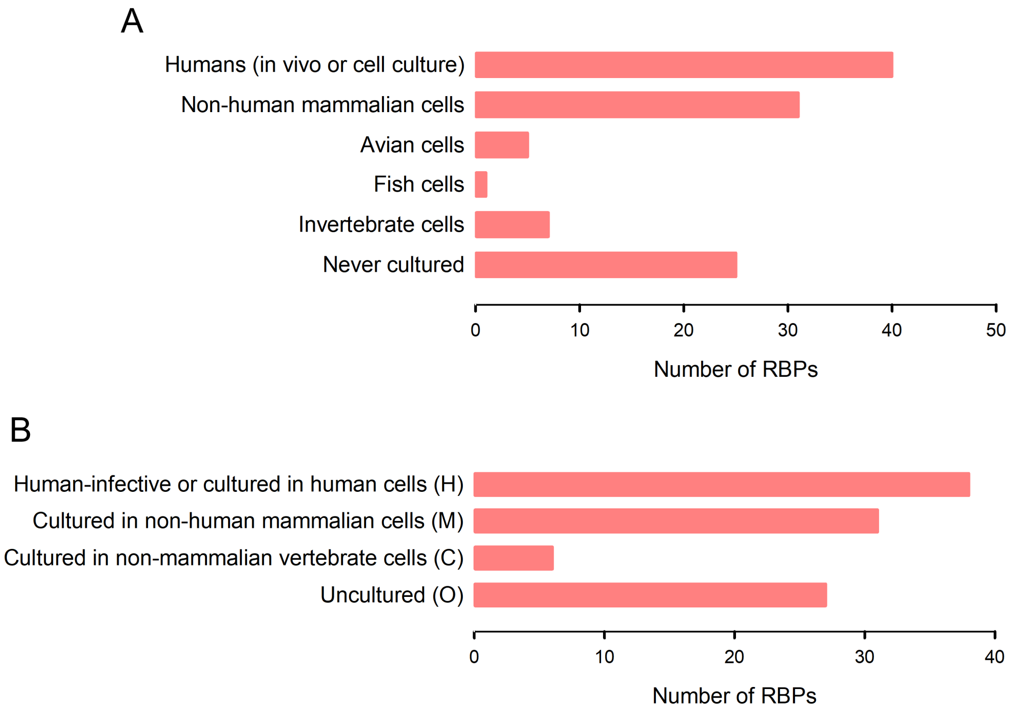

Figure S17. Information about previous viral passaging in cell cultures for the 102 successfully pseudotyped RBPs. A. Counts of the number of viruses cultured in the indicated cell categories. Notice that each virus can be counted in more than one host category. Details and PubMed identifiers for at least one of the reported cell lines (typically the closest to human cells) are provided in **Table S3**. In this Table, information for humans also includes in vivo infections from **Table S2**. **B.** Classification of RBPs in four mutually exclusive categories: human-infective viruses or cultured in human cells (H), cultured in non-human mammalian cells (M), cultured in non-mammalian vertebrate cells (V), and uncultured so far (O).

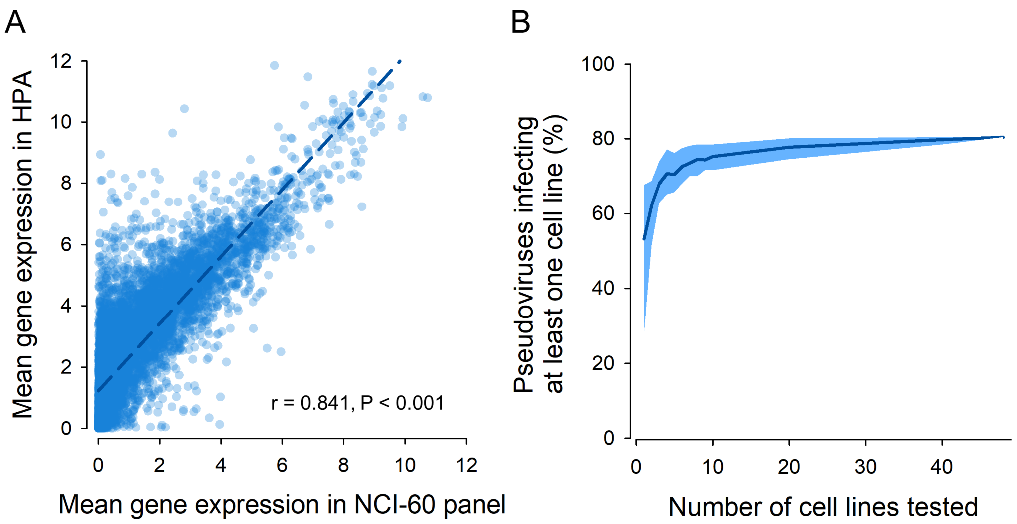

Figure S18. Suitability of the cells lines for investigating human cell entry. A. Correlation between the mean mRNA expression values of cell surface genes in the NCI-60 and Human Protein Atlas (HPA) datasets. The expression values in cell lines from the NCI-60 panel used in this study were compared to those of 41 different human tissues available from HPA. Data were retrieved from the corresponding websites, filtered to consider 7496 annotated as cell surface in Gene Ontology, and expressed as log_2(rpkm+1) and log_2(nTPM+1), respectively. Pearson r coefficient and p-value are indicated (two-sided test; $P < 0.0001$). **B.** Effect of the number of cells tested on estimated human cell infectivity. The number of pseudotypes infecting at least one cell line was calculated as a function of the number of cell lines considered. The dark blue line indicates the average of all possible cell line combinations and the lighter blue shade indicates the range corresponding to the 5th and 95th percentiles. The percentage of pseudotypes infecting at least one cell line rapidly plateaued.

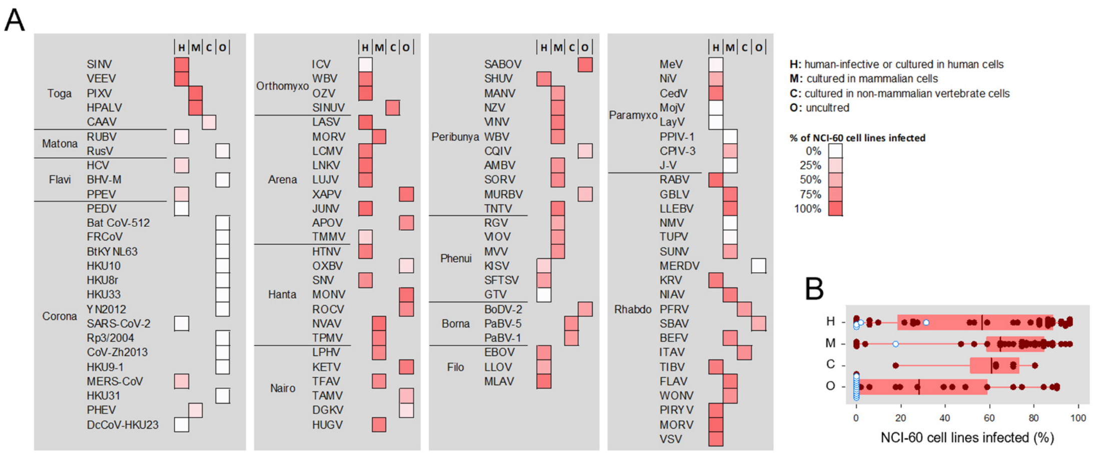

Figure S19. Percentage of cell lines infected as a function of the information about previous viral passaging. A. The percentage of NCI-60 cell lines infected by each viral pseudotype is shown in shades of red. Viruses are classified by family and in four groups: human-infective viruses or cultured in human cells (H), cultured in non-human mammalian cells (M), cultured in non-mammalian vertebrate cells (V), and uncultured so far (O). **B.** Box plot of the percentage of NCI-60 cell lines infected for each of these four groups. Boxes show the median (red line), 25th and 75th percentiles. Horizontal lines departing from boxes indicate the 10th and 90th percentiles. Dots show data points for individual RBPs (n = 102). Coronaviruses are indicated in blue.

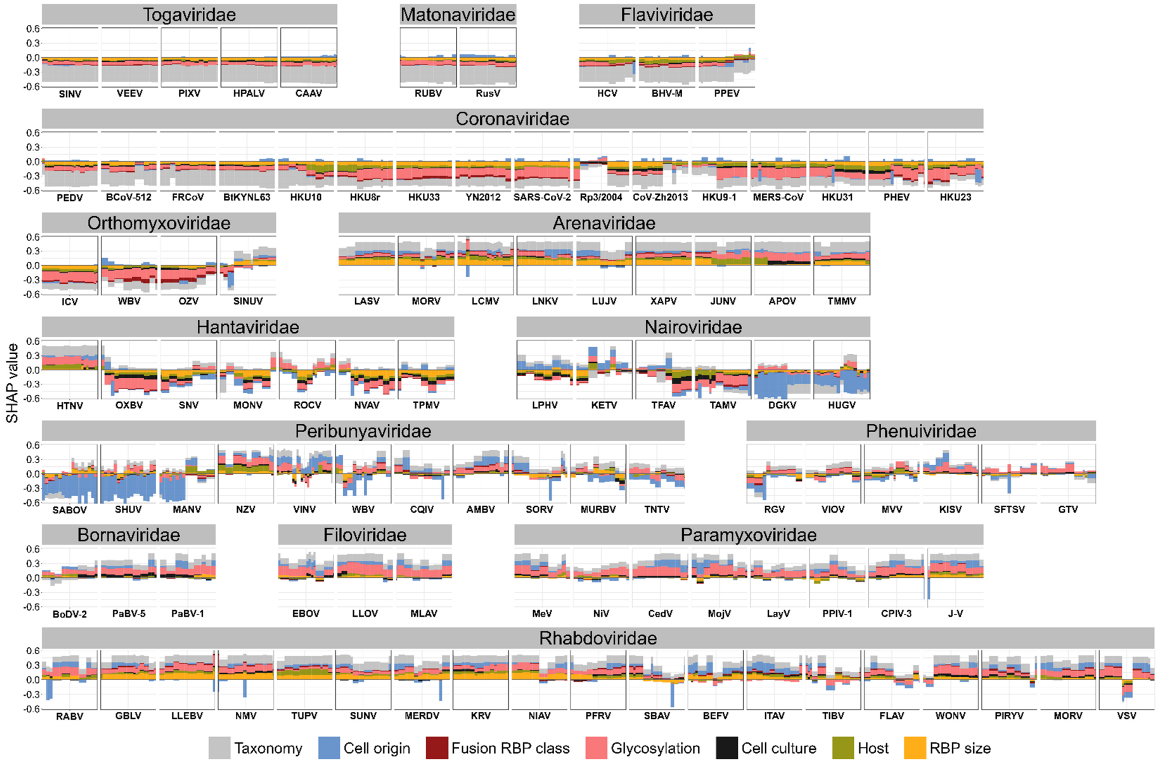

Figure S20. SHAP values from the gradient boosting model. SHAP force plot for the XGBoost model showing how each specific group of features collectively influences predictions for each virus tested. Contributions from different levels of categorical features, as well as N- and O-glycosylation, were aggregated into six final variables (taxonomy, cell origin, fusion RBP class, glycosylation, cell culture, host, and RBP size).

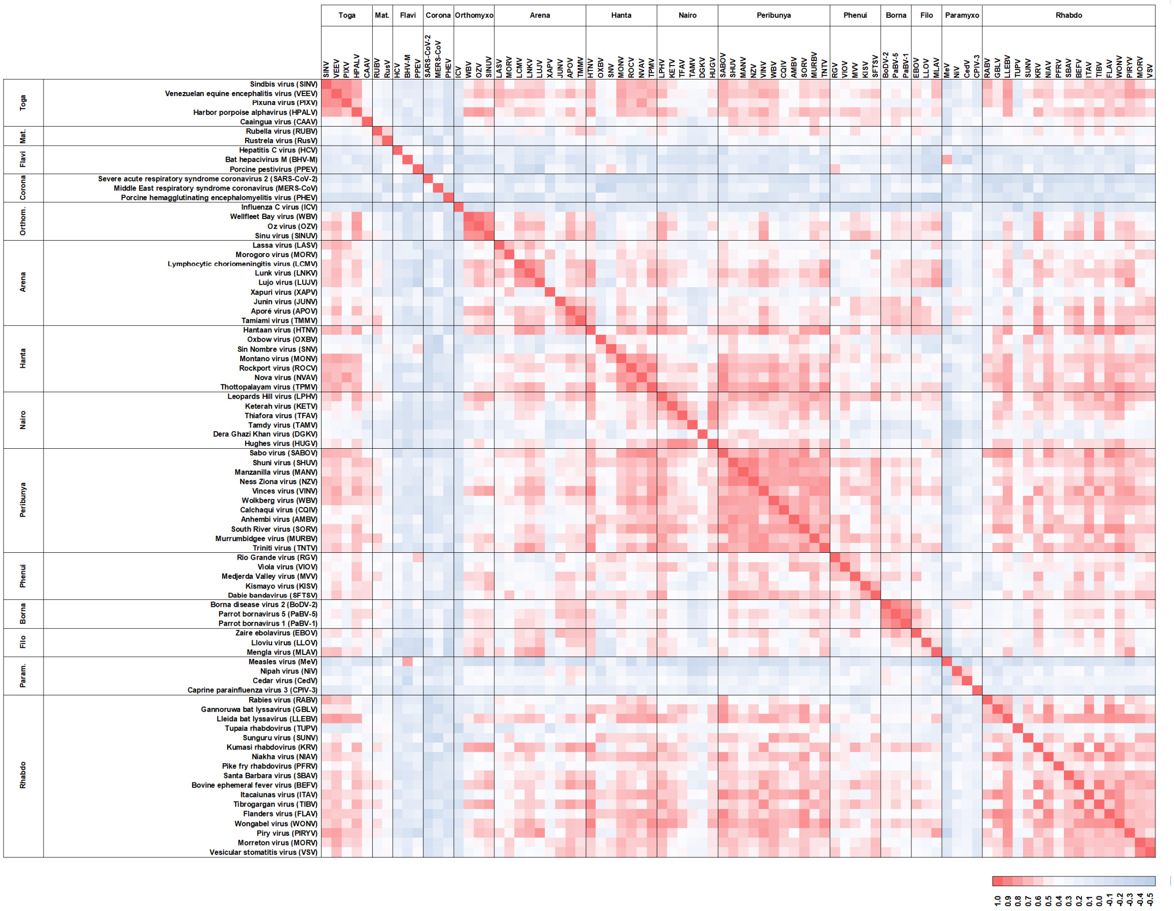

Figure S21. Correlation matrix between pseudotype infectivity across cell lines. The heat map indicates the Pearson correlation between each pair of pseudotypes in terms of their $\log_2(R+1)$ values across the 51 cell lines.

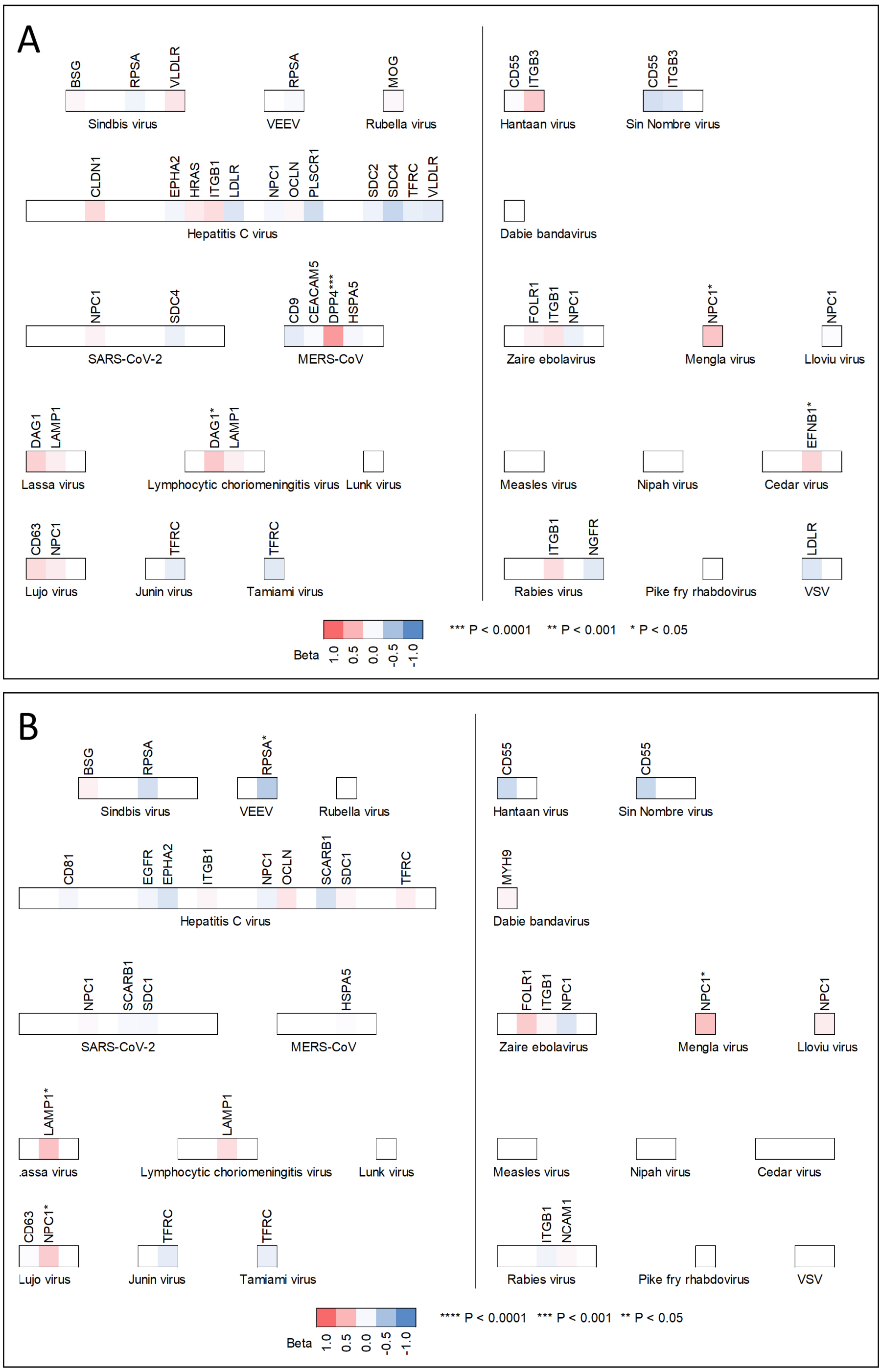

Figure S22. Association between viral entry and the expression levels of known receptors using proteomic data. Multiple regression analysis of pseudotype infectivity as a function of the protein expression levels of known viral receptors quantified using mass spectrometry. These analyses were performed using two independent quantitative proteomic datasets that included 28 (**A**) and 17 (**B**) receptors. Viruses and receptors are shown as in **Figure 5**, but only for receptors with available proteomic data. The rest of combinations are left blank. Colors indicate the standardized regression coefficient. Significant coefficients are indicated with asterisks (two-sided test; **A:** MERS-DPP4: P < 0.0001, LCMV-DAG1: P = 0.0086, Mengla-NPC1: P = 0.0039. Cedar-EFNB1: P = 0.0377; **B:** VEEV-RPSA: P = 0.0044, Lassa-LAMP1: P = 0.0021, Lujo-NPC1: P = 0.0102, Mengla-NPC1: P = 0.0023). The dependent variable was $\log_2(R+1)$ in each of the cell lines, and the independent variables were the protein expression levels measured as log-transformed label free quantification intensities.

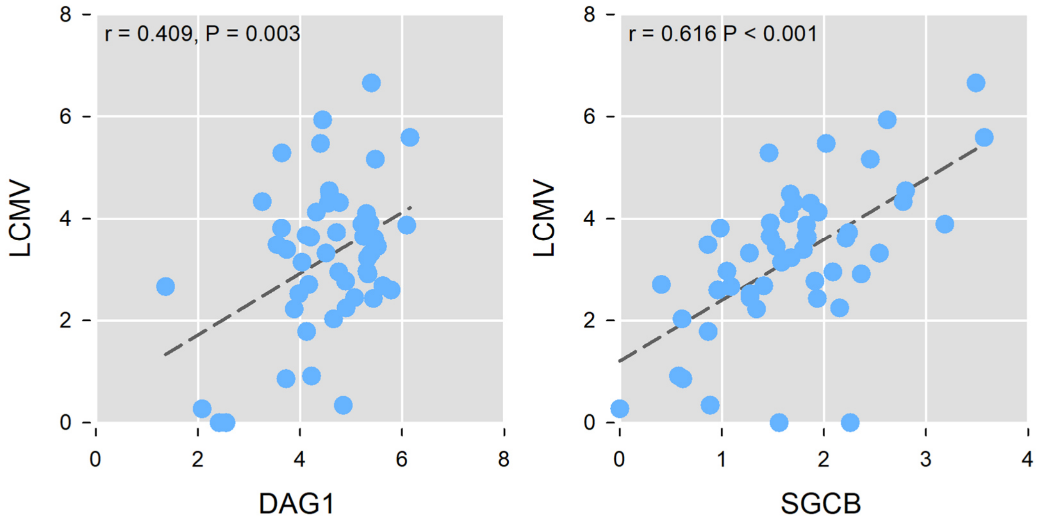

Figure S23. Example of possible indirect determinants of viral tropism. LCMV pseudotype infectivity correlated less strongly with the mRNA expression level of the known DAG1 receptor than with that of SCGB, another component of the dystroglycan complex. Each dot represents a cell line (n = 50). Pearson r coefficients and p-values are indicated (two-sided test; LCMV-DAG1: P = 0.0032; LCMV-SGCB: P < 0.0001).

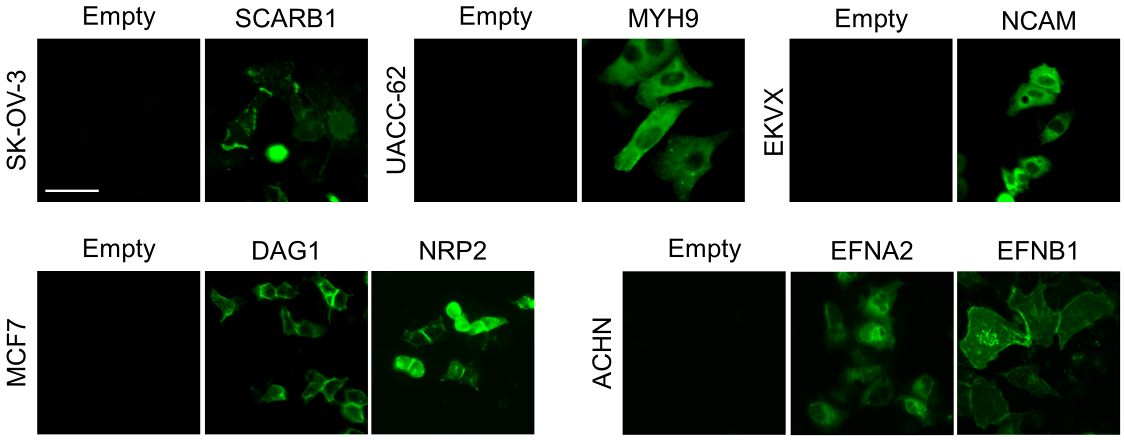

Figure S24. Immunofluorescence analysis of receptor overexpression. The indicated cell lines (SK-OV-3, UACC-62, EKVX, MCF-7, ACHN) were transfected with an empty vector or a Flag-tagged expression construct of the indicated receptor. Receptor overexpression was checked 24 hours post-transfection by immunofluorescence using an anti-Flag antibody. Scale bar: 50 μm. Representative images of one out of two independent experiments are shown.

Supplementary Table 1. Features of the RBPs analyzed in this study (Excel format). The viral family, virus name, RBP name, accession number, fusion protein class, protein size, predicted N-glycosylation and O-glycosylation sites scaled to protein size, range of the amino acids used for RBP reconstruction, tag used for protein detection, and validation of RBP incorporation by western blot or infectivity are provided.

Supplementary Table 2. Host information (Excel format). For each of the 102 successfully pseudotyped viruses, a relevant PubMed identifier providing direct evidence of infection (virus isolation, PCR or sequencing) in humans, non-human primates, bats, rodents, artiodactyls, other mammals, other vertebrates (bird or fish) and arthropods is provided.

Supplementary Table 3. Virus passaging information (Excel format). For each of the 102 successfully pseudotyped viruses, names of cell lines previously used for viral passaging are indicated. Cells are classified depending on their origin (human, non-human primate, non-primate mammalian, avian, invertebrate, other). A relevant PubMed identifier (typically reporting the cell line closest to human) is provided for each virus. For humans, information about in vivo infection is also included.

Supplementary Table 4. Pseudotype infectivity (Excel format). For each of the 102 successfully pseudotyped viruses, the percentage of GFP positive HUVEC cells inoculated with the VSV-based pseudotype is shown, and pseudotypes are classified as infectious if the criteria detailed in the Methods section were met ($Q > 0.05$ and GFP signal at least 5-fold higher than in cells inoculated with a bald pseudotype).

Supplementary Table 5. Features of the 51 cell lines of the NCI-60 panel (Excel format). The name of each cell line, tissue of origin, whether the cell line is derived from the neuroectoderm, and age and sex of donor are provided. Additional details are available at dtp.cancer.gov/discovery_development/nci-60/cell_list.htm.

Supplementary Table 6. Pseudotype infectivity in the NCI-60 panel (Excel format). For each cell-RBP combination, the average percentage of infected cells (GFP positive) is provided, along with background signal obtained with empty controls (bald VSV carrying no RBP). All pseudotypes were assayed twice. A positive control in which VSV-DG was loaded with its own RBP was assayed in each experimental block (total 42 replicates). A dichotomous variable indicating the presence or absence of infection are provided, as well as $\log_2(R+1)$ values. Details on the calculation of these variables are provided in the Methods section. Data are shown only for viruses infecting at least one cell type.

Supplementary Table 7. Primers used for cDNA amplification (Excel format). The gene, accession number, cloning method and vector, and primer sequences are provided.

Supplementary Table 8. RNA-sequencing data (Excel format). Gene expression data were obtained from the CellMiner website (discover.nci.nih.gov/cellminer/loadDownload.do, RNA-seq - composite expression file) and expressed as $\log_2(\text{rpkm}+1)$.

Supplementary Table 9. First set of proteomics data (Excel format). Protein expression data were obtained from the CellMiner website (discover.nci.nih.gov/cellminer/loadDownload.do, SWATH Mass spectrometry – Protein file).

Supplementary Table 10. Second set of proteomics data (Excel format). Protein expression data were obtained from ebi.ac.uk/pride/archive/projects/PXD005940.

Uncropped blots – Supplementary Figure 1B

Viral particles

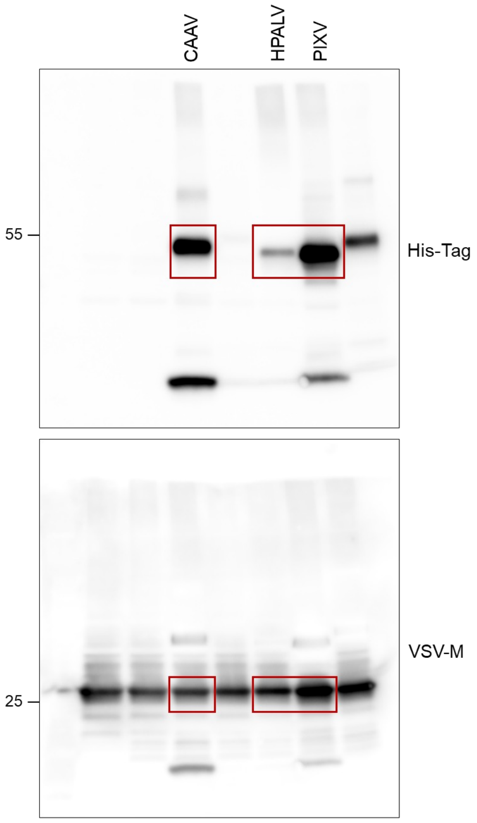

Cell lysates

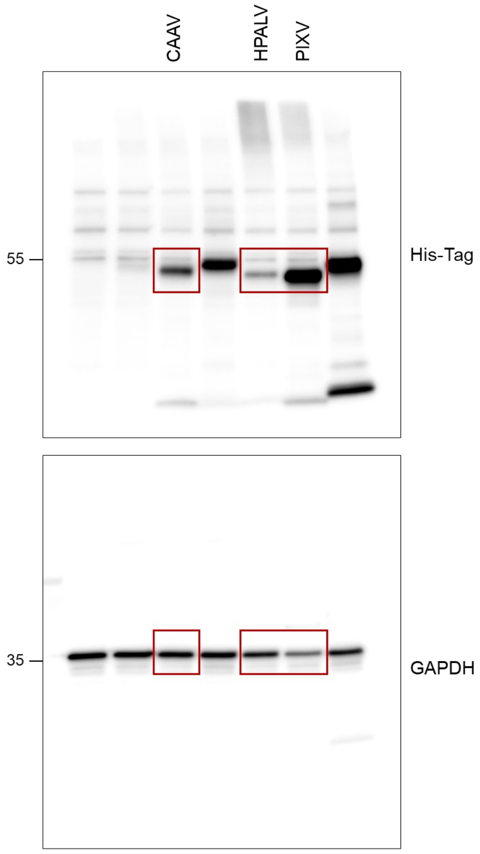

Viral particles

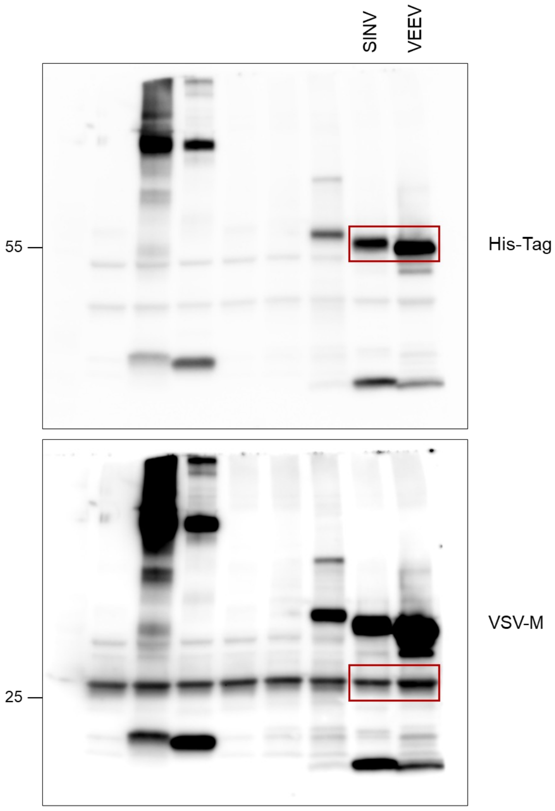

Cell lysates

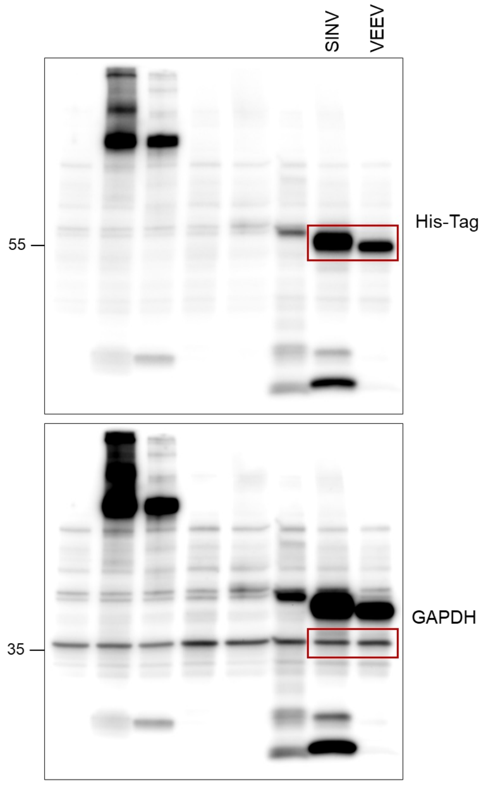

Uncropped blots – Supplementary Figure 2B

Viral particles

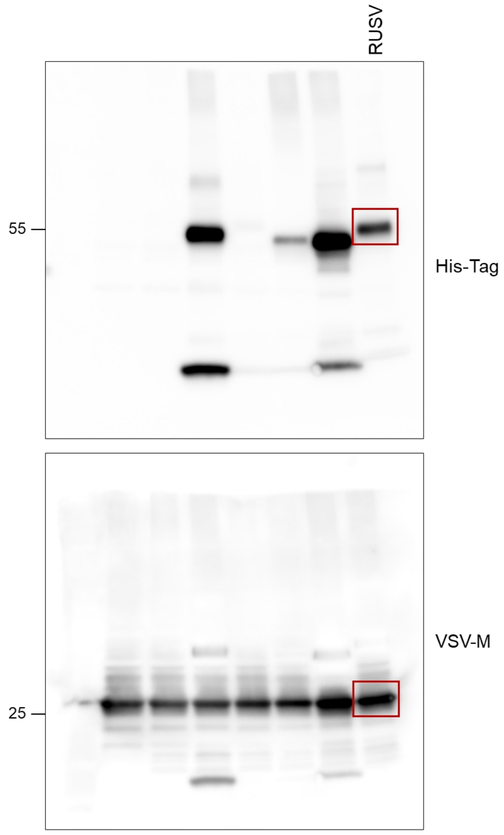

Cell lysates

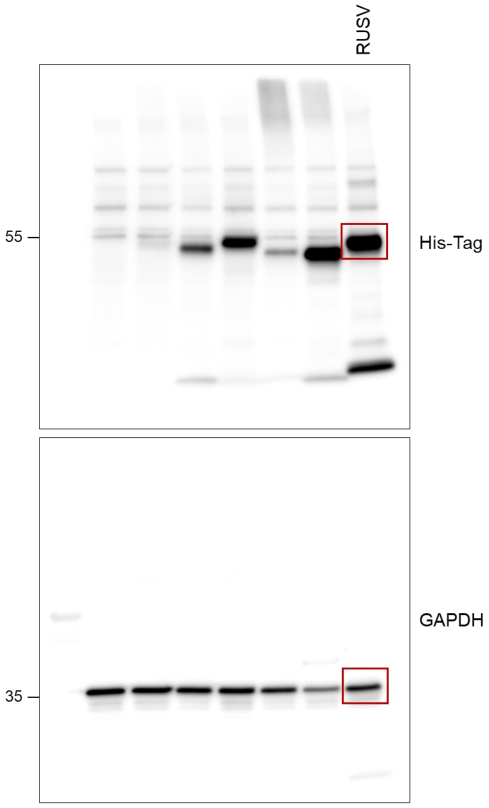

Viral particles

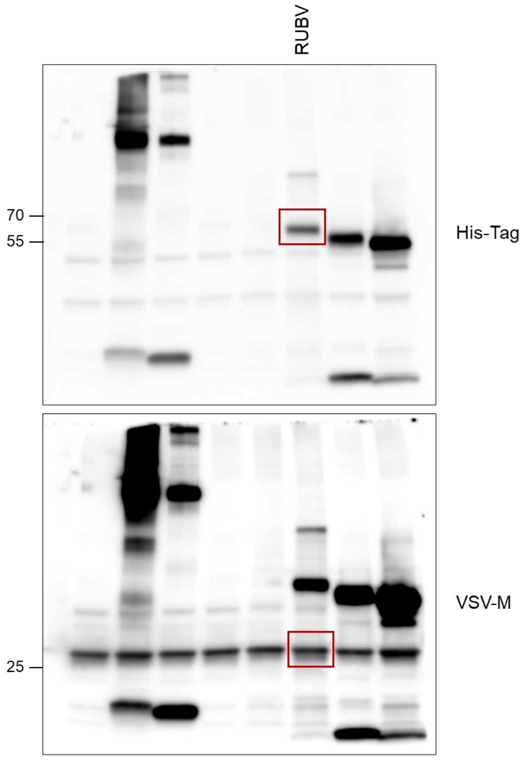

Cell lysates

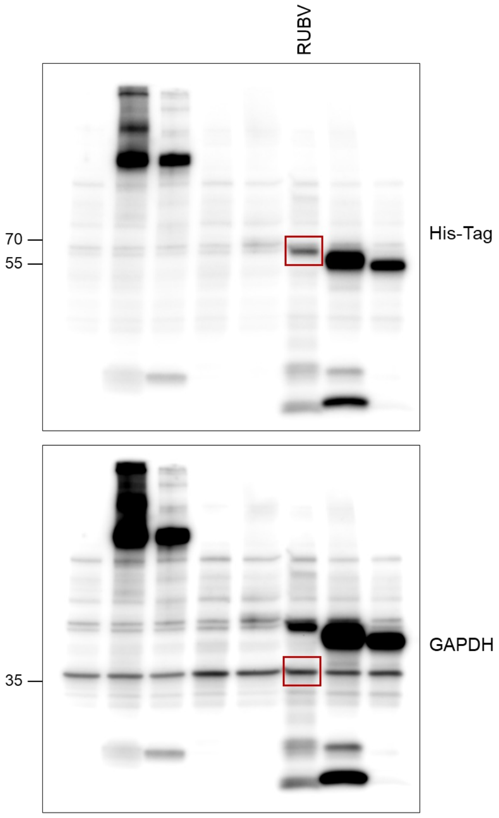

Uncropped blots – Supplementary Figure 3B

Viral particles

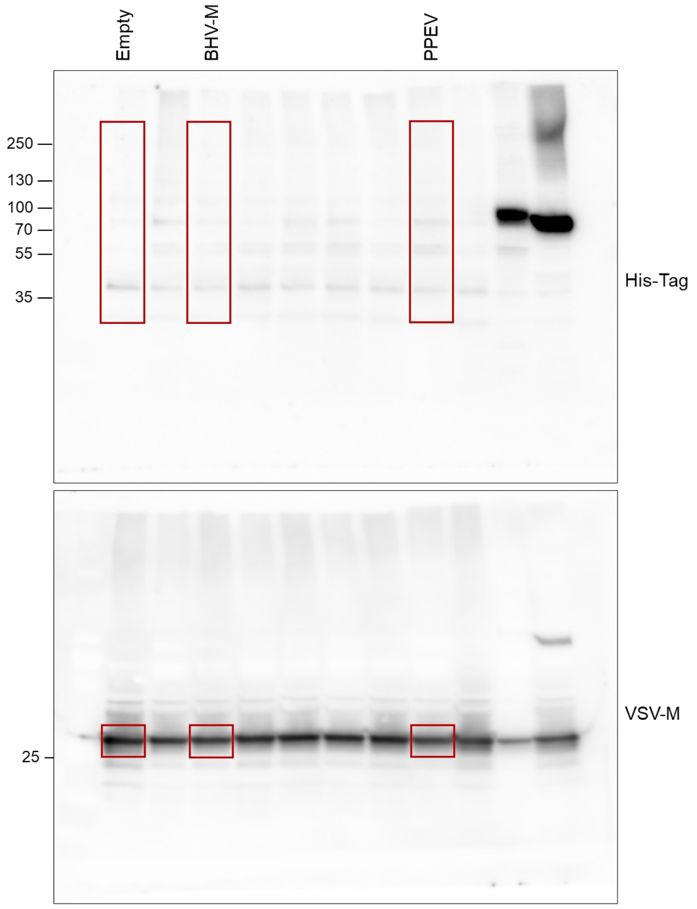

Cell lysates

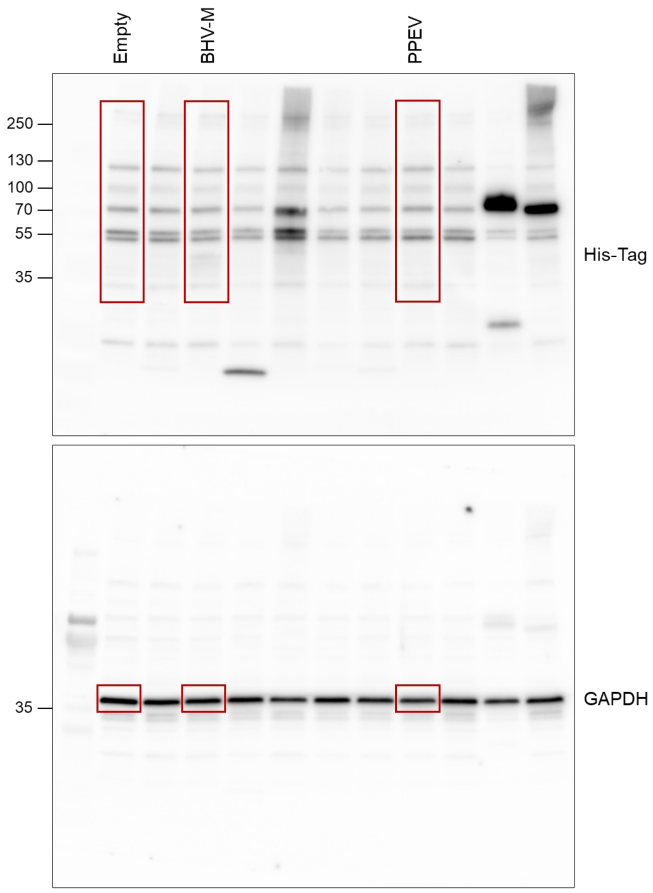

Viral particles

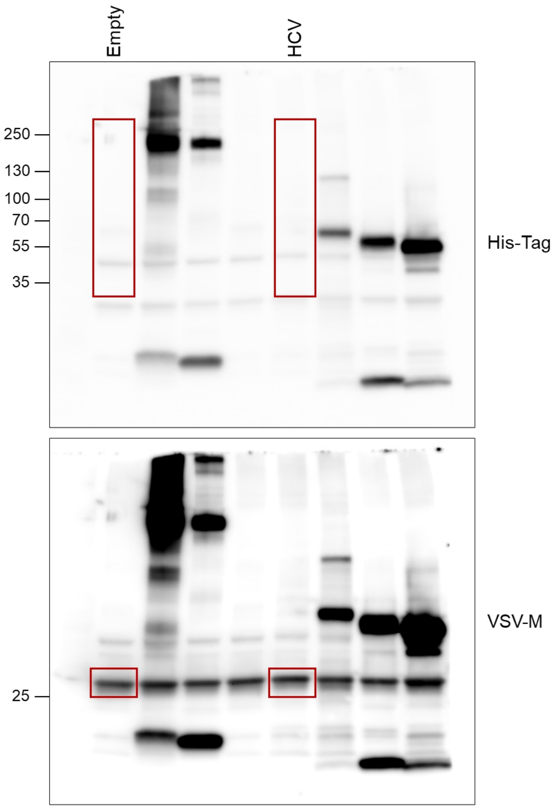

Cell lysates

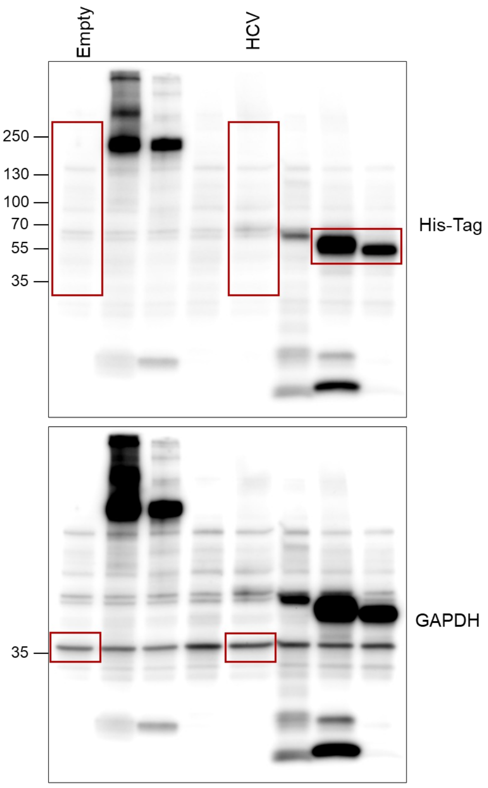

Uncropped blots – Supplementary Figure 4B - 1

Viral particles

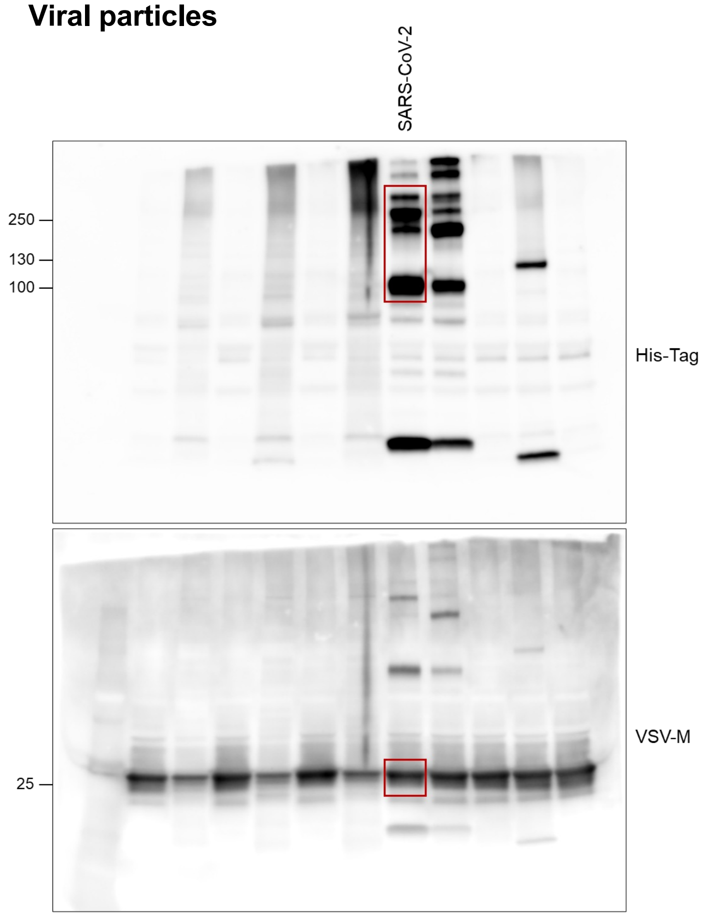

Cell lysates

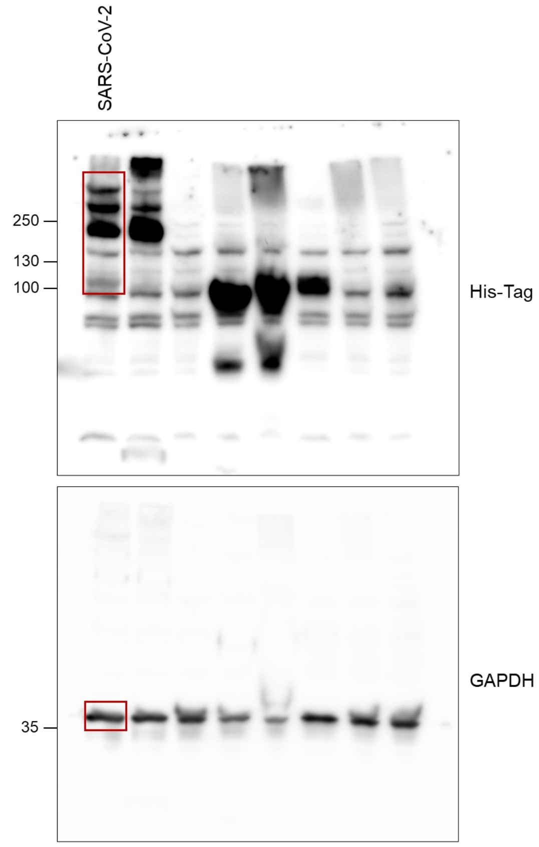

Viral particles

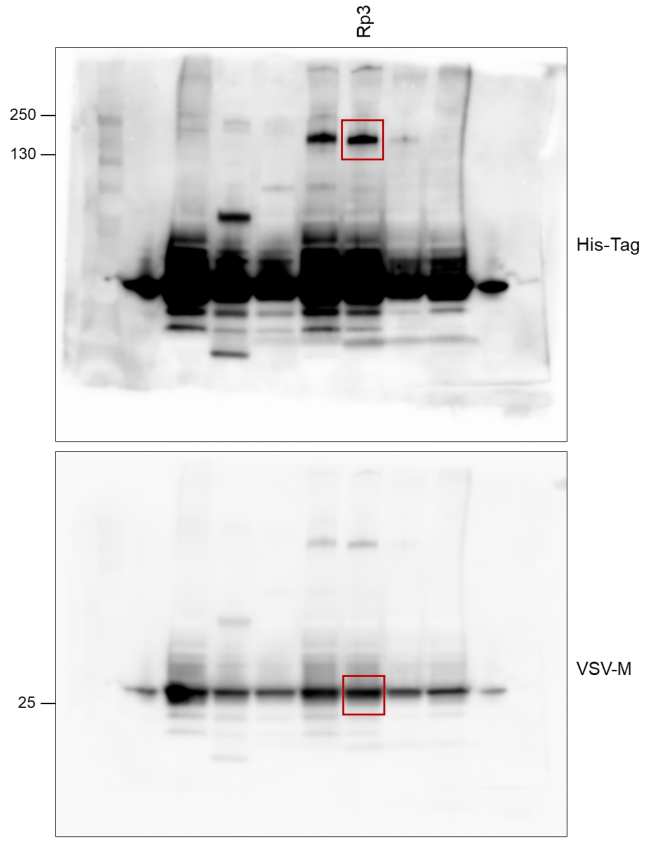

Cell lysates

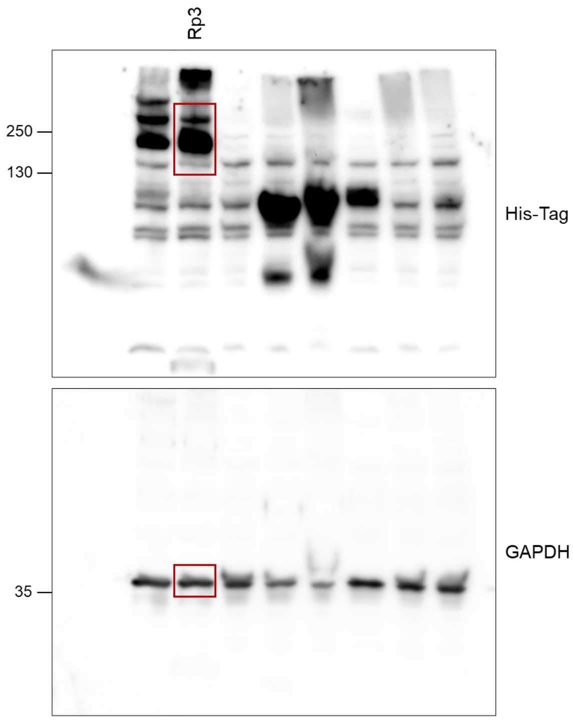

Uncropped blots – Supplementary Figure 4B - 2

Viral particles

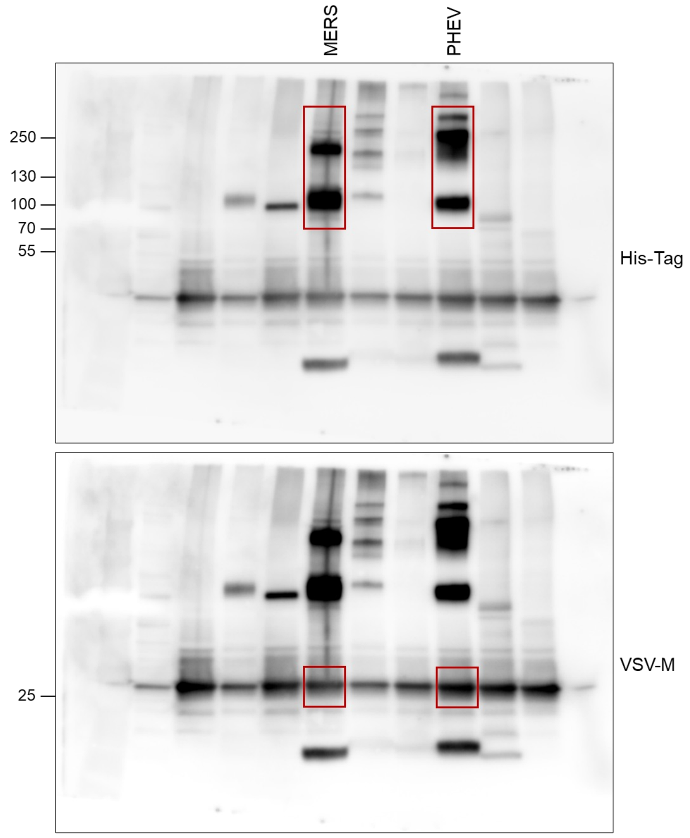

Cell lysates

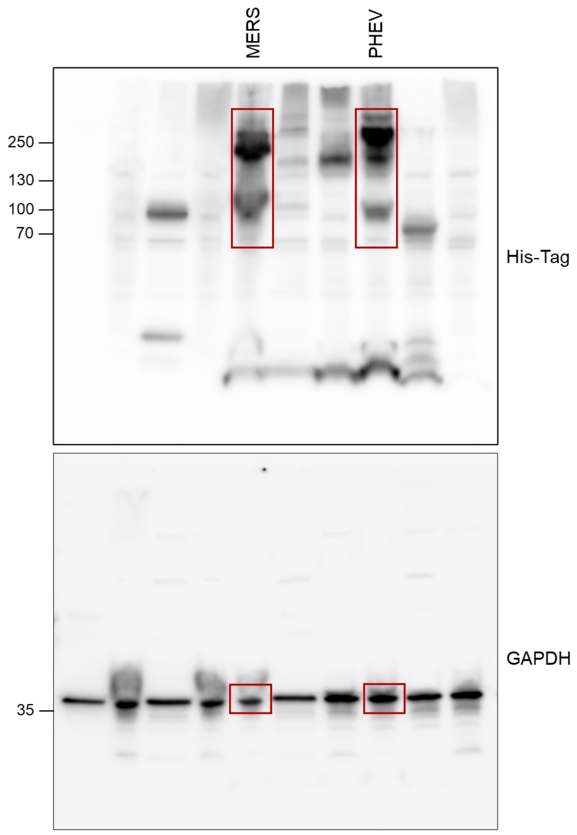

Viral particles

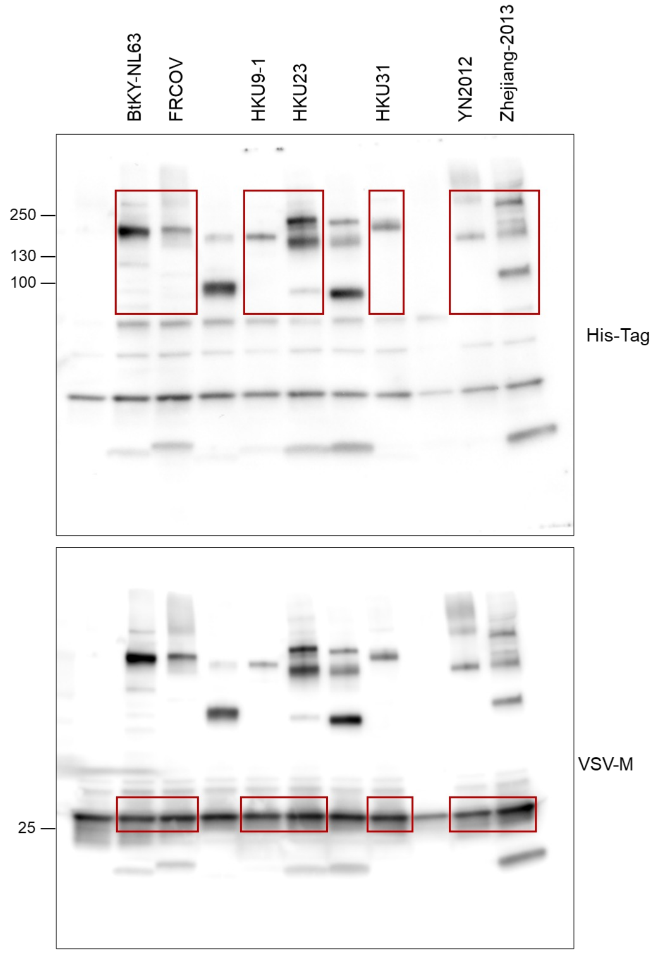

Cell lysates

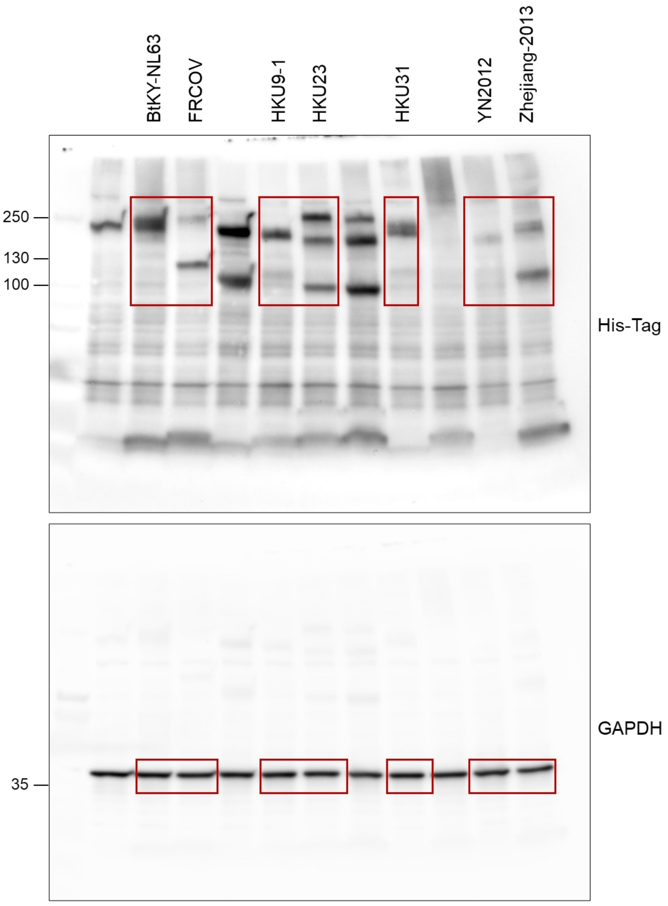

Uncropped blots – Supplementary Figure 4B - 3

Viral particles

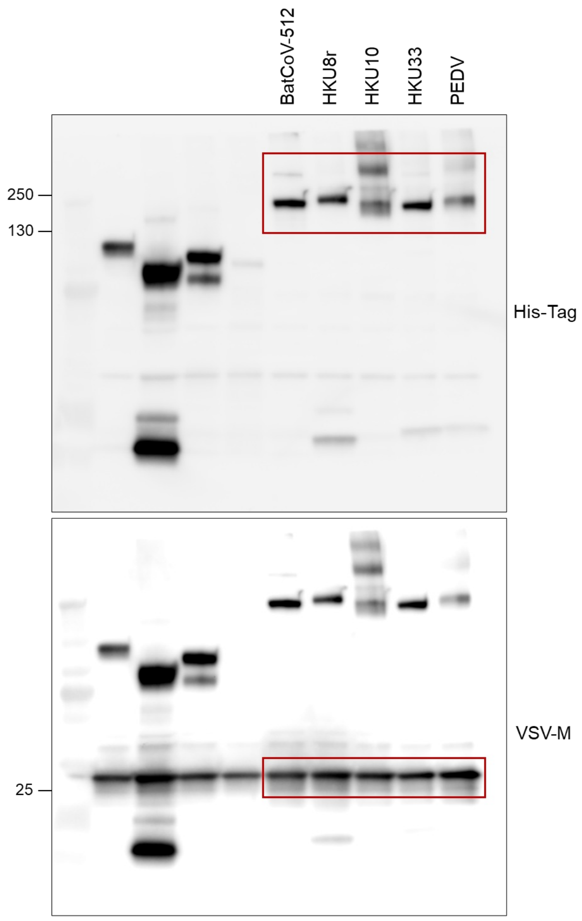

Cell lysates

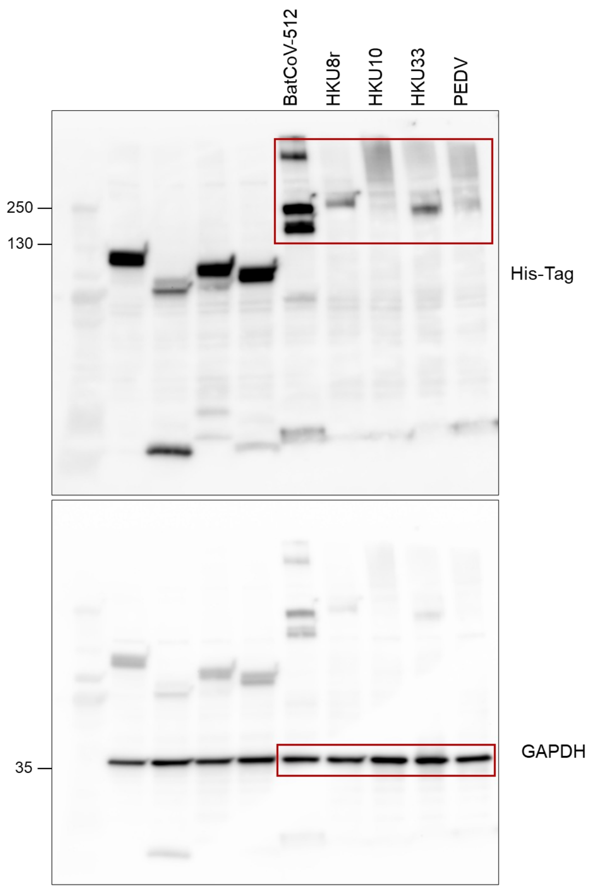

Uncropped blots – Supplementary Figure 5B

Viral particles

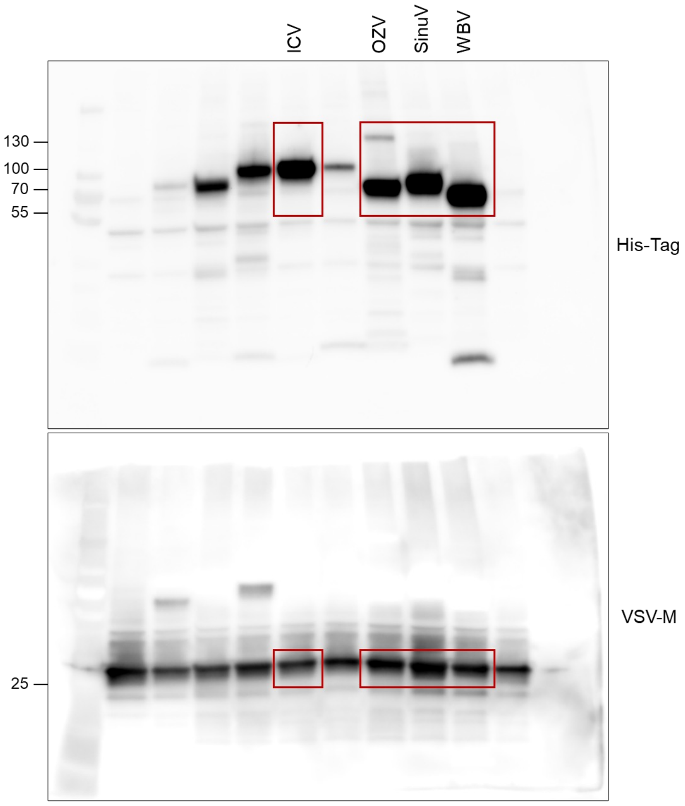

Cell lysates

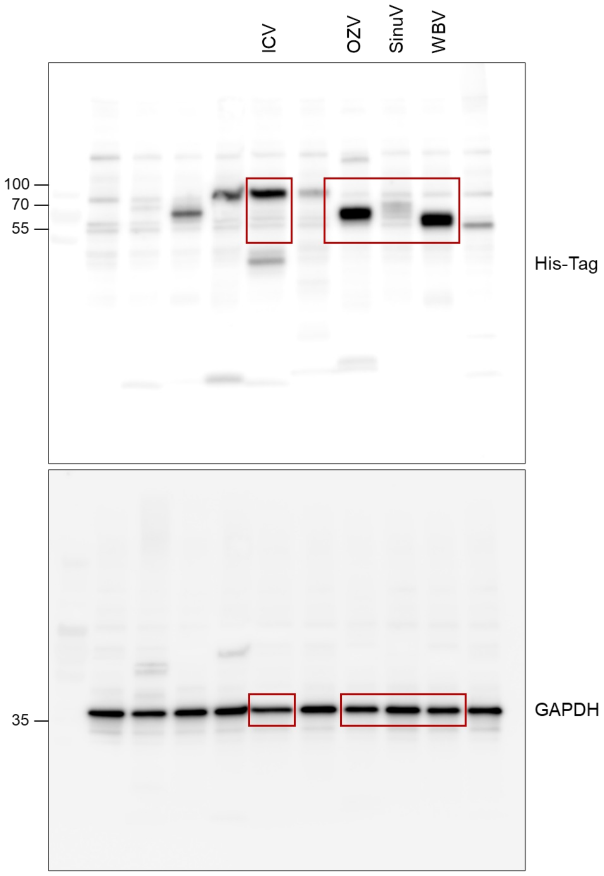

Uncropped blots – Supplementary Figure 6B - 1

Viral particles

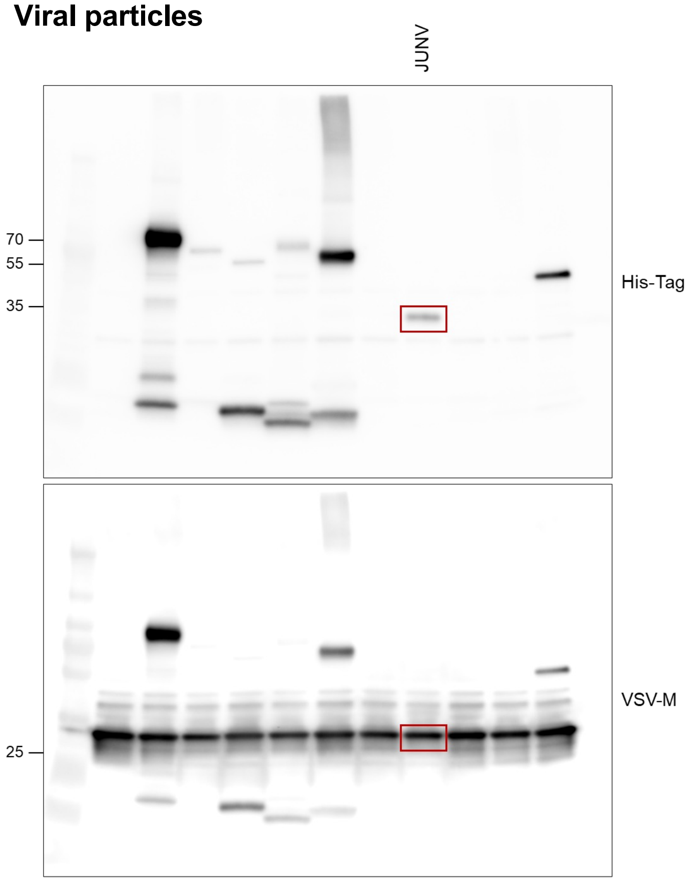

Cell lysates

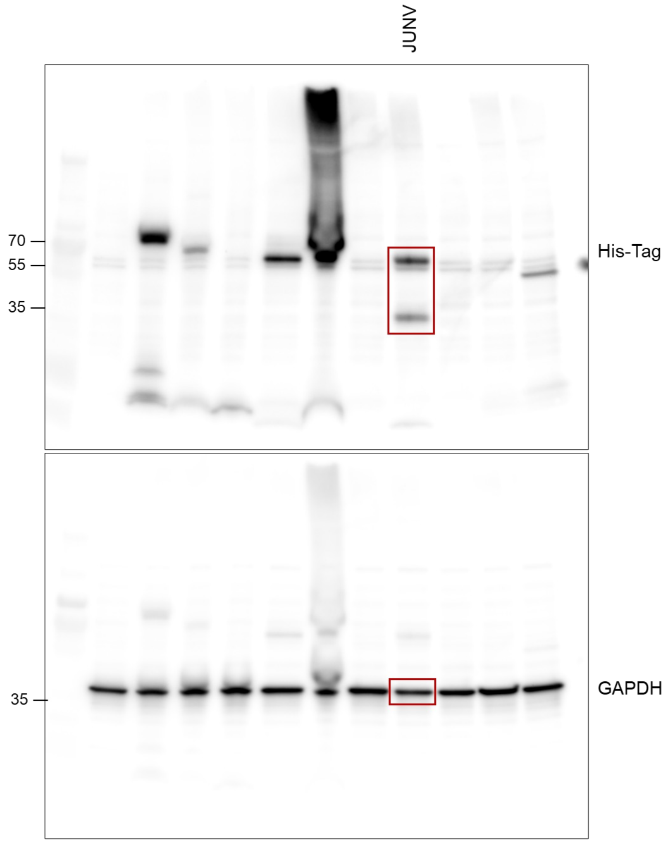

Viral particles

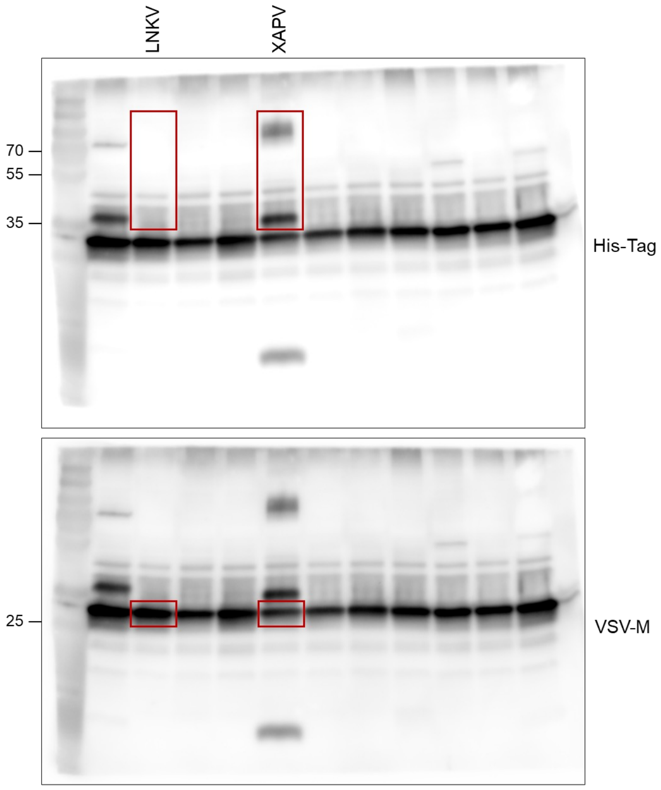

Cell lysates

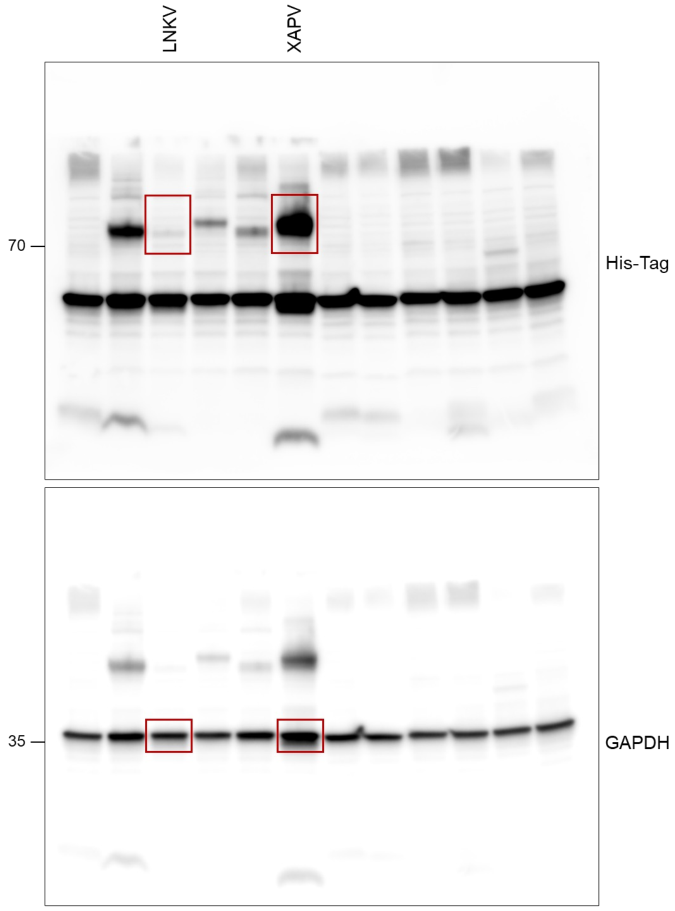

Uncropped blots – Supplementary Figure 6B - 2

Viral particles

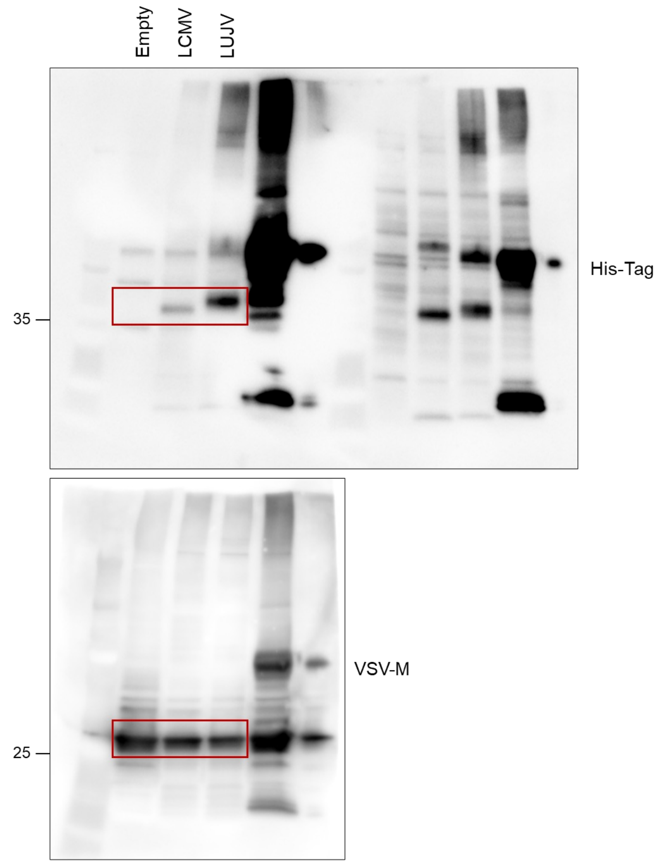

Cell lysates

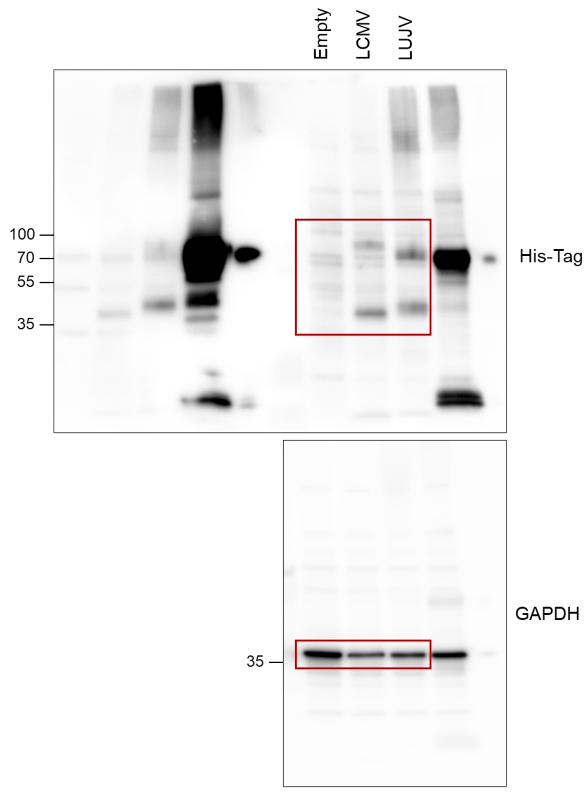

Viral particles

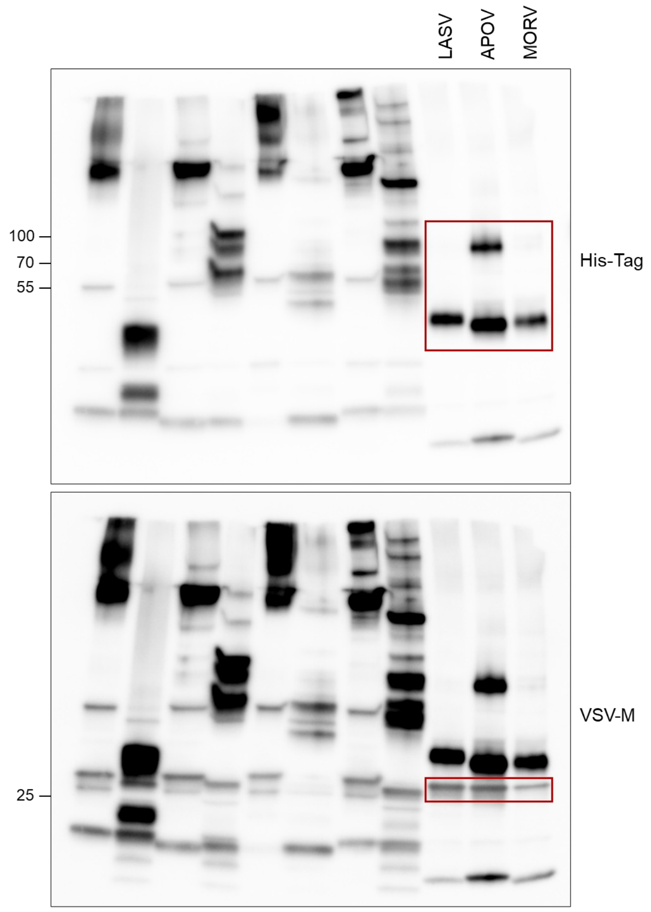

Cell lysates

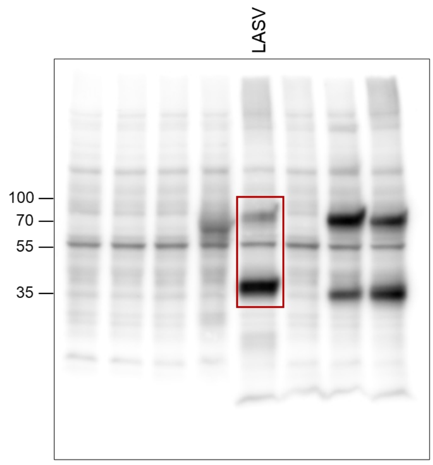

Uncropped blots – Supplementary Figure 6B - 3

Cell lysates

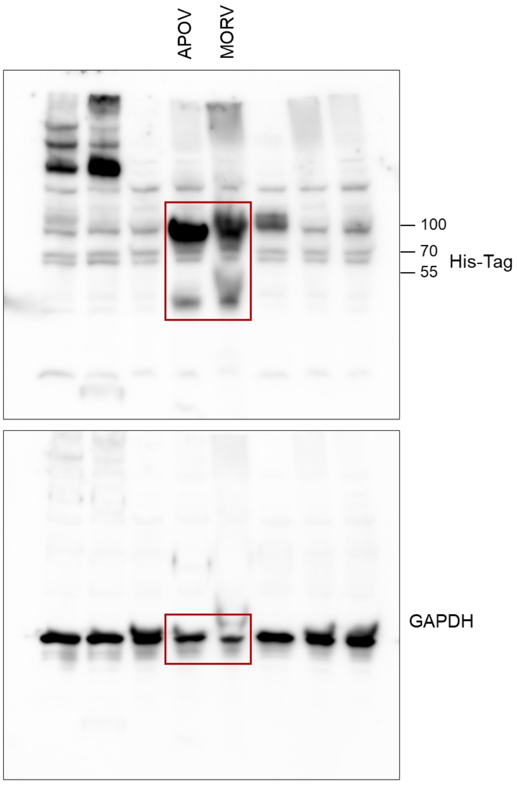

Uncropped blots – Supplementary Figure 7B

Viral particles

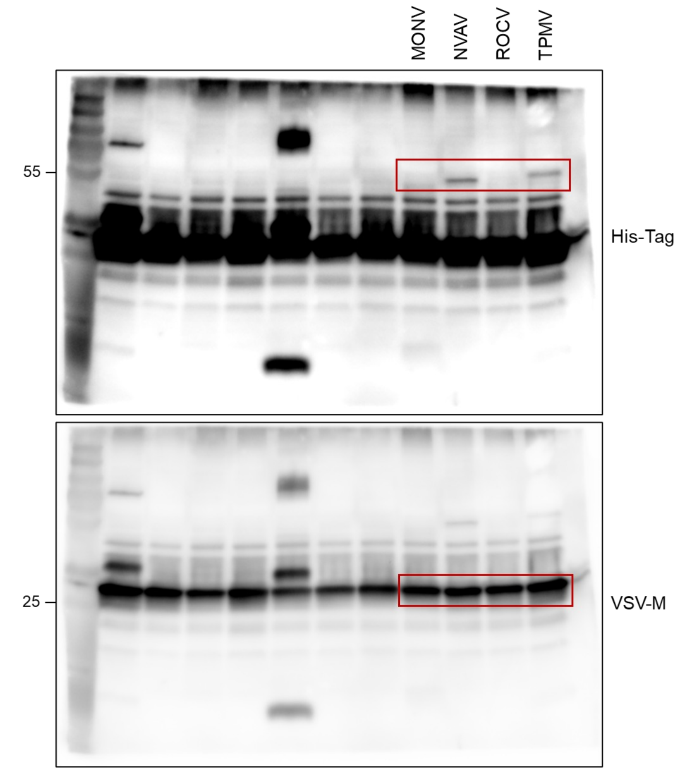

Cell lysates

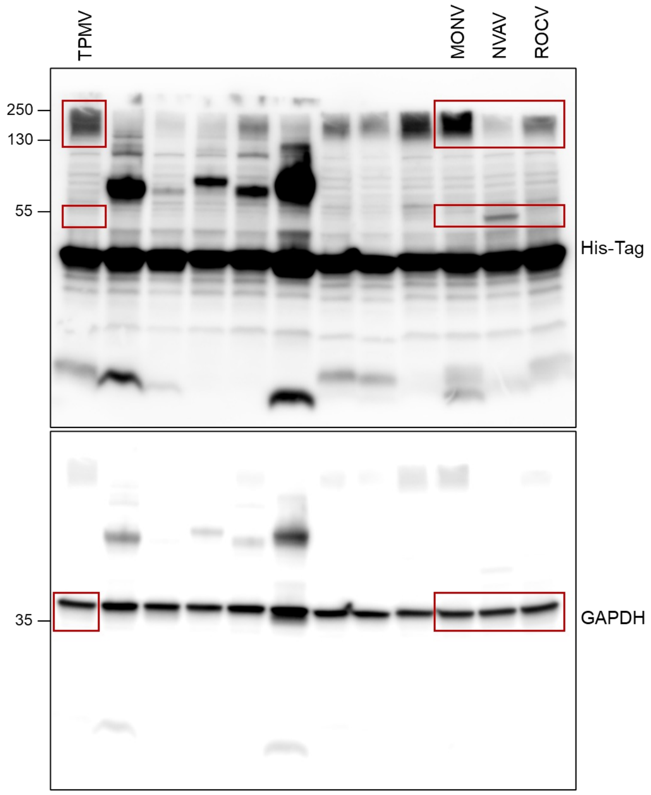

Viral particles

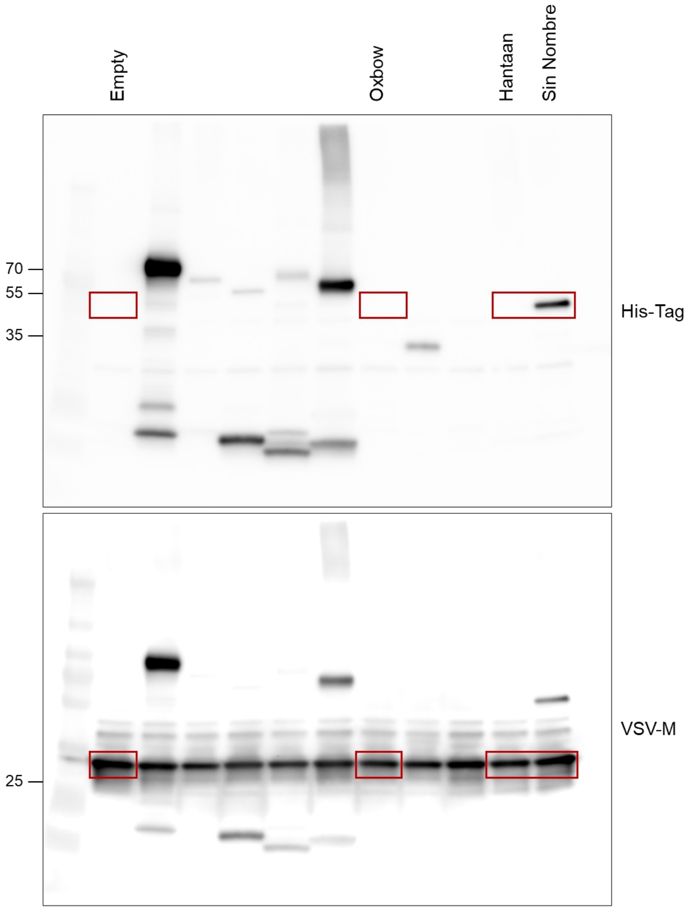

Cell lysates

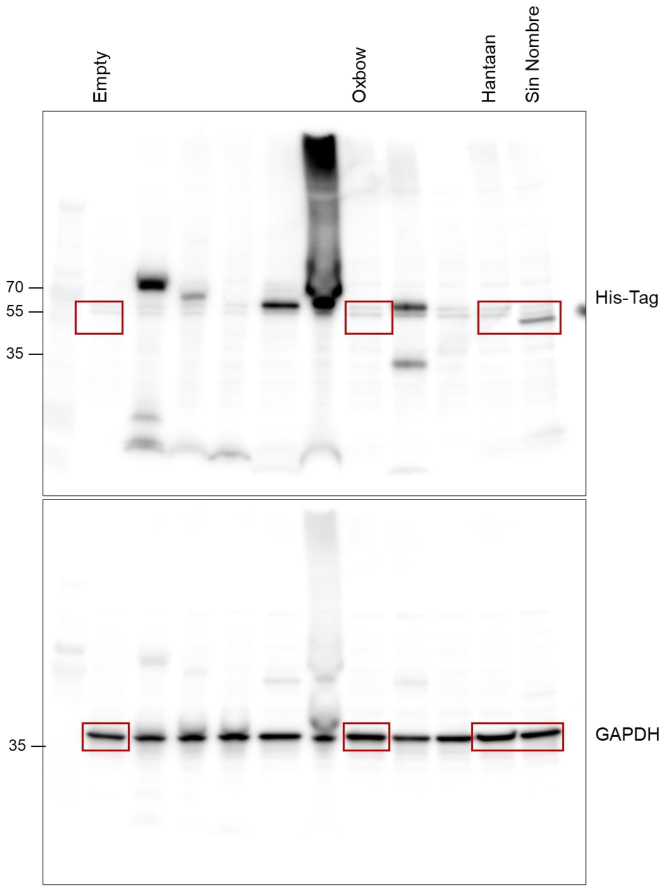

Uncropped blots – Supplementary Figure 8B

Viral particles

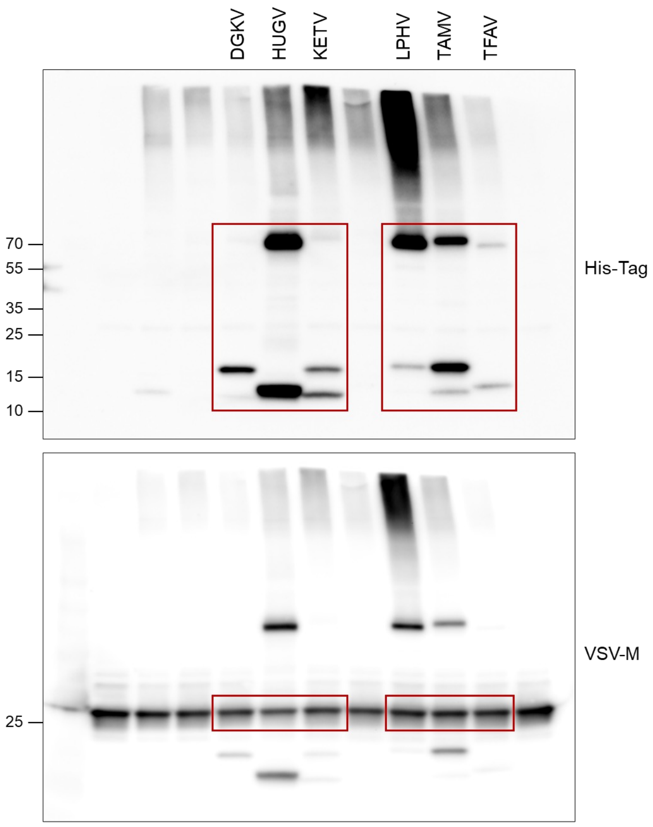

Cell lysates

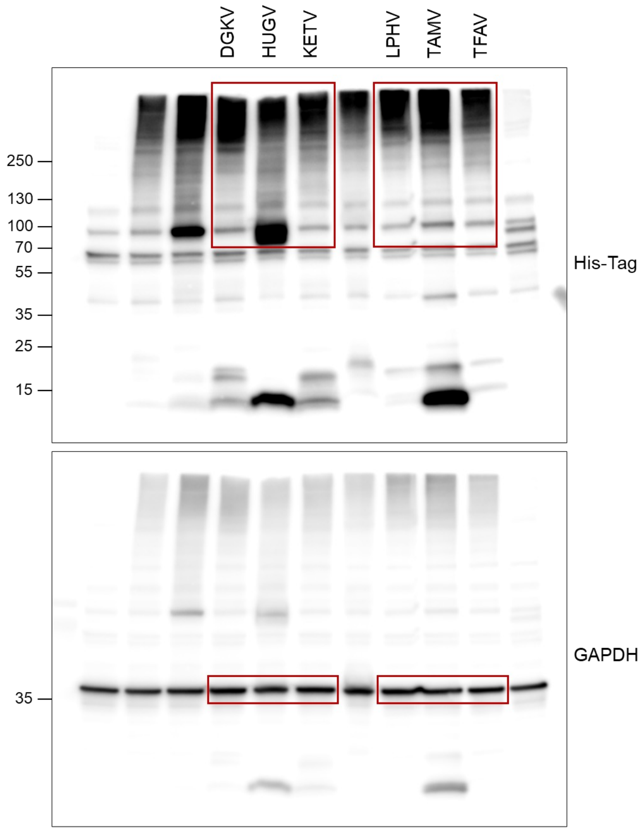

Uncropped blots – Supplementary Figure 9B - 1

Viral particles

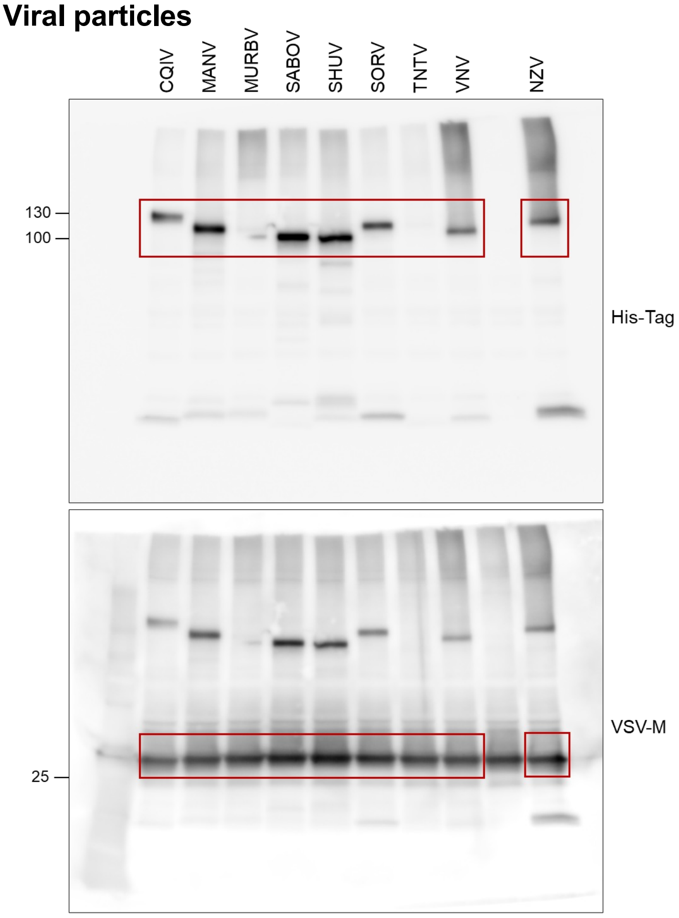

Cell lysates

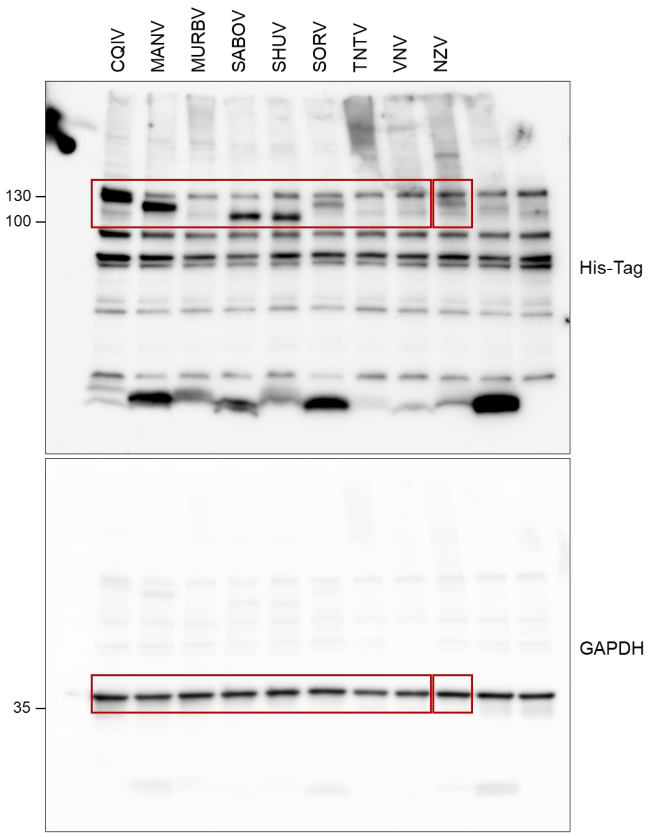

Viral particles

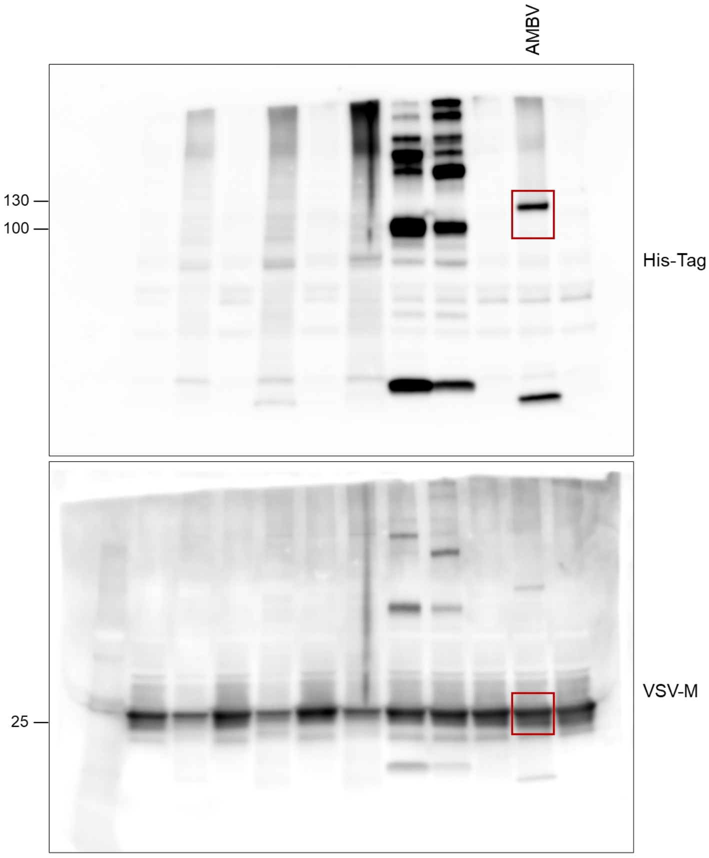

Cell lysates

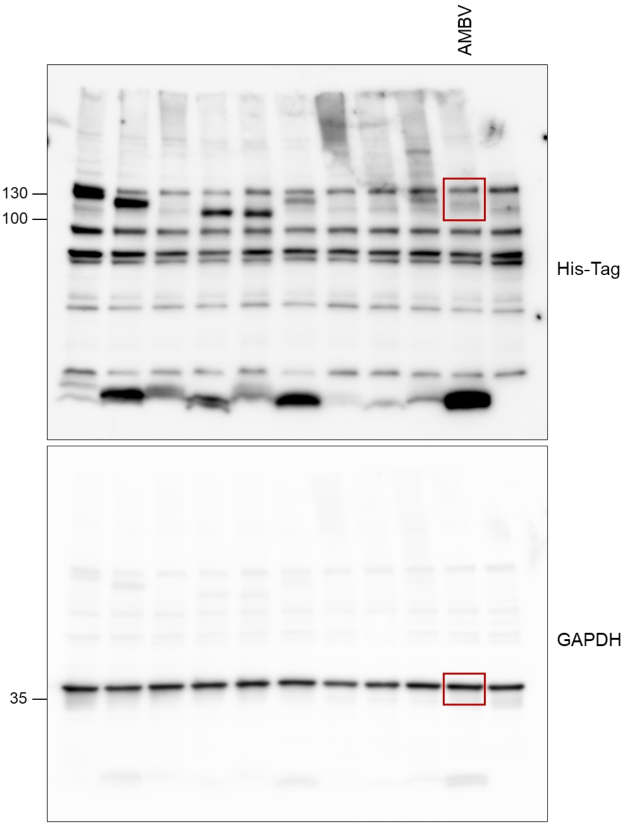

Uncropped blots – Supplementary Figure 9B - 2

Viral particles

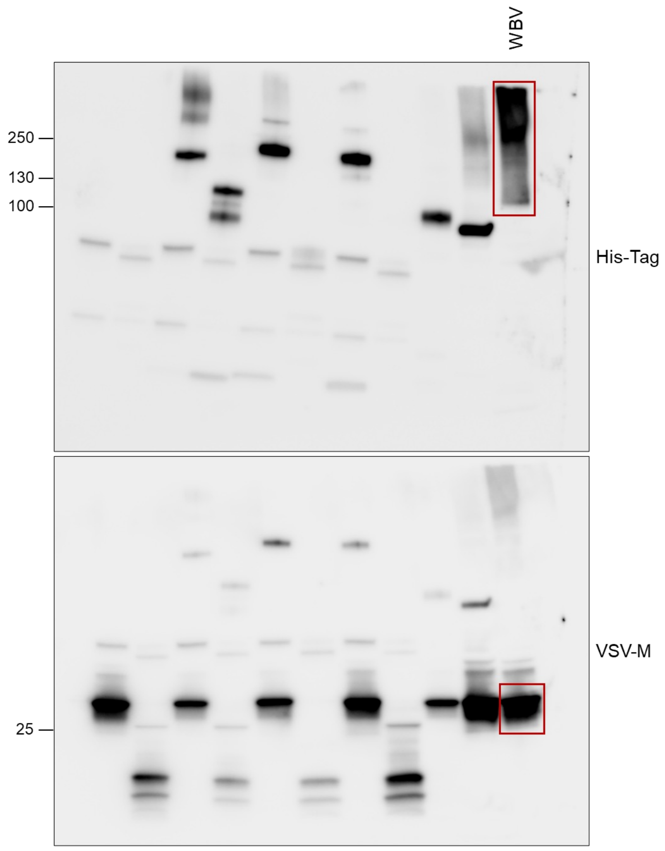

Cell lysates

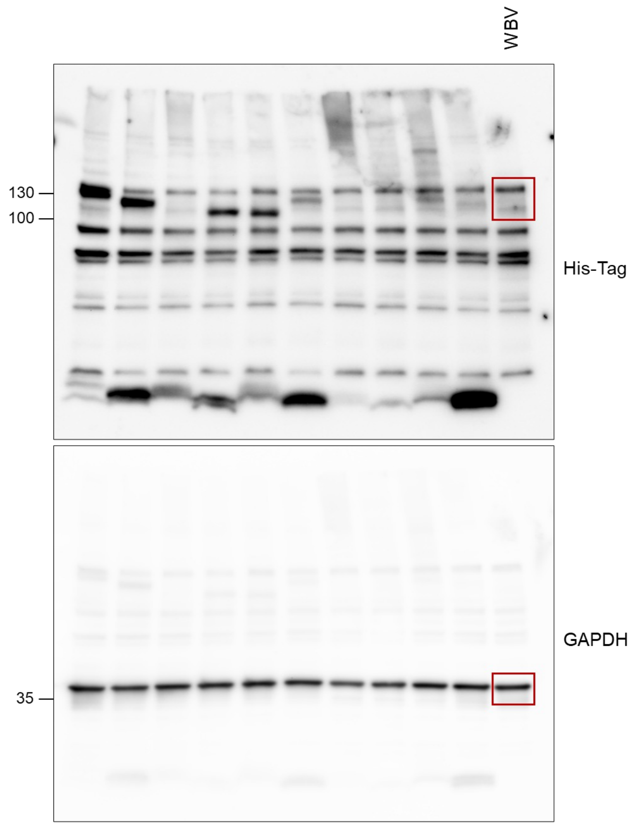

Uncropped blots – Supplementary Figure 10B - 1

Viral particles

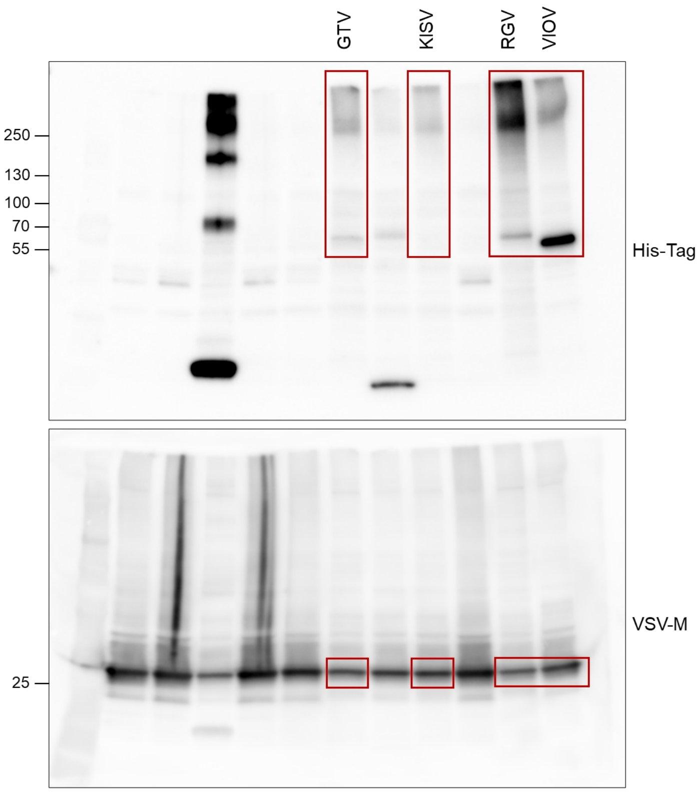

Cell lysates

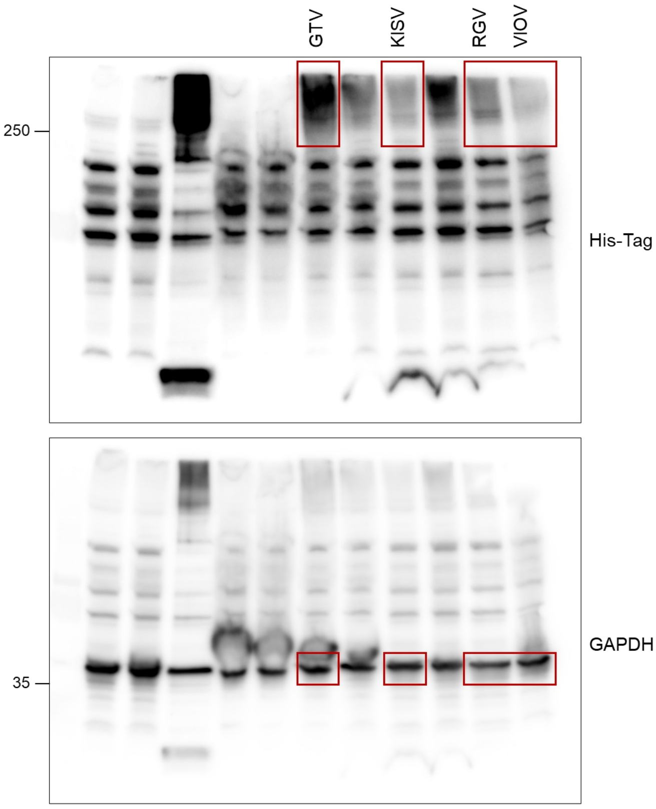

Viral particles

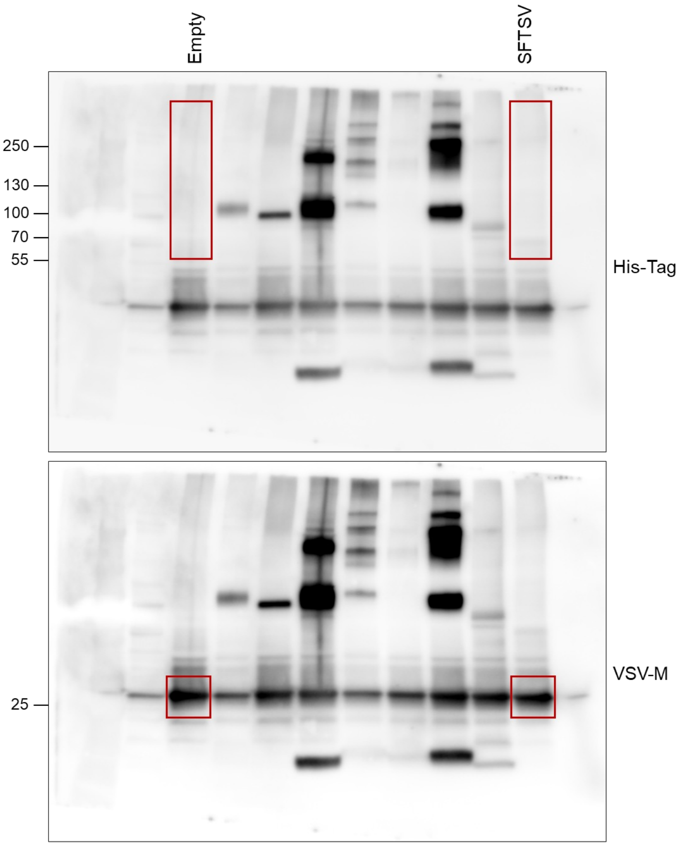

Cell lysates

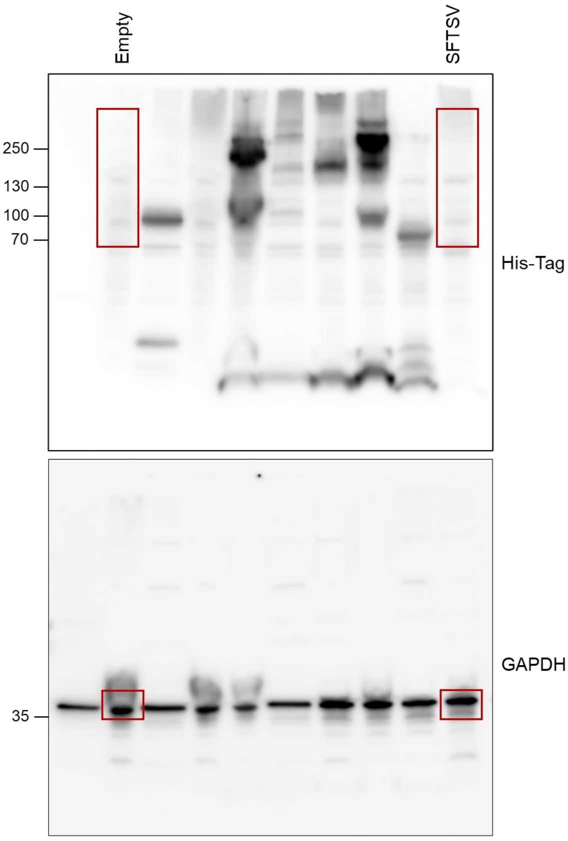

Uncropped blots – Supplementary Figure 10B - 2

Viral particles

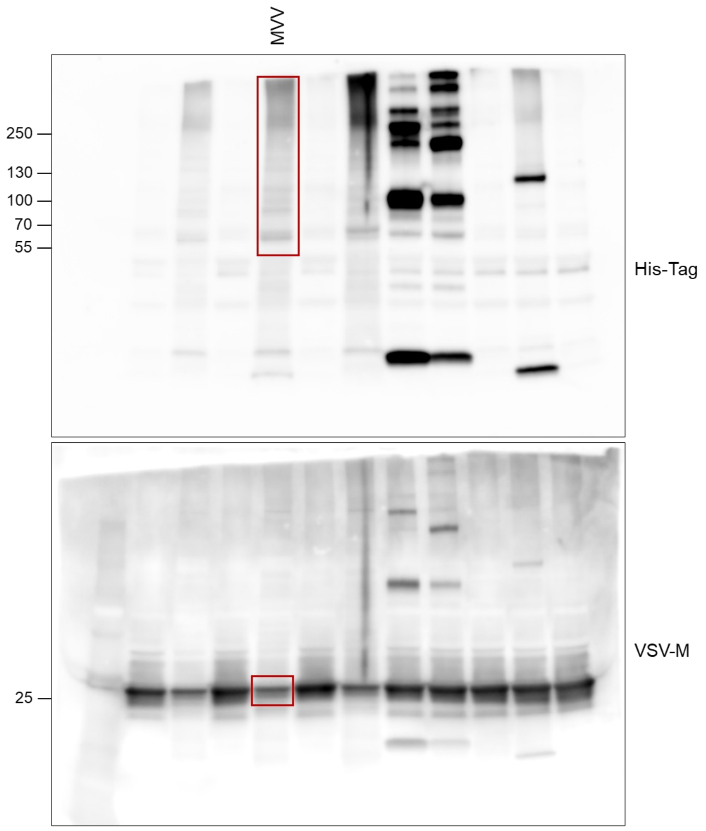

Uncropped blots – Supplementary Figure 11B

Viral particles

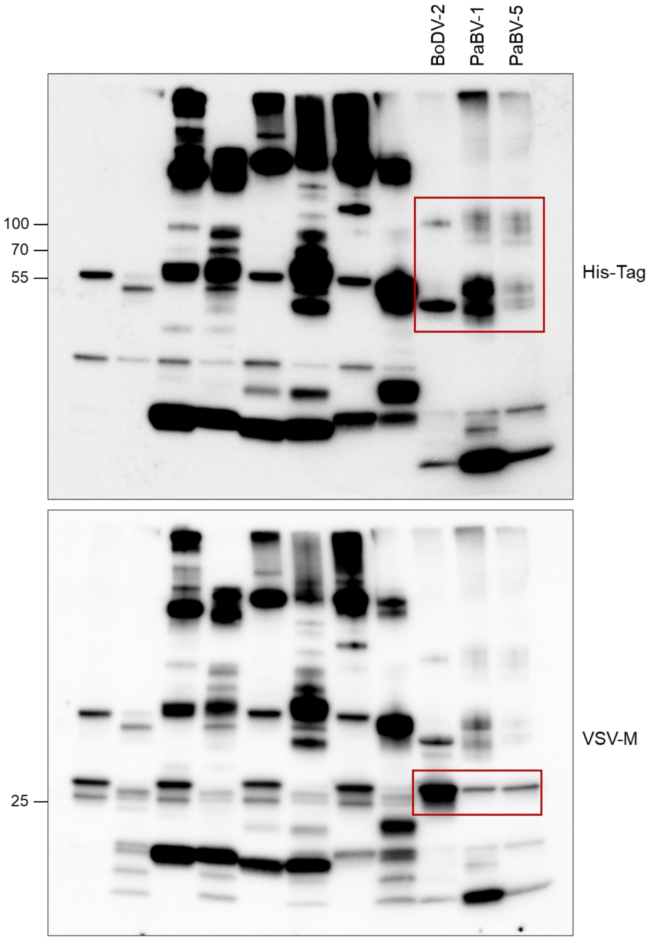

Cell lysates

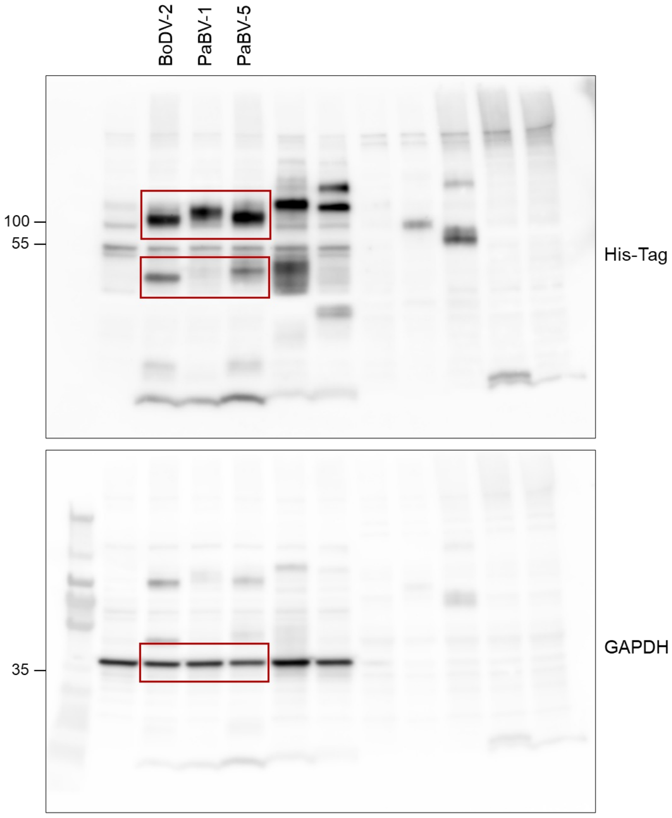

Uncropped blots – Supplementary Figure 12B

Viral particles

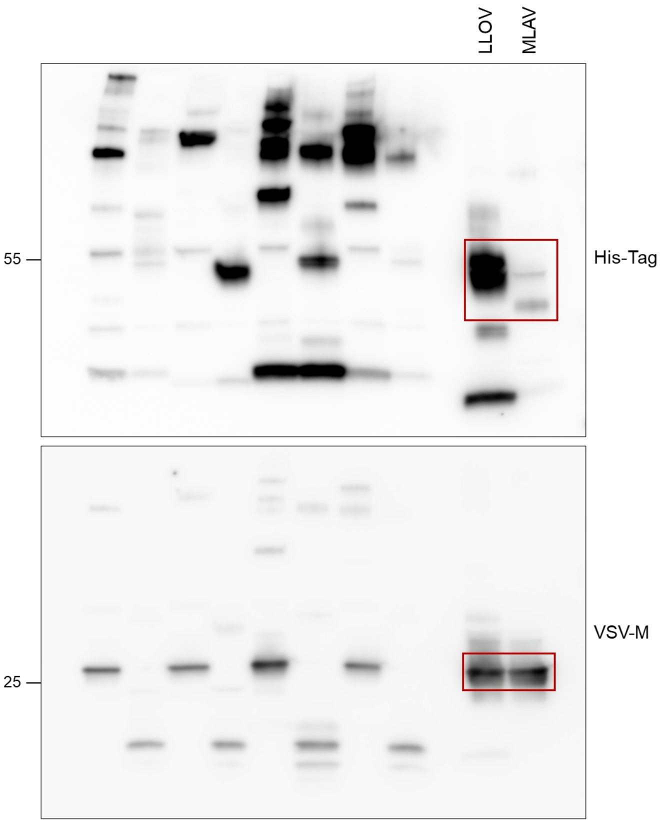

Cell lysates

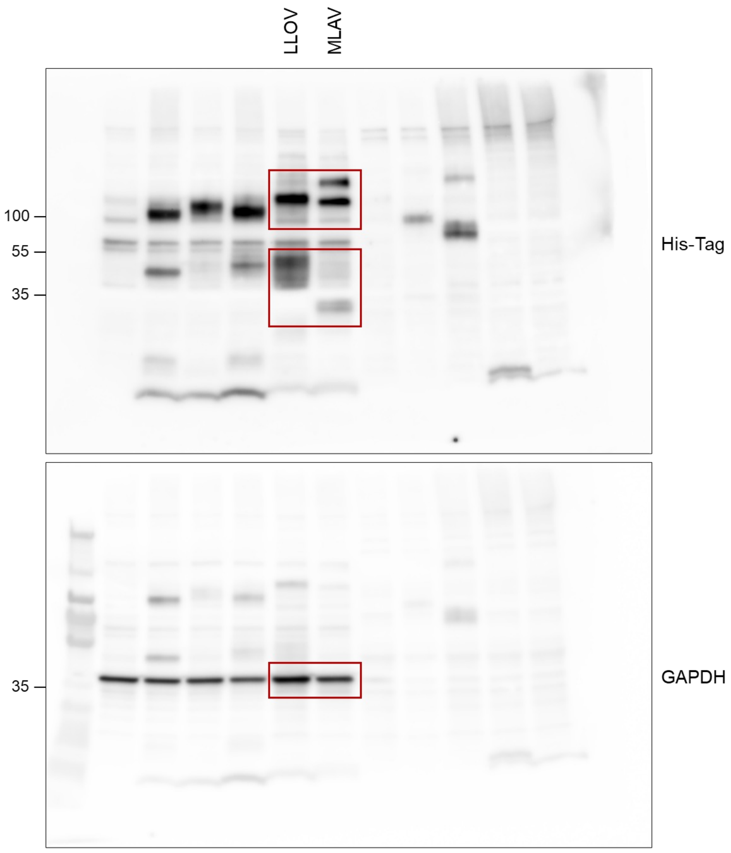

Uncropped blots – Supplementary Figure 13B - 1

Viral particles

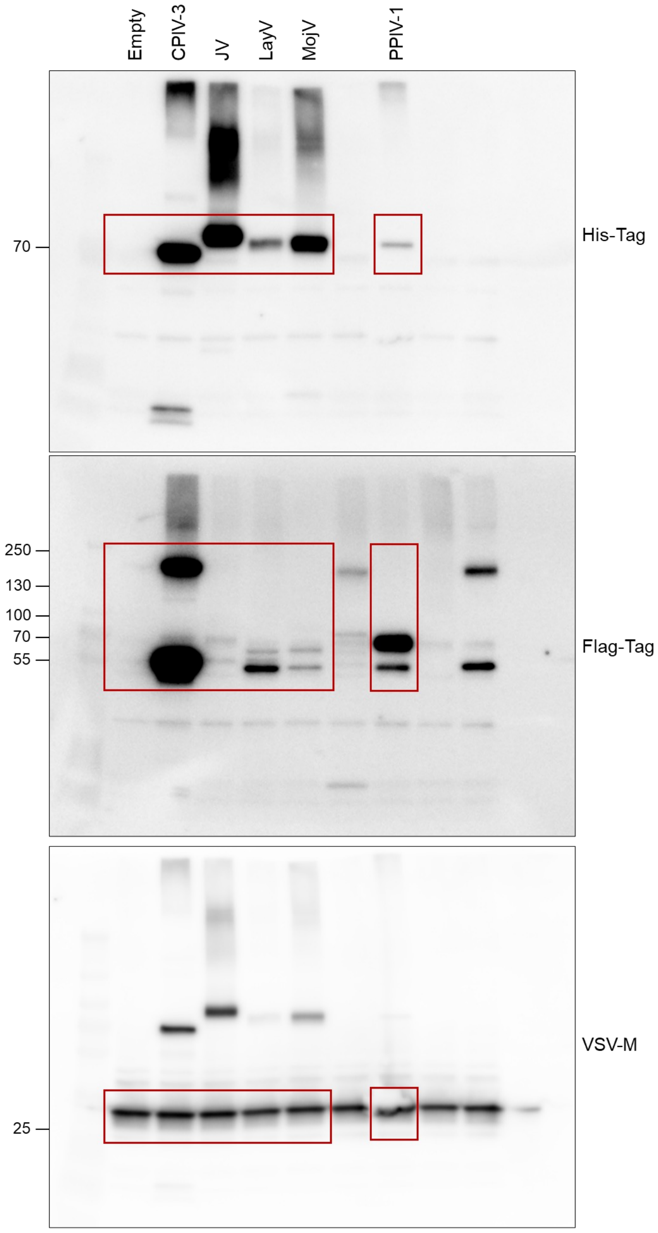

Cell lysates

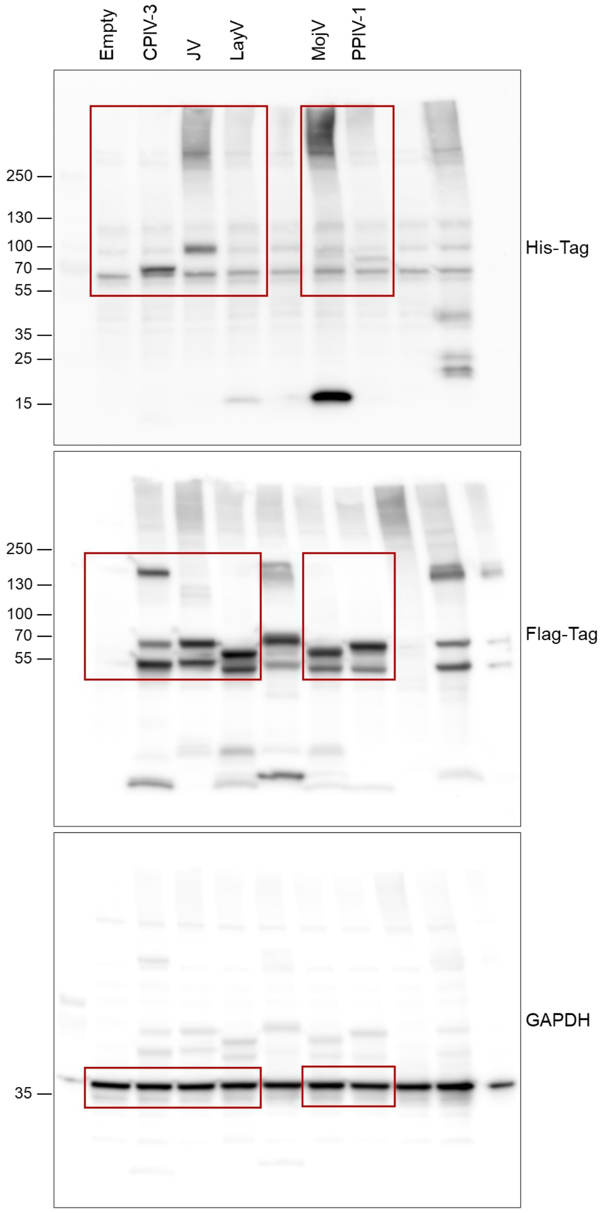

Uncropped blots – Supplementary Figure 13B - 2

Viral particles

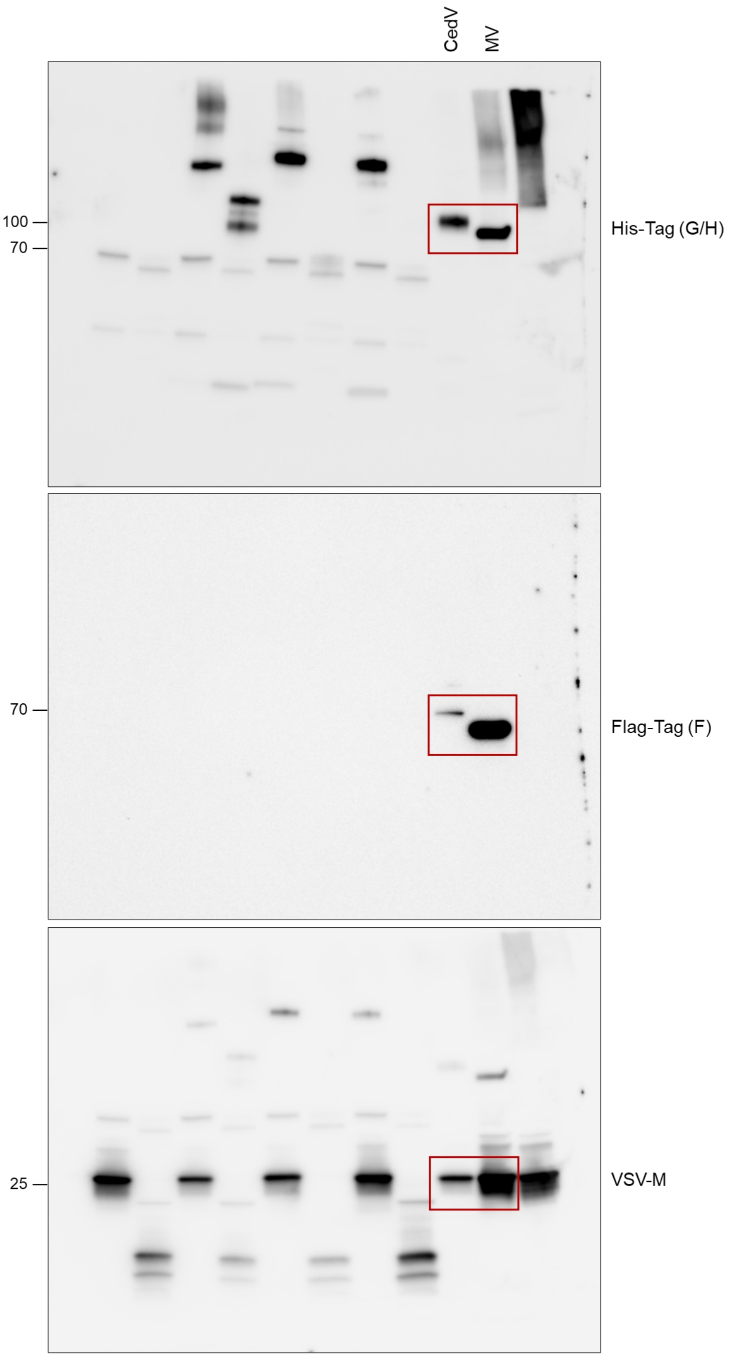

Cell lysates

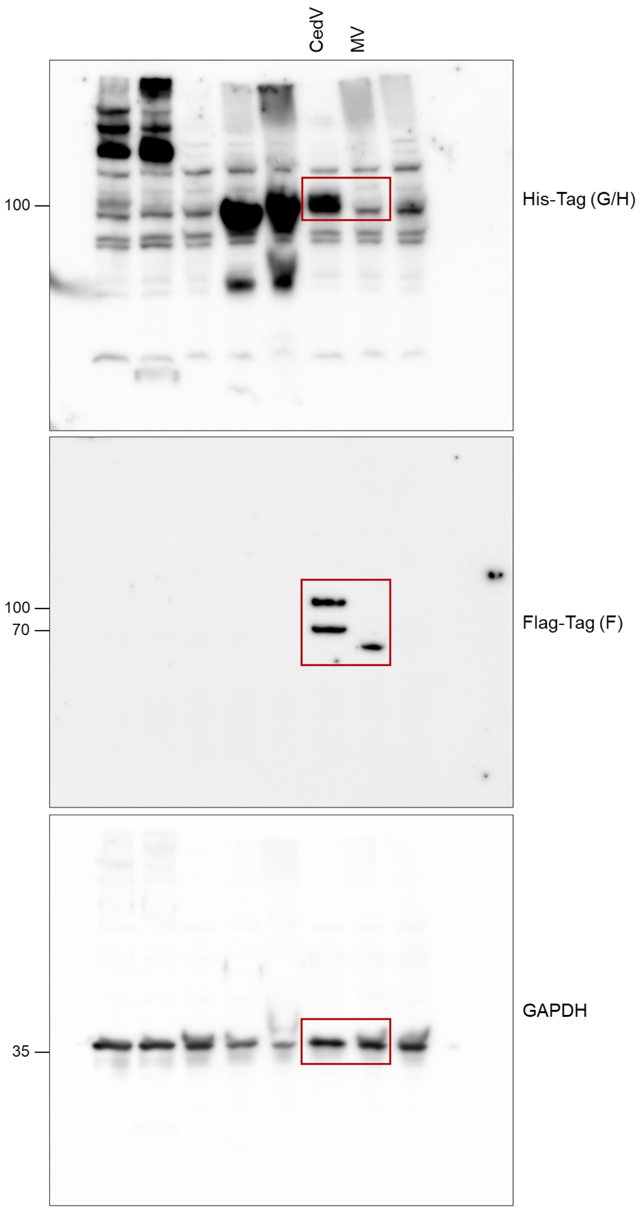

Uncropped blots – Supplementary Figure 14B - 1

Viral particles

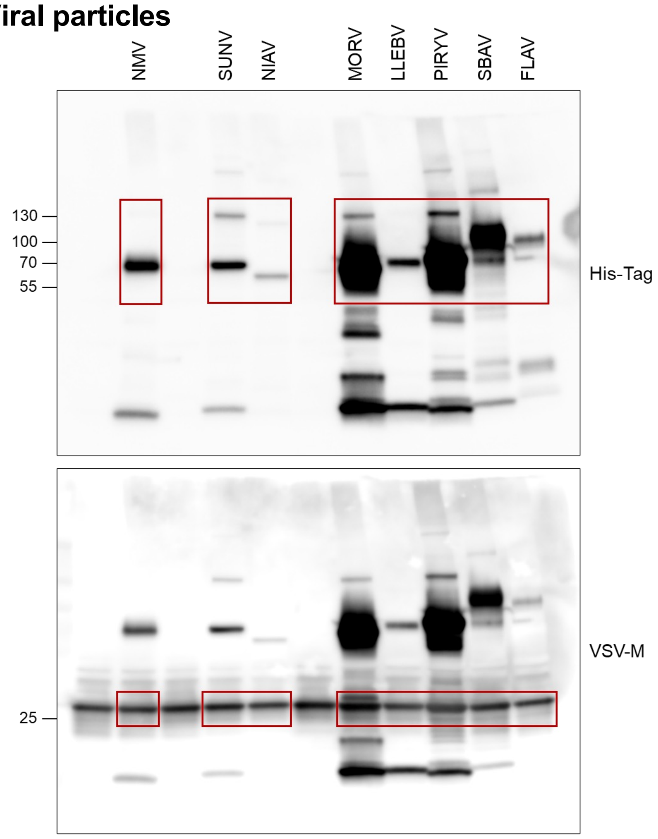

Cell lysates

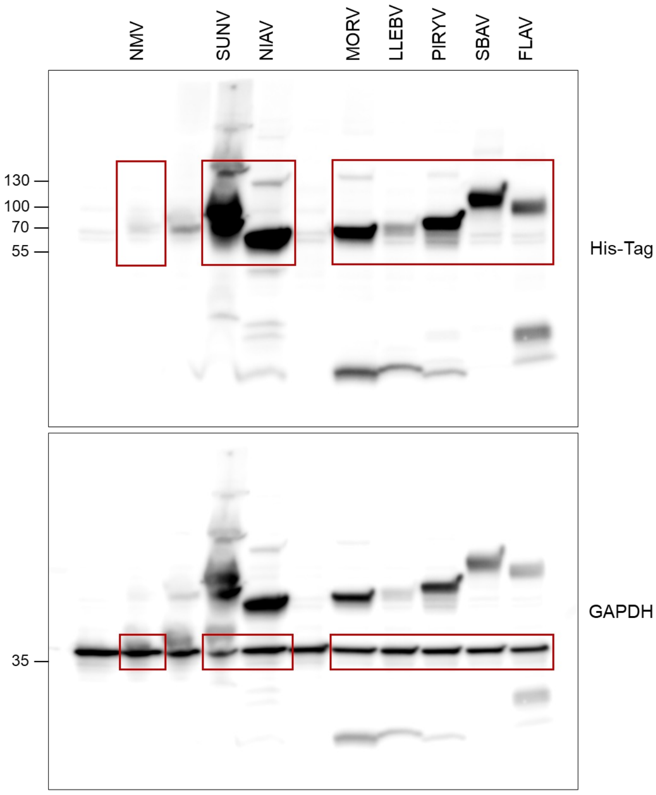

Viral particles

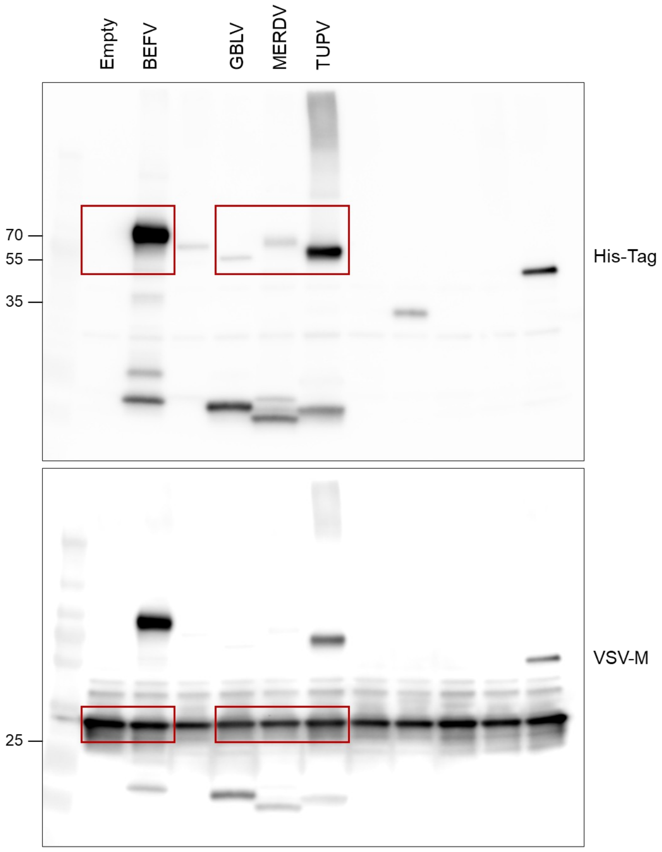

Cell lysates

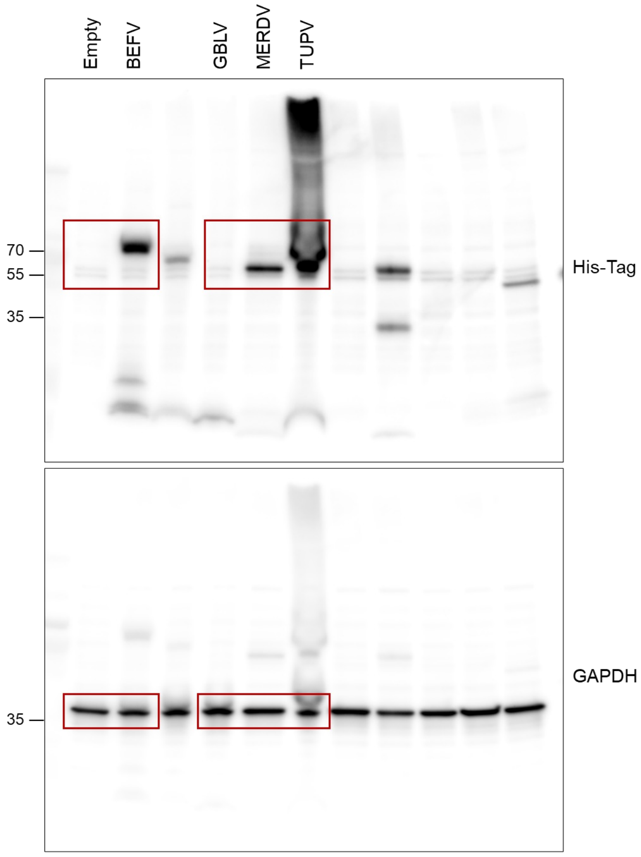

Uncropped blots – Supplementary Figure 14B - 2

Viral particles

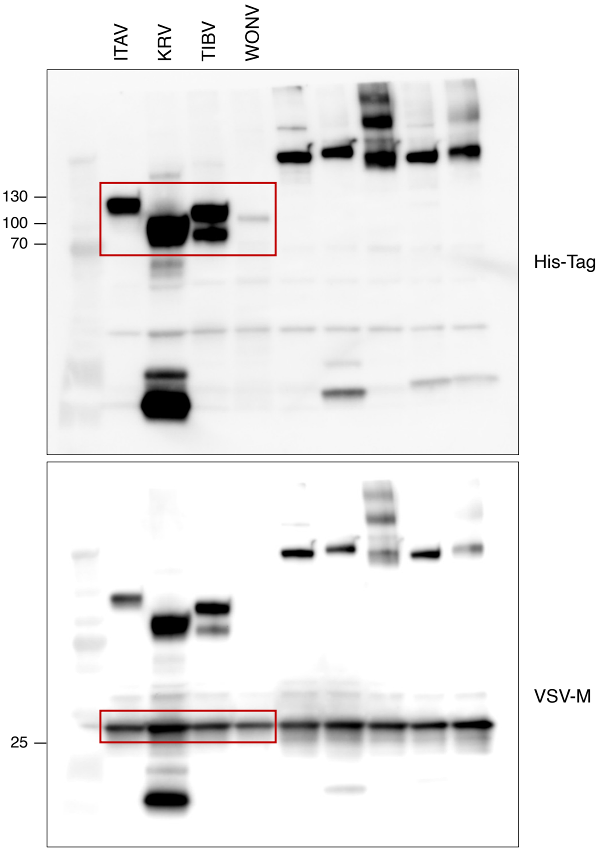

Cell lysates

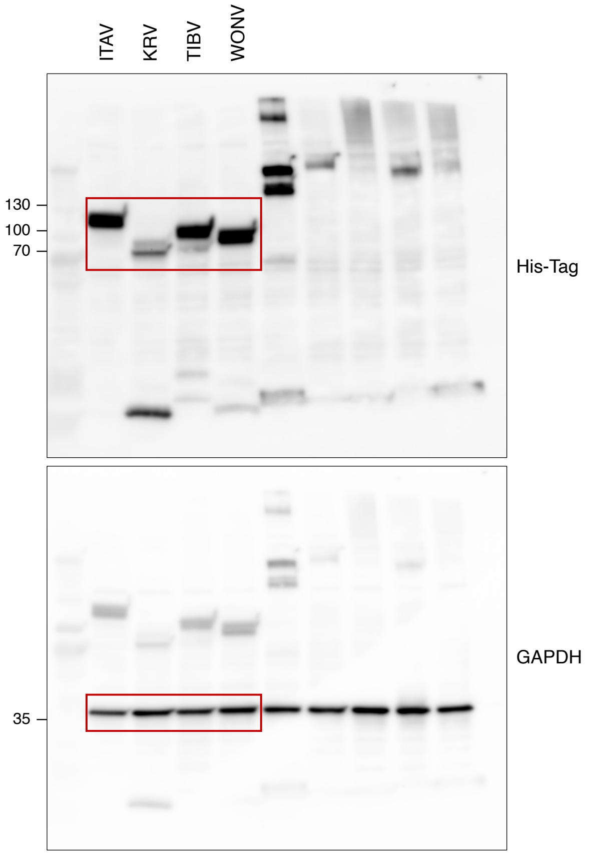

Viral particles

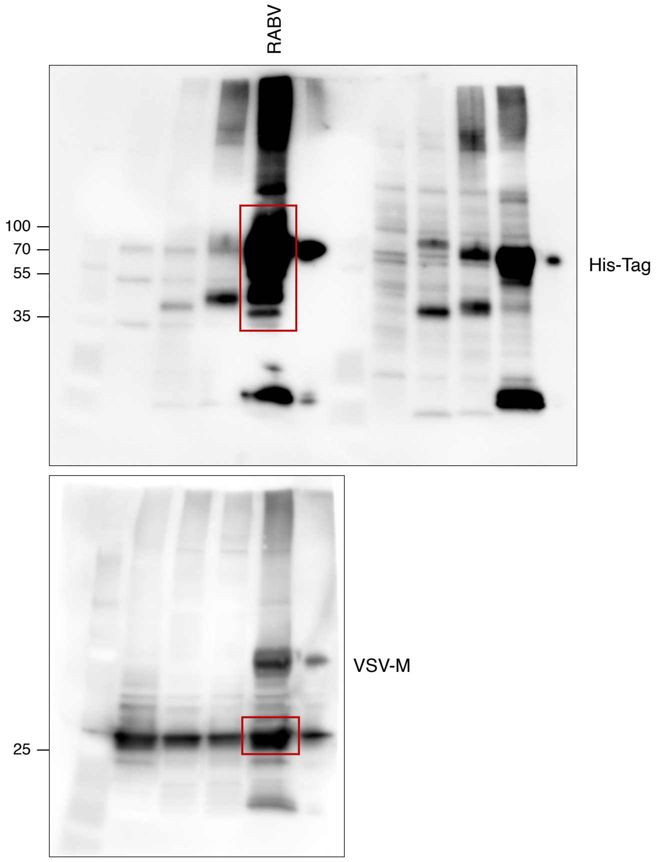

Cell lysates

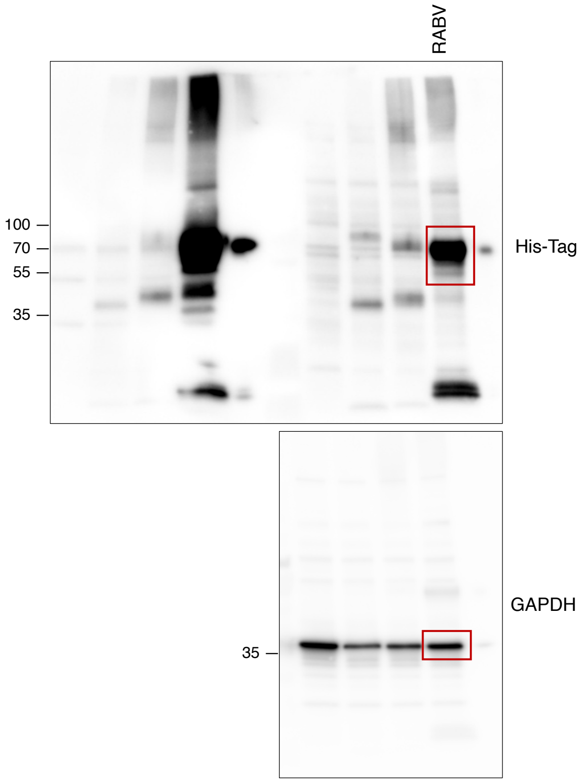

Uncropped blots – Supplementary Figure 14B - 3

Viral particles

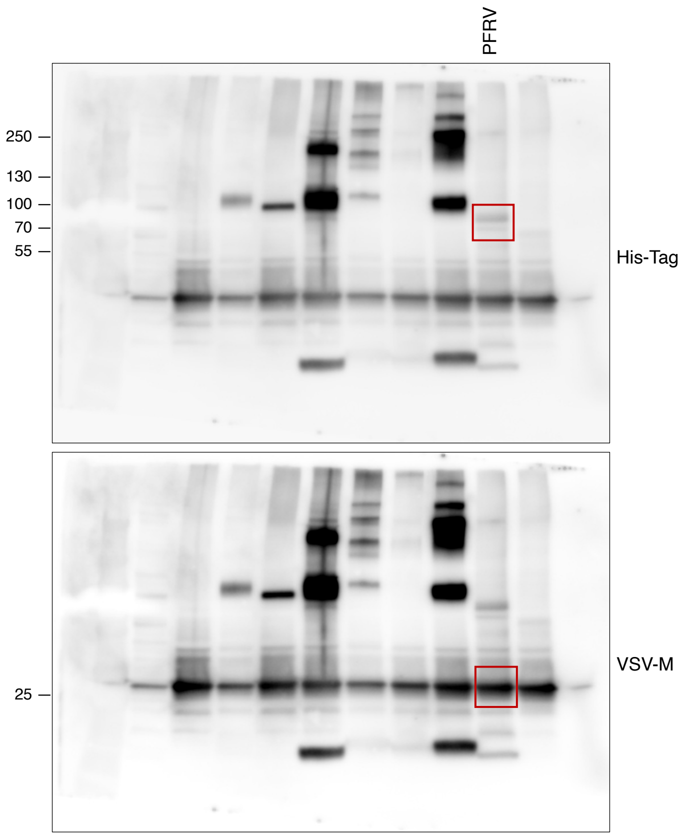

Cell lysates

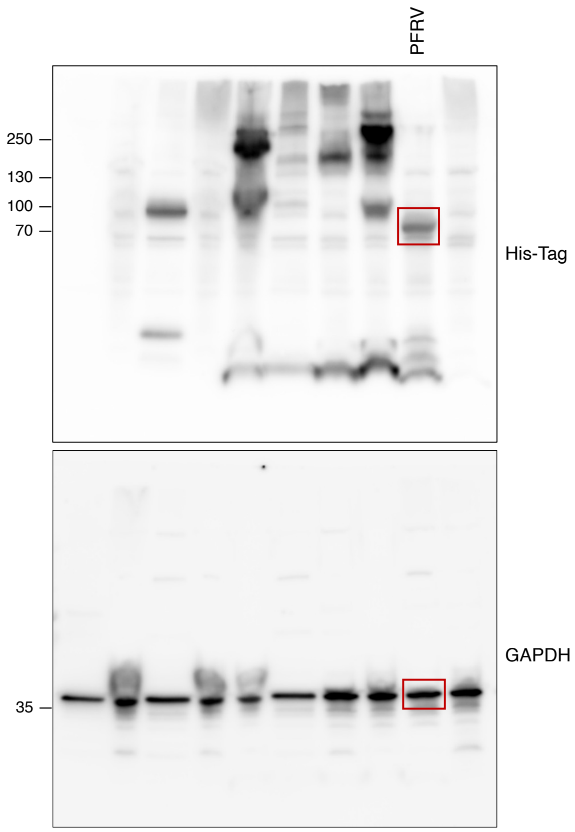

Supplement: Supplementary file 1 — Supplementary Figs. 1–24, legends of Tables 1–6 and Uncropped western blots related to Figs. 1–14. [file 41564_2024_1879_MOESM1_ESM.pdf]
