# Supplementary material for: Receptor-binding proteins from animal viruses are broadly compatible with human cell entry factors
Source: Nat Microbiol. 2025 Jan 2;10(2):405–19. doi: 10.1038/s41564-024-01879-4 (PMC11790484; doi:10.1038/s41564-024-01879-4)
Supplement: Supplementary file 2 — Reporting Summary [file 41564_2024_1879_MOESM2_ESM.pdf]

nature portfolio

Corresponding author(s): Rafael Sanjuán
Last updated by author(s): Nov 4, 2024

Reporting Summary

Nature Portfolio wishes to improve the reproducibility of the work that we publish. This form provides structure for consistency and transparency in reporting. For further information on Nature Portfolio policies, see our Editorial Policies and the Editorial Policy Checklist.

Statistics

For all statistical analyses, confirm that the following items are present in the figure legend, table legend, main text, or Methods section.

n/a	Confirmed	
☐	☒	The exact sample size (*n*) for each experimental group/condition, given as a discrete number and unit of measurement
☐	☒	A statement on whether measurements were taken from distinct samples or whether the same sample was measured repeatedly
☐	☒	The statistical test(s) used AND whether they are one- or two-sided<br>*Only common tests should be described solely by name; describe more complex techniques in the Methods section.*
☒	☐	A description of all covariates tested
☒	☐	A description of any assumptions or corrections, such as tests of normality and adjustment for multiple comparisons
☐	☒	A full description of the statistical parameters including central tendency (e.g. means) or other basic estimates (e.g. regression coefficient) AND variation (e.g. standard deviation) or associated estimates of uncertainty (e.g. confidence intervals)
☐	☒	For null hypothesis testing, the test statistic (e.g. *F*, *t*, *r*) with confidence intervals, effect sizes, degrees of freedom and *P* value noted<br>*Give P values as exact values whenever suitable.*
☒	☐	For Bayesian analysis, information on the choice of priors and Markov chain Monte Carlo settings
☒	☐	For hierarchical and complex designs, identification of the appropriate level for tests and full reporting of outcomes
☐	☒	Estimates of effect sizes (e.g. Cohen's *d*, Pearson's *r*), indicating how they were calculated

Our web collection on statistics for biologists contains articles on many of the points above.

Software and code

Policy information about availability of computer code

Data collection	No software or code was used for data collection.
Data analysis	Fiji v2.1.0<br>Microsoft Excel v2312<br>SPSS v28<br>R v4.3<br>ProtTest (v3.2)<br>RAxML (v8)<br>OSIRIS (v2.16)<br>Incucyte Analysis software (v2022B)<br>xgboost R package (v1.7.8.1)<br>ParBayesianOptimization R package (v1.2.6)<br>shapforxgboost R package (v0.1.3)<br>pvclust R package (v2.2.0)<br>LMNglyPred (KCLabMTU/LMNglyPred)<br>LM-OGlcNAc-Site (KCLabMTU/LM-OGlcNAc-Site)

For manuscripts utilizing custom algorithms or software that are central to the research but not yet described in published literature, software must be made available to editors and reviewers. We strongly encourage code deposition in a community repository (e.g. GitHub). See the Nature Portfolio guidelines for submitting code & software for further information.

Data

Policy information about availability of data

All manuscripts must include a data availability statement. This statement should provide the following information, where applicable:

- Accession codes, unique identifiers, or web links for publicly available datasets
- A description of any restrictions on data availability
- For clinical datasets or third party data, please ensure that the statement adheres to our policy

Features of the RBPs analyzed in this study are available in Supplementary Table 1. Information about natural hosts and cell culture passaging of viruses is available in Supplementary Table 2 and Supplementary Table 3, respectively. The infectivity of VSV pseudotypes in HEK293T and HUVEC cells, and of lentiviral pseudotypes in HEK293T cells is provided in Supplementary Table 4. Features of the 51 cell lines of the NCI-60 panel are available in Supplementary Table 5 and at dtp.cancer.gov/discovery_development/nci-60/cell_list.htm. VSV pseudotype infection data in these 51 cell lines are shown in Supplementary Table 6. Information about receptor cloning is available in Supplementary Table 7. NCI-60 omics data are available at discover.nci.nih.gov/cellminer/loadDownload.do, and at ebi.ac.uk/pride/archive/projects/PXD005940 and in Supplementary Tables 8-10. RNA-seq data from the Human Protein Atlas are available at proteinatlas.org/about/download. Known virus-host associations were retrieved from the Virus-Host Database (genome.jp/virushostdb) and the Virion Database (viralemergence.github.io/virion). Source data files are provided with this paper.

Research involving human participants, their data, or biological material

Policy information about studies with human participants or human data. See also policy information about sex, gender (identity/presentation), and sexual orientation and race, ethnicity and racism.

Reporting on sex and gender	NA
Reporting on race, ethnicity, or other socially relevant groupings	NA
Population characteristics	NA
Recruitment	NA
Ethics oversight	NA

Note that full information on the approval of the study protocol must also be provided in the manuscript.

Field-specific reporting

Please select the one below that is the best fit for your research. If you are not sure, read the appropriate sections before making your selection.

☒ Life sciences ☐ Behavioural & social sciences ☐ Ecological, evolutionary & environmental sciences

For a reference copy of the document with all sections, see nature.com/documents/nr-reporting-summary-flat.pdf

Life sciences study design

All studies must disclose on these points even when the disclosure is negative.

Sample size	By calculating the number of pseudotypes infecting at least one cell line as a function of the number of cell lines considered, we confirmed that including 51 human cell lines in our analysis was a sufficient sample size to detect human-tropic viral receptor-binding proteins.
Data exclusions	No data were excluded.
Replication	All pseudotype infections of the NCI-60 panel were performed in independent in experiments performed on two different days, as indicated in the main text. We always included the same positive control in all infection experiment to validate the robustness and the repeatability of our assay.<br>Western Blot confirmations of RBP incorporation into VSV pseudotypes or cleavage by trypsin were performed once.<br>Infection of HEK293T and HUVEC cells with the 102 VSV pseudotypes was performed once.<br>Infection of HEK293T cells with the 102 lentiviral pseudotypes was performed once.<br>Immunofluorescence validation of host gene overexpression was performed twice independently.<br>The effect of host gene overexpression on pseudotypes infectivity (receptors, IFITM) was performed twice independently.<br>The role of sialic acids in pseudotyped viruses entry was evaluated in two technical replicates.<br>The role of heparan sulfate cleavage following Heparinase III treatment was evaluated in two independent experiments.<br>The validation of sialic acid depletion using IAV and heparan sulfate cleavage by immunofluorescence was performed in two technical replicates and two independent experiments, respectively.<br>All details on repeatability can be found in the figure legends.<br>All attempts of replicating the results were successful.

Randomization	Randomization was not applied to this study because it did not involve research subjects or participants. The study used in vitro assays that typically do not require randomization. However, we ensured reproducibility and validity of the results by enforcing experimental rigor and the use of solid controls and standardized protocols.
Blinding	Biological analysis did not require any subjective analysis and thus did not require blinding.

Reporting for specific materials, systems and methods

We require information from authors about some types of materials, experimental systems and methods used in many studies. Here, indicate whether each material, system or method listed is relevant to your study. If you are not sure if a list item applies to your research, read the appropriate section before selecting a response.

Materials & experimental systems

n/a	Involved in the study	
☐	☒	Antibodies
☐	☒	Eukaryotic cell lines
☒	☐	Palaeontology and archaeology
☒	☐	Animals and other organisms
☒	☐	Clinical data
☒	☐	Dual use research of concern
☒	☐	Plants

Methods

n/a	Involved in the study	
☒	☐	ChIP-seq
☒	☐	Flow cytometry
☒	☐	MRI-based neuroimaging

Antibodies

Antibodies used	Mouse anti-His-Tag (clone HIS.H8, Invitrogen, MA121315)<br>Mouse anti-Flag (clone M2, Sigma-Aldrich, F1804)<br>Mouse anti-VSV-M (clone 23H12, Kerafast EB0011)<br>Rabbit anti-GAPDH (Sigma-Aldrich, ABS16)<br>Mouse anti-digested Heparan Sulfates (Amsbio, F69-3G10)<br>HRP-conjugated anti-mouse (Invitrogen, G-21040)<br>HRP-conjugated anti-rabbit (Invitrogen, G-21234)<br>AF488-conjugated anti-mouse (Invitrogen, A32766)<br>Anti-VSV-G monoclonal antibody (mouse hybridoma cell line AcMI1, as described in PMID 8613674)
Validation	All antibodies used in this study are comercially available and have been validated by the manufacturer, and used by other publications. Especially, all antibodies have been validated for their use in Western Blot and immunofluorescence. Moreover, anti-His and anti-Flag antibodies were validated by checking the absence of signal by western blot or immunofluorescence in bald pseudotypes or cells transfected with an empty plasmid. Similarly, the anti-digested Heparan Sulfates was validated by detecting specific immunofluorescence signal after heparinase treatment of cells. The anti-VSV-G antibody was validated by measuring its neutralizing activity against VSV. The VSV-M and GAPDH antibodies were validated commercially by detecting bands of the correct size in VSV-transfected cells and virions, or various human cell lines, respectively.

Eukaryotic cell lines

Policy information about cell lines and Sex and Gender in Research

Cell line source(s)	The NCI-60 cell line panel was obtained from the National Cancer Institute. HEK293T cells were obtained from the Servicio Central de Soporte a la Investigación Experimental (SCSIE) from the University of Valencia.
Authentication	The 48 adherent cell lines of the NCI-60 panel used in this study were authentificated by Short Tandem Repeat (STR) genotyping. More technical information can be found in the methods section of the manuscript. The three myeloid NCI-60 cell lines (K562, MOLT-4, CCRF-CEM) and HEK293T cells were not authenticated.
Mycoplasma contamination	Cell lines were regularly (every two weeks) shown to be free of mycoplasma contamination by PCR.
Commonly misidentified lines<br>(See ICLAC register)	None

Plants

Seed stocks	NA
Novel plant genotypes	NA
Authentication	NA
