# Supplementary figures and images for: Receptor-binding proteins from animal viruses are broadly compatible with human cell entry factors
Source: Nat Microbiol. 2025 Jan 2;10(2):405–19. doi: 10.1038/s41564-024-01879-4 (PMC11790484; doi:10.1038/s41564-024-01879-4)

Uncropped blots – Extended Data Figure 5B

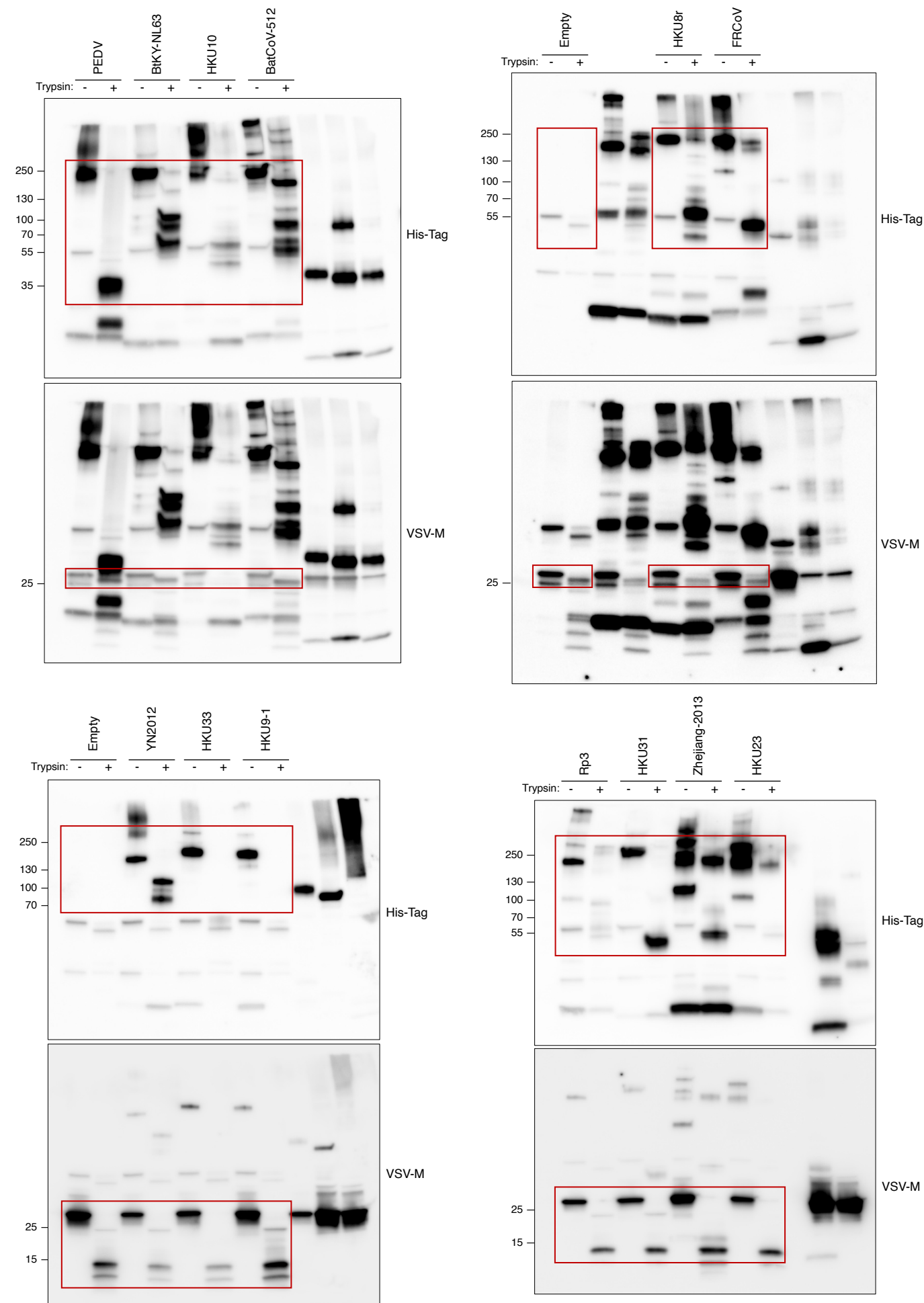

Supplement: Supplementary file 13 — Unprocessed western blots. [file 41564_2024_1879_MOESM13_ESM.pdf]
